# A Systematic Analysis of Lipid–Protein Interactions in the Protein Data Bank

**DOI:** 10.1021/acs.biochem.6c00170

**Published:** 2026-06-18

**Authors:** Nandita Puri, Andrew C. McShan

**Affiliations:** School of Chemistry and Biochemistry, 1372 Georgia Institute of Technology, Atlanta, Georgia 30332, United States

## Abstract

Lipid–protein interactions are ubiquitous in biology, where they are fundamental to membrane structure, cell signaling, immunology, and metabolism. Despite the availability of thousands of experimentally determined lipid–protein structures, the molecular basis for lipid recognition and specificity across the lipid–protein interactome remains incompletely understood. Here, we report a systematic analysis of 113,782 annular and nonannular lipid–protein complexes spanning the eight lipid classes. Pairwise atomic interactions are linked to lipid and protein physicochemical properties and binding geometries. Hydrophobic contacts, hydrogen bonds, and salt bridges contributed to over 99% of lipid–protein interactions. Lipid class-, protein sublocalization-, protein function-, and protein fold-dependent trends were identified. Protein pockets were finely tuned for lipid size, shape, and polarity: fatty acyls associated with narrow, moderately hydrophobic pockets; saccharolipids and glycerophospholipids bound to larger, polar cavities; and sterols and prenols preferentially occupied compact hydrophobic sites. Global analysis across different protein families identified similarities in interaction profiles, while also highlighting protein-specific recognition adapted to biochemical function. Lipid–protein interaction maps were projected onto lipid structures to uncover conserved and divergent hotspots and coldspots across lipid classes. The heatmaps imply that recognition and specificity are mediated by tailored anchoring of polar head groups and varying interaction with hydrophobic tails. Together, the data establish nature’s principles governing lipid binding, lipid selectivity, and complex stability, and collectively provide a molecular atlas of the lipid–protein interactome. The work enables the elucidation of lipid biology at scale and establishes guiding principles for the rational design of chemical probes and therapeutics targeting lipid biology.

## Graphical abstract

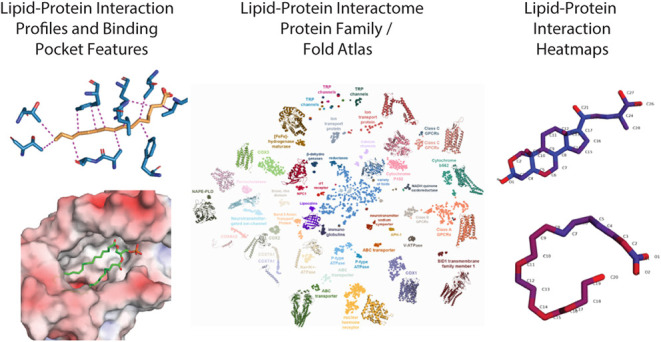

Lipid–protein interactions are universal to biology where they orchestrate processes ranging from membrane structure and energy metabolism to signaling, immune regulation, enzyme cofactors/vitamins, pigments, and other key cellular regulators.^1–4^ Deciphering the landscape of lipid–protein interactions is essential for understanding basic biochemical function, but also for harnessing the intrinsic properties of lipids for biotechnology and therapeutic applications. Dysregulation of lipid–protein interactions due to mutation gives rise to rare lipid storage disorders, cancer, autoimmune disease, heart disease, and neurodegenerative disease.^5–9^ For example, the G863V missense mutation in microsomal triglyceride transfer protein reduces lipid transfer activity by impairing its ability to interact with triglycerides and phospholipids, which contributes to the progression of abetalipoproteinemia.^10^ In addition, lipid–protein interactions have emerged as important tools for biotechnology and medicine, highlighted by lipid nanoparticle-based drug delivery, lipid vaccine adjuvants, and therapeutic modulation of lipid–protein interactions.^11–13^ For example, the glycosphingolipid α-galactosylceramide serves as a potent vaccine adjuvant by activating the innate immune system through lipid–protein interactions with the CD1d immunoreceptor and invariant natural killer T cell receptors.^11^ Despite landmark studies that have applied high-throughput experimental screens to identify lipid binding proteins,^14–19^ the global landscape of lipid–protein complex structures and the atomic interactions responsible for their assembly, stability, and functional regulation remain to be systematically evaluated.

LIPID MAPS categorizes lipids into eight classes: 1. fatty acyls, 2. glycerolipids, 3. glycerophospholipids, 4. sphingolipids, 5. sterols, 6. prenols, 7. saccharolipids, and 8. polyketides^20–22^ (Figure 1). Lipid–protein interactions encompass a vast and heterogeneous landscape consistent with the structural and functional diversity of lipid and protein molecules.^4,23^ Binding events can occur in a variety of cellular environments; for example, lipids interact with soluble proteins in the cytosol or with integral membrane proteins, peripheral membrane proteins, and lipid-anchored membrane proteins in various organelle membranes.^24,25^ Lipid–protein interactions are broadly classified into bulk, surface (annular), and buried (nonannular) regimes.^26^ Bulk lipids do not directly contact proteins but can influence protein dynamics, structure, and function indirectly by modulating membrane properties, such as fluidity, diffusion, and curvature.^4^ Surface (annular) interactions involve lipids that form a dynamic, ring-like, largely nonspecific heterogeneous shell around proteins and are not typically considered biological ligands.^27^ Examples of protein families where annular lipid–protein interactions play key roles include G protein-coupled receptors, ABC transporters, gated ion channels, aquaporins, and light harvesting complexes^28–30^ (Figure S1A). Although annular lipids can modulate protein conformation and function, the interactions are generally transient and solvent-like in nature.^31^ In stark contrast, buried (nonannular) lipid–protein interactions involve lipid binding to well-defined, buried or partially buried protein pockets that confer molecular specificity and functional relevance.^32–34^ The interactions are often characterized by geometric complementarity, headgroup and acyl tail chain-specific contacts, and electrostatic interactions. Evidence that supports nonannular lipid–protein interactions in solution includes NMR spectroscopy, surface plasmon resonance, fluorescence polarization, native isoelectric focusing, isothermal titration calorimetry, thin-layer chromatography, mass spectrometry, and many others.^18,35–38^ Nonannular interactions also typically exhibit higher affinities (nM to μM) and slower kinetic off rates than annular lipids.^33,39^ Nonannular lipid binding is central to recognition of lipids as biological ligands across diverse protein classes; it underpins a range of cellular processes including lipid antigen presentation, lipid transport proteins, lipid transfer proteins, signaling receptors, nuclear hormone receptors, and enzyme-catalyzed lipid metabolism^17,38,40^ (Figure S1B).

**Figure 1:**
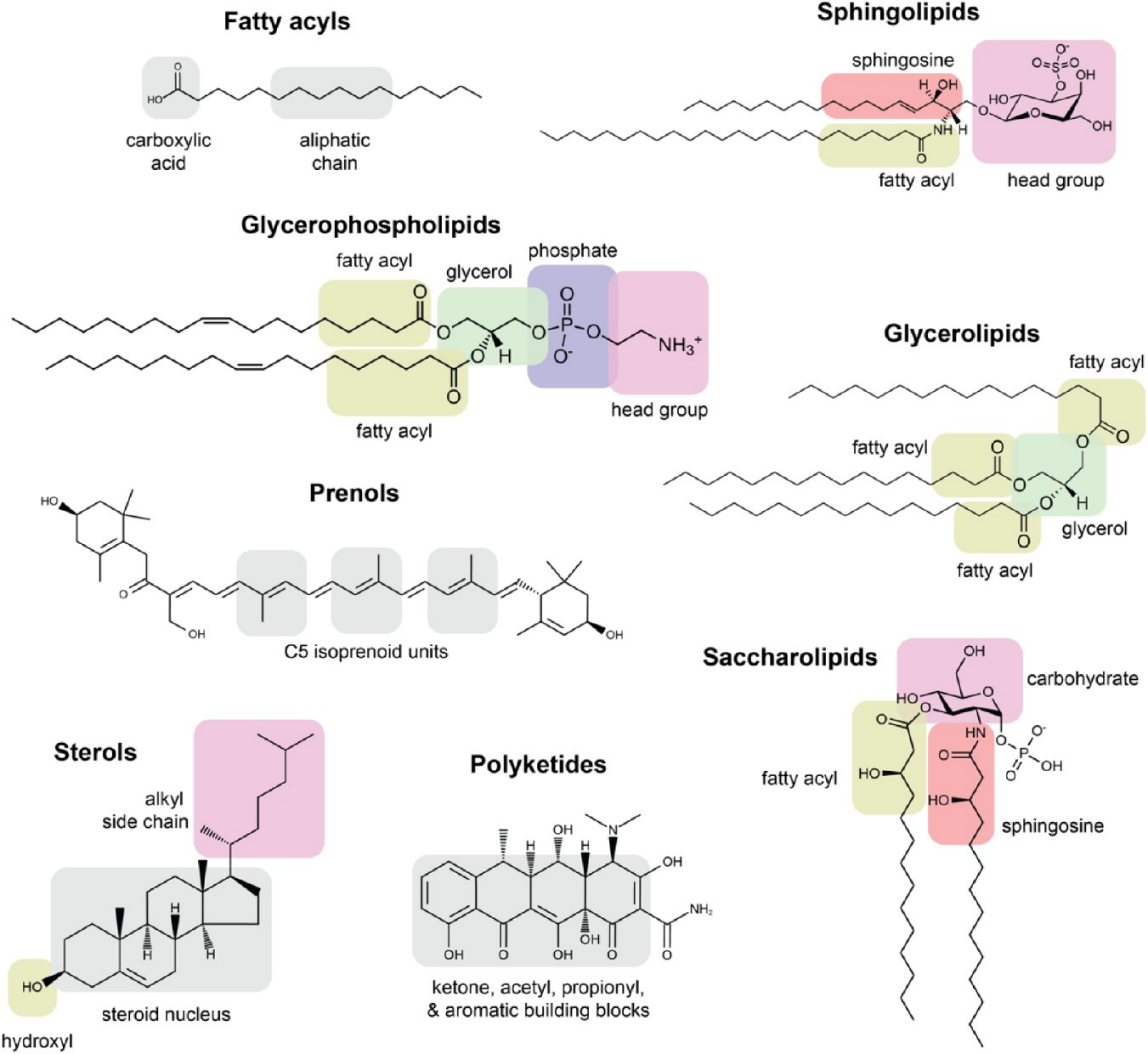
Representative structures of the eight classes of lipids as defined by LIPID MAPS. Example chemical structures for the eight lipid classes as defined by LIPID MAPS.^20–22^ Fatty acyls: palmitic acid 16:0, PubChem CID 135369651; Sphingolipids: sulfatide Δ18:1/24:0, PubChem CID 145710102; Sterols: cholesterol, PubChem CID 5997; Polyketides: doxycycline, PubChem CID 54685920; Prenols: siphonaxanthin, PubChem CID 11204185; Glycerophospholipids: phosphatidylethanolamine 18:1, PubChem CID 9546757; Glycerolipid: tripalmitin, PubChem CID 11147; Saccharolipids: lipid X, PubChem CID 123907. The colored boxes highlight representative examples of specialized chemical units and functional groups relevant to each lipid class.

Curated databases have been developed to study the lipid–protein interactome at scale. DBLiPro is a database for lipid–protein interactions involved in human lipid metabolism.^41^ BioLiP2 contains detailed binding information and affinities for a wide variety of ligand-protein interactions, including lipid–protein complexes.^42^ MemProtMD is a database of membrane protein structures inserted into simulated lipid bilayers.^43^ The Protein Data Bank (PDB) contains atomic structures of lipid–protein complexes solved by nuclear magnetic resonance, X-ray crystallography, or cryo-electron microscopy.^44^ Our group recently developed BioDolphin (Biological Database of Lipid–Protein Highly Inclusive Interactions) as a comprehensive, curated lipid–protein complex structure and interaction database sourced from the PDB, BioLiP2, and others.^45^ Side by side with structural biology and data curation advances, *in silico* tools have been developed to characterize atomic details of ligand–protein interactions and compare interaction profiles between different complexes. Examples include Protein–Ligand Interaction Profiler (PLIP) for deciphering pairwise atomistic interactions,^46,47^ dpocket for characterizing pocket descriptors,^48^ and ProteinCartography for building maps of protein structural landscapes.^49^ Finally, several computational tools have also been developed to predict lipid–protein interactions through sequence or structure-based methods, including LIBP-Pred,^50^ iDLB-Pred,^51^ SliPP,^52^ PLiCat,^53^ and CholBindNet.^54^ However, despite these advances, a systematic analysis of the lipid–protein interactome in the PDB has not yet been performed, which limits mechanistic insights into assembly, specificity, stability, cellular function, and druggability of lipid–protein complexes.

Here, we perform a systematic atomic-level analysis of interactions present in lipid–protein complex entries from BioDolphin, which includes both nonannular (buried) and annular (surface) lipid–protein interactions.^45^ A suite of *in silico* tools, including PLIP,^46,47^ dpocket,^48^ and ProteinCartography,^49^ are utilized to extract features from a wide range of experimental lipid–protein complex structures toward elucidating the physicochemical properties of lipid–protein interactions and protein binding pockets that accommodate the eight different classes of lipids. We uncover broad principles underpinning lipid selectivity and recognition that depend on lipid class, lipid chemistry, and protein family. We discover generalizable features for each class of lipids responsible for association with proteins, while also highlighting examples of lipid–protein interactions that deviate from the typical trends. By integrating global interaction patterns at a protein family level with atomic- and pocket-level descriptions of chemical and biophysical features, the work establishes a foundation upon which to understand nature’s molecular rules governing lipid–protein recognition. The results inform basic principles of lipid biology and could enable translational efforts in lipid-based medicine and biotechnology.

## Materials and Methods

### Data Organization and Curation

A data set of 127,359 lipid–protein interactions (1:1 lipid–protein pairs) derived from 14,891 PDB entries was obtained from BioDolphin_v1.1 (PDB cutoff release date September 6, 2024).^45^ All entries represent experimental nuclear magnetic resonance, X-ray crystallography, or cryo-electron microscopy structures. No solely computationally derived models (i.e., from AlphaFold 3) are present. Metadata for each BioDolphin entry (Biodolphin_v1.1.zip file from https://biodolphin.chemistry.gatech.edu/download) was split into separate excel spreadsheets for each lipid class for accessible data sorting and filtering. Legacy PDB or CIF format files for each lipid–protein interaction were obtained via PDB’s API. Atomic coordinate files from 14,891 PDB entries containing either a single lipid–protein chain pair or multiple lipid and protein chains were separated into 1:1 lipid–protein pairs. This is consistent with how data are organized in BioDolphin and facilitates downstream visualization, characterization, and classification of lipid–protein interactions. Importantly, all lipid–protein interactions from PDB entries containing multiple lipids and protein chains/subunits (i.e., complex multicomponent assemblies) were retained and analyzed. The data set includes lipid–protein interactions spanning both annular (surface-bound) and nonannular (buried) lipid–protein pairs. BioDolphin entries that contained duplicate information (i.e., pairs of the same protein and lipid interacting in the exact same orientation) were removed to reduce data set redundancy. PDB files were cleaned by removing heteroatoms not directly linked to the lipid (i.e., water, ions, metals). Although interactions between water and lipid atoms can contribute to lipid coordination, we did not include them in this work. It is not trivial to identify whether the conformations of bound waters in crystallography models represent biologically relevant conformations. Water–lipid and water–protein interactions may be better characterized using molecular dynamics simulations or complementary experimental approaches. Thus, water atoms were removed from the PDB files prior to processing. Likewise, metals, ions (Ca^2+^, Na^2+^, etc.), and cofactors (heme, etc.) were excluded from the analysis because the focus of this study is lipid–protein interactions. Although these species can influence local binding environments, they represent chemically and functionally distinct classes of ligands. In addition, it is often difficult to clarify whether bound ions and metals reflect biologically relevant states or artifacts present in crystallography or purification buffers. As a result, we focused on direct lipid–protein contacts. Metal atoms directly incorporated into lipid structures were retained and considered. The final data set contained 113,782 lipid–protein interaction pairs spanning the eight classes of lipids. Throughout the manuscript, the PDB Chemical Component Dictionary (CCD) IDs for lipids of interest are noted. Detailed information can be obtained from the RCSB website for each CCD ID: for example, the CCD ID for cholesterol is CLR and the metadata is accessible on the page https://www.rcsb.org/ligand/CLR.

### Determination of Molecular Interaction Using PLIP

Information on the molecular interactions between lipid and protein atoms was obtained using the command line version of the Protein–Ligand Interaction Profiler (PLIP) tool v2.4.0 (https://github.com/pharmai/plip).^46,47^ Prior to PLIP analysis, CIF format files were converted to PDB format using GEMMI v0.7.4 with the gemmi convert command.^55^ PDB files containing only lipid and protein atoms were input to PLIP. PLIP was used to extract lipid–protein interactions with default parameters with the command: plip -f $in_dir/*.pdb -x -t -o $out_dir. The default PLIP geometric criteria used for interaction assignment were as follows: hydrophobic contacts were defined using a maximum heavy-atom distance of 4.0 Å; hydrogen bonds were identified using a maximum donor–acceptor distance of 4.1 Å and a minimum donor angle of 100°; π-stacking interactions were defined using a maximum centroid distance of 5.5 Å, with angular deviation limits of 30° and maximum offset of 2.0 Å; π-cation interactions were defined using a maximum distance of 6.0 Å between charged groups and aromatic ring centers; salt bridges were defined using a maximum distance of 5.5 Å between oppositely charged centers; halogen bonds were identified using a maximum distance of 4.0 Å with an optimal acceptor angle of 120° and donor angle of 165° (±30° tolerance); metal coordination interactions were defined using a maximum distance cutoff of 3.0 Å; and water-mediated bridges were defined using distance cutoffs of 2.5 to 4.1 Å and angular constraints between 71° and 140° depending on geometry. Binding site detection was performed using a 7.5 Å cutoff for inclusion of surrounding residues, and aromatic ring detection used a planarity threshold of 5.0°. Additional details on the definitions that PLIP uses for each interaction type can be found here: https://plip-tool.biotec.tu-dresden.de/plip-web/plip/help. In report mode, PLIP outputs a file called report.txt that contains interaction information. PLIP outputs interaction characteristics for the following interaction types: hydrophobic interactions, hydrogen bonds, π-stacking, π-cation, salt bridges, metal complex, and halogen bonds. In-house Python scripts were used to parse the PLIP report.txt files for information, such as interaction distances, angles, atom types involved, protein family identification numbers, etc. PLIP-determined interactions were visualized in PyMOL v3.1.6.1 with the following color codes: hydrophobic interactions magenta dashed line, hydrogen bonds cyan solid line, π-stacking (parallel) green dashed line, π-stacking (perpendicular) green dashed line, π-cation dark green dashed line, halogen bond orange solid line, salt bridge yellow dashed line, and metal complex violet grey90 dashed line.

Heat maps of lipid–protein interactions for representative lipids from each class were produced by a three-step pipeline combining PLIP output parsing, quantitative aggregation, and structural visualization. First, all PLIP report.txt files for the same lipid bound to different proteins (i.e., same CCD ID and different PDB IDs) were parsed by in-house Bash/AWK scripts. PLIP interaction tables were then parsed to extract lipid atom indices participating in interactions, including those in LIGCARBONIDX, LIG_IDX_LIST, DONORIDX, and ACCEPTORIDX fields, capturing hydrophobic contacts, hydrogen bonds, and other types of interactions. Lipid atom indices were then linked to atom names and elements, and total interaction numbers were aggregated per atom across all structures. Second, interaction frequencies were normalized relative to the atom with the highest interaction count. Interaction frequencies were grouped by atom name and expressed as percentages of total interactions to enable comparison between atoms. Spatial heat maps were created using PyMOL v3.1.6.1. Interaction frequencies for each atom were normalized to the highest frequency observed and plotted on a red-to-blue color gradient (red = highest frequency; blue = lowest). The colored stick model of each lipid offered a three-dimensional view of interaction hotspots. Full scripts and example result outputs are provided in the Data Availability statement for a more detailed description of the pipeline.

### Pocket Analysis with Dpocket

The dpocket module from the fpocket package v4.2.2 (https://github.com/Discngine/fpocket)^48^ was used to extract protein pocket descriptors of lipid bound pockets using PDB files containing only lipid and protein atoms as input. Default parameters were used with the command dpocket -f filelist.txt. Default parameters include α-sphere generation with minimum and maximum radii of 3.4 Å and 6.2 Å, respectively, a minimum pocket size threshold of 15 α-spheres, default polar/apolar classification (A = 3), and default clustering using single-linkage clustering with Euclidean distance and a hierarchical cutoff distance of 2.4 Å. No modifications were made to ligand-restricted modes, chain filtering, volume discretization, or energy calculation options. The pocket descriptors were extracted from the explicitly defined pocket (explicitp.txt). Information on the following pocket descriptors was generated: pocket volume (pock_vol), number of α spheres (nb_AS), pocket surface area (surf_vdw), pocket polar surface area (surf_pol_vdw), pocket apolar surface area (surf_apol_vdw), hydrophobicity score (hydrophobicity_score), mean local hydrophobic density (mean_loc_hyd_dens), proportion of apolar α sphere (apol_as_prop), proportion of polar atoms (prop_polar_atm), mean α sphere solvent accessibility (mean_as_solv_acc), α sphere density (as_dens), maximum distance of α spheres (as_max_dst), volume score (volume_score), polarity score (polarity_score), charge score (charge_score), and flexibility (flex). In-house Python scripts were used to parse the dpocket results (see Data Availability statement). For visualization of protein pockets, PDB files were analyzed in PyMOL v3.1.6.1. Electrostatic surface potential maps were determined in PyMOL with the Adaptive Poisson–Boltzmann Solver (APBS) plugin.^56^ Structures were processed using the PDB 2PQR method.^57^ APBS maps were calculated with 0.5 grid spacing, and contour scaling in the Connolly surface method ranged from −5 kT/e (red, negative) to +5 kT/e (blue, positive).

### Statistical Significance Calculations

Pairwise differences of lipid–protein interaction features between lipid classes or subcellular localization were assessed using a two-sided Mann–Whitney U test. *p*-values were adjusted for multiple comparisons using the Benjamini–Hochberg procedure (FDR < 0.05) as implemented in statsmodels. Significance is defined as either ns = not significant, * = *p* < 0.05, ** = *p* < 0.01, or *** = *p* < 0.001.

### ProteinCartography

ProteinCartography v0.5.0 was installed from Arcadia Science’s GitHub (https://github.com/Arcadia-Science/ProteinCartography).^49^ PDB files for lipid–protein complexes contained within BioDolphin’s data set (1:1 lipid–protein pairs as described above) were separated by the eight different lipid class. In-house Python scripts were used to generate the uniprot_features.tsv file required to run ProteinCartography. ProteinCartography was run in cluster mode with t-SNE plotting with the command:
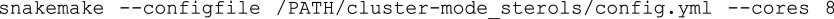


Graph-based clustering was performed on an all-vs-all protein similarity matrix generated using Foldseek.^58^ Foldseek was used to compute all-vs-all structural similarities between proteins, generating a pairwise similarity matrix that served as the input for downstream analyses. By default, Foldseek search masks alignments with e-values > 0.001 and returns up to 1000 hits per query protein. To maintain a complete similarity matrix required for downstream embedding and clustering, masked values (e-value > 0.001) as well as missing entries arising from the top-1000 hit limitation were set to zero. The resulting symmetric similarity matrix was used as input for dimensionality reduction where principal component analysis (PCA) was applied to the full matrix and reduced to 30 components prior to graph construction and downstream embedding. A k-nearest neighbor graph was then constructed from the PCA-reduced space using default parameters from the ProteinCartography pipeline. The number of neighbors was set to n_neighbors = max­(10, round­(N/10)), where N is the number of proteins in the data set, allowing data set-scaled graph construction. Leiden community detection (as implemented in Scanpy) was then applied to the resulting graph using default resolution (1.0), producing data-driven cluster assignments without prespecification of the number of clusters. This enables cluster structure to emerge from the topology of the similarity network. For visualization, t-distributed stochastic neighbor embedding (t-SNE) was applied to the PCA-reduced space using a fixed random seed (random_state = 123456) to ensure reproducibility. t-SNE was run with a perplexity of 50 and 2000 optimization iterations, using default initialization and Euclidean distance as implemented in scikit-learn. These parameters were held constant across all analyses to ensure comparability between data sets. Full scripts and example outputs are provided in the Data Availability statement together with a more detailed description of the pipeline.

## Results

### A Holistic View of Lipid–Protein Interactions

To obtain a holistic view of the lipid–protein interactome, we analyzed 113,782 lipid–protein interactions (1:1 lipid–protein pairs) derived from 14,891 PDB entries from BioDolphin.^45^ The data set contains both annular (surface) and nonannular (buried) lipid–protein interactions (Figure S1A,B). The data set contained 30.4% glycerophospholipids, 26.8% fatty acyls, 14.8% prenols, 7% glycerolipids, 6% sterols, 2.3% polyketides, 0.3% saccharolipids, and 0.2% sphingolipids with the remaining complexes assigned to mixed classifications (Figure 2A). A multitiered framework was implemented to obtain a comprehensive view of global lipid–protein interaction profiles. First, we quantified intermolecular interactions between lipid and protein atoms using the PLIP tool.^46,47^ PDB files for each lipid–protein pair were provided to PLIP, which outputs information on several interaction types (Materials and Methods). Although the analysis is performed on 1:1 lipid–protein pairs for convenience, the full set of lipid–protein interactions from larger multicomponent assemblies is still retained and represented through these decomposed pairwise interactions. The most frequently observed interactions between lipid and protein atoms were hydrophobic contacts (van der Waals forces), hydrogen bonds, and salt bridges (Coulombic electrostatic attraction), which together comprised 99.2% of interactions across all BioDolphin entries (Figure 2B). The remaining 0.8% consisted of π-stacking, π-cation, halogen bonds, and metal-coordinated interactions (Figure 2B). We next compared the count of each interaction type (normalized relative to the most frequently observed) across lipid–protein complexes spanning the eight different lipid classes defined by LIPID MAPS.^20–22^ Hydrophobic contacts, hydrogen bonds, and salt bridges were consistently represented between lipid and protein atoms across all eight lipid classes, albeit to varying degrees (Figure 2C). Lipid class-specific trends emerged (Figure 2C,D). Some notable examples are (1) prenol–protein interactions displayed much fewer salt bridges than glycerophospholipid–protein interactions, consistent with the predominantly un-ionized, aliphatic isoprenoid-derived moieties of prenols relative to the ionized anionic phosphate groups in the backbone of glycerophospholipids, (2) saccharolipid–protein interactions were enriched with hydrogen bonds relative to prenol–protein interactions, reflecting the elevated presence of carbohydrate hydroxyl and ether functional groups in saccharolipid structures, and (3) polyketide–protein interactions contained an increased amount of π-stacking interactions relative to fatty acyl-protein interactions, likely reflecting the presence of aromatic rings in polyketide structures. Together, the global PLIP analysis supports that most lipid–protein interactions are driven by hydrophobic interactions. In addition, hydrogen bonding and salt bridges are also common interactions observed. An important observation, not frequently discussed in the literature, is that lipid–protein interaction profiles are highly lipid class-specific due to the presence or absence of specialized subunits and functional groups (Figure 1).

**Figure 2:**
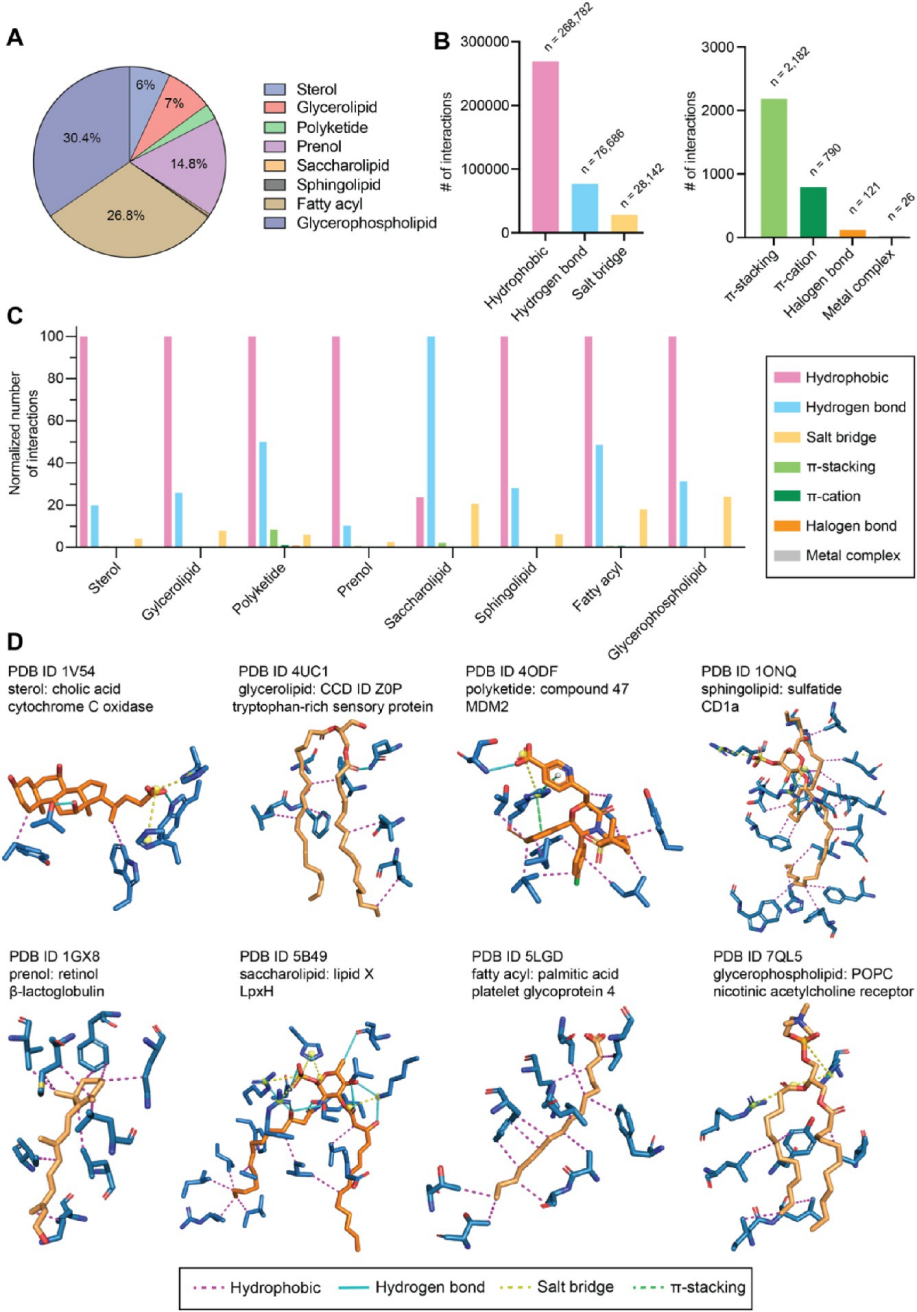
Distribution of molecular interactions across lipid–protein complexes in the Protein Data Bank. (A) The percentage of lipid types across lipid–protein complexes in the curated BioDolphin data set. (B) Total interaction count for the most common (left) and least common (right) molecular interactions determined using the PLIP tool. The n value represents the total number of interactions observed across all entries for each type of interaction. (C) Normalized number of interactions for lipid–protein complexes across each class of lipid. Interaction counts were normalized by dividing counts of each interaction type by the highest count within each class of lipid separately. For clarity *p*-values are not shown but were calculated and are provided in the associated source data file. (D) Representative examples of PLIP results highlighting interactions between protein atoms (blue sticks) and lipid atoms (orange sticks). The PDB ID, lipid name, and protein name are provided. The color key for the PLIP-determined interactions (shown as dashed or solid lines) is also provided.

To explore features of the data set at a deeper level, the structural completeness of lipids present in PDB entries was evaluated. Lipids may not be fully modeled in crystallography or cryo-EM structures due to low resolution, conformational dynamics, or both.^59^ We found that 60.6% of PDB files contained fully modeled lipid structures, whereas 39.4% contained incompletely modeled lipid structures. This is not surprising since BioDolphin does not filter out structures by resolution or completeness. Of the 2,605 unique lipid CCD IDs analyzed, most were represented by at least some PDB entries with fully modeled atoms. However, 241 lipid CCD IDs lacked any fully modeled structures. This implies a potential bias in interaction profiles for certain lipids arising from either the limited availability of experimental structures and/or unresolved lipid regions, particularly flexible carbon atoms within longer acyl-chain tails. We also assessed whether binding affinities between lipids and proteins in the data set had been reported, which would support the presence of nonannular (buried, biologically relevant) lipid–protein interactions (Figure S1). BioDolphin contains experimental binding information (where available) sourced from BioLiP2, PDBbind, BindingDB, Binding MOAD, and other related sources.^45^ Binding information (K_D_, IC_50_, etc.) was available for 2.3% of lipid–protein complexes in the data set spanning eight major lipid classes. This represents that only a small subset of entries have reported experimental binding affinities. Mean reported equilibrium dissociation constant values (K_d_ ± standard deviation) were: Fatty Acyls 286 ± 1219 μM, Glycerolipids 3.2 ± 0 μM, Glycerophospholipids 31.6 ± 88.2 μM, Polyketides 62.4 ± 606.6 μM, Prenols 82.5 ± 603.4 μM, Saccharolipids 404 ± 305.7 μM, Sphingolipids 0.094 ± 0.176 μM, and Sterols 409 ± 6621 μM. K_d_ values ranged from low nanomolar (sphingolipids) to high micromolar (sterols) affinities, supporting that at least a portion of the analyzed lipid–protein interactions reflect biologically relevant and selective binding rather than incidental molecules present during copurification or biologically irrelevant ligands present due to crystallographic artifacts.

Second, dpocket was used for global evaluation of the chemical and geometric features of lipid binding protein pockets.^48^ PDB files for each lipid–protein pair were provided to dpocket, which output values for a variety of pocket descriptors (Materials and Methods). Lipid class-specific trends emerged in protein pocket volume, lipid volume, lipid volume/pocket volume ratio, pocket hydrophobicity, pocket polarity, and pocket geometry (Figure 3A,B). Protein pockets bound to fatty acyls contained the smallest pocket volumes among all lipid classes with a median volume of 345.2 Å^3^. This likely reflects the predominantly monopodal architecture and malleability of fatty acyls, which typically bind narrow hydrophobic cavities. In stark contrast, protein pockets containing saccharolipids and sphingolipids had the largest pocket volumes with median volumes of 1030.7 and 981.2 Å^3^, respectively. This is consistent with large saccharolipid and sphingolipid polar headgroups and bipodal hydrocarbon chains, which are accommodated by wider binding cavities. Volumes of protein pockets containing other lipid classes fell into an intermediate range spanning 430 to 700 Å^3^. Protein pocket volumes correlated with bound lipid volumes. Fatty acyls had the smallest volumes with a median of 168 Å^3^. On average, glycerophospholipids and sphingolipids had the largest volumes with medians of 510 and 514 Å^3^, respectively. Sterols, polyketides and prenols occupied an intermediate volume range (290 to 475 Å^3^). Lipid volume-to-pocket volume ratios further highlighted differences in packing efficiency: most lipids were equal to or smaller than the occupied protein cavity (ratio of 1 or less). Glycerophospholipids and glycerolipids exhibited the highest median ratios (∼0.91), indicating tight volumetric complementarity between lipid size and protein cavity. In contrast, fatty acyls, saccharolipids, and sphingolipids displayed lower ratios (0.39 to 0.45), consistent with their amphipathic architectures that require both hydrophobic enclosure and polar exposure. Sterols and prenols showed intermediate-to-high ratios (0.67 to 0.73), consistent with their rigid, compact scaffolds.

**Figure 3:**
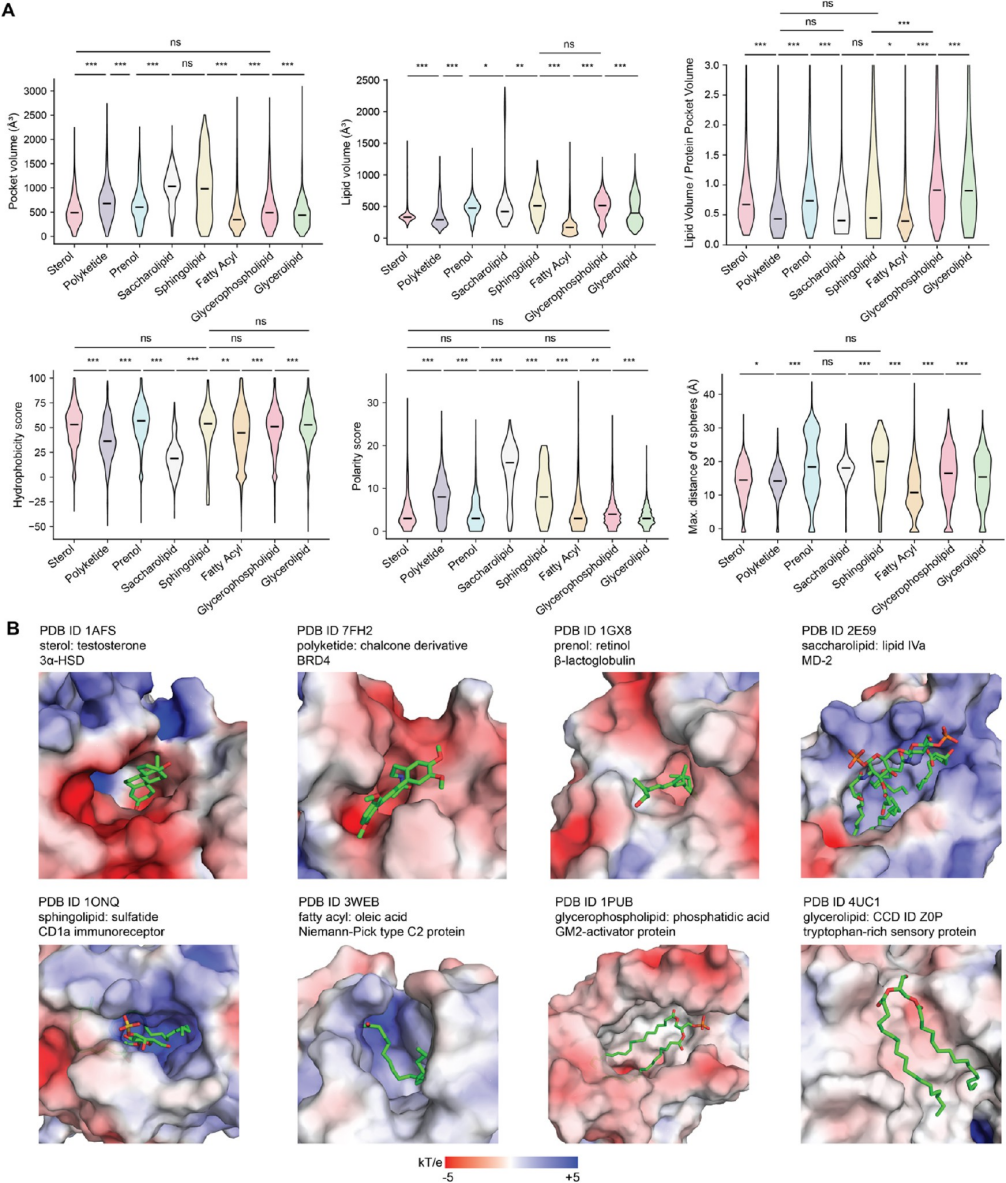
Summary of lipid binding protein pocket features of lipid–protein complexes in BioDolphin. (A) Results from dpocket analysis of protein pockets containing lipids classified within the eight lipid classes. Violin plots of dpocket descriptors are shown with median values represented as horizontal black lines. The number of lipid-bound protein pockets analyzed for each lipid class (n) were: sterols (*n* = 6783), polyketides (*n* = 2576), prenols (*n* = 16586), saccharolipids (*n* = 329), sphingolipids (*n* = 222), fatty acyls (*n* = 28530), glycerophospholipids (*n* = 28752), and glycerolipids (*n* = 7432). Pairwise differences between pocket features between lipid classes were assessed using a two-sided Mann–Whitney U test. *p*-values were adjusted for multiple comparisons using the Benjamini–Hochberg procedure (FDR < 0.05) as implemented in statsmodels. ns = not significant. * = *p* < 0.05; ** = *p* < 0.01; *** = *p* < 0.001. Statistical significance (*, **, or ***) is denoted only for select data sets for clarity. All ns data set comparisons are denoted. (B) Representative examples of lipid bound protein pockets. Proteins are shown in surface view colored by electrostatic surface potential calculated with the Adaptive Poisson–Boltzmann Solver (APBS plugin of PyMOL).^61^ The contour scale for the APBS visualization is −5 kT/e (red, negative) to +5 kT/e (blue, positive). Bound lipids are shown as green sticks.

We evaluated the hydrophobicity of lipid bound protein pockets using dpocket’s hydrophobicity and polarity scores (Figure 3A). Hydrophobicity scores range from −55 to 100 (more polar to more hydrophobic).^60^ Protein pockets containing sterols, prenols, sphingolipids, and glycerophospholipids had higher median hydrophobicity scores (∼51 to 57), indicating predominantly nonpolar binding environments. Protein pockets containing fatty acyls had moderate median hydrophobicity scores (∼45), while those with saccharolipids showed the lowest median hydrophobicity (∼19). Hydrophobicity scores were inversely correlated with polarity scores. We also evaluated pocket geometry using dpocket’s *as_max_dst* descriptor, which reports the maximum distance between alpha spheres to capture pocket geometry and shape. Pockets bound to fatty acyls exhibited the smallest median *as_max_dst* (10.8 Å), suggesting a preference for narrow, elongated cavities (Figure 3A). In contrast, prenol-, sphingolipid-, and saccharolipid-bound pockets displayed the largest median *as_max_dst* values (18.1 to 19.9 Å), indicating a preference for more extended binding sites with larger surface areas. Sterols, polyketides, glycerophospholipids, and glycerolipids occupied intermediate ranges (14.2 to 16.5 Å). These trends parallel volumetric differences and further support that lipid binding protein pocket architectures are shaped by lipid size, chemistry, and topology.

The violin plots support that, even for different lipid–protein complexes within the same class of lipid, protein pocket volume, lipid volume, pocket hydrophobicity, pocket polarity, and *as_max_dst* values span a remarkable range of values. This is fully consistent with the massive diversity in the size, geometry, and chemistry of lipids. Together, the analysis provides compelling evidence that lipid binding protein pockets are finely tuned to the size, geometry, polarity, and hydrophobicity of their cognate ligands (Figure 3B). Small, linear lipids, typical of fatty acyls, occupy compact, moderately hydrophobic pockets to accommodate flexible aliphatic hydrocarbon tail moieties, whereas bulky polar ligands, like saccharolipids, require larger pockets that are more hydrophilic to accommodate hydroxyl containing carbohydrate moieties. Overall, the structural and physicochemical properties of protein pockets closely complement lipid chemistry, indicating that both geometric fit and chemical compatibility govern lipid recognition and complex stability.

### Comparisons of Lipid–Protein Interaction Profiles and Pocket Features across Subcellular Locations

Lipid and protein compositions vary substantially across different cellular compartments. For example, the cytoplasm contains a distinct lipid and protein repertoire compared to the plasma membrane, endoplasmic reticulum, mitochondrion, and lysosome, each of which is enriched in specific lipids.^1^ Similarly, proteins are differentially localized across compartments for functional specialization within certain organelles.^62^ To assess whether biochemical and spatial differences translate into distinct features of the lipid–protein interactome, we reframed the lipid–protein interaction and pocket feature analysis through stratifying results by subcellular localization annotated via Gene Ontology (GO) Cellular Component terms.^63^

We found that interaction profiles of several lipid classes were broadly conserved across different cellular compartments (Figure S2). For example, sphingolipid–protein, prenol–protein, and polyketide–protein interactions showed similar interaction trends independent of subcellular localization. Other lipid classes displayed compartment-specific interaction signatures (Figure S2). A few examples are described below. Sterol–protein interactions within respiratory chain complex IV in the mitochondrial inner membrane were enriched in hydrogen bonds. The cholic acid–cytochrome c oxidase interaction is not merely a crystallographic artifact;^64^ it occurs in solution where it promotes cytochrome c oxidase dimerization and induces a structural change that abolishes the allosteric ATP-mediated inhibition of the enzyme.^65–67^ Saccharolipid–protein interactions at the bacterial flagellum basal body within the outer cell membrane were enriched in van der Waals contacts. Hydrophobic contacts between the L-ring protein and the lipid A portion of lipopolysaccharide guide proper insertion and assembly of the L-ring during flagellum biogenesis, ensuring it localizes in the outer membrane before the full motor assembles.^68^ Glycerophospholipid–protein interactions in plasma membrane-derived thylakoid membranes showed an increased number of hydrogen bonds. Hydrogen bonds between the cytochrome b559 subunit α of photosystem II and phosphatidylglycerol are key to stabilizing the reaction center complex.^69^ They play roles in supporting proper assembly and functional integrity of electron transfer machinery in the thylakoid membrane.^70,71^ We also asked whether pocket features of lipid–protein complexes are conserved across different cellular compartments (Figure S3). Saccharolipid–protein interactions include complexes present in the bacterial flagellum basal body within the outer cell membrane characterized by lower protein pocket volumes and higher lipid volumes, such as those formed between photosystem II subunits and the lipid A component of lipopolysaccharide (as noted above).^68^ Glycerolipid–protein interactions include complexes contained within respiratory chain complex IV in the mitochondrial inner membrane, such as cytochrome c oxidase interacting with tristearin, which are characterized by higher protein pocket volumes and higher lipid volumes.^64^ Together, these results demonstrate that subcellular localization is a contributing determinant of the lipid–protein interactome, driven by both the local availability of lipid species and the specialized functional requirements of protein complexes embedded within distinct cellular environments.

### Comparison of Interaction Types across Different Lipid Classes

#### Hydrophobic Interactions

From the PLIP analysis (Figure 2), hydrophobic contacts were by far the most common interaction type in lipid–protein complexes with a total of 268,782 contacts across the data set. Hydrophobic contacts between lipid and protein atoms were dominated by contributions from amino acids containing aliphatic side chains (Ala, Val, Ile, Leu, Met, Pro) and aromatic rings (Phe, Tyr, Trp) (Figure 4). Apart from saccharolipid–protein complexes, which showed a global reduction in hydrophobic contacts, hydrophobic interactions represented more than 75% of interactions for aliphatic and aromatic amino acid residue types. Charged and polar uncharged amino acids, such as Thr, His, Lys, Asn, and Glu, also contributed to hydrophobic interactions with lipids through aliphatic moieties, albeit to a lesser extent. Hydrophobic contact distances between protein and lipid atoms ranged from ≈3.4 to 4.2 Å, consistent with optimal distances for van der Waals forces and hydrophobic packing of small molecule–protein complexes (Figure S4).^72^ The median distance of hydrophobic interactions between protein and lipid atoms was consistent across all lipid classes at ≈3.7 Å. This analysis largely supports previous claims that many, but not all, lipid–protein interactions are driven by van der Waals forces and the hydrophobic effect.^73,74^ It also broadly uncovers features of the hydrophobic contacts and how they differ across lipid–protein complexes composed of different lipid classes for the first time.

**Figure 4:**
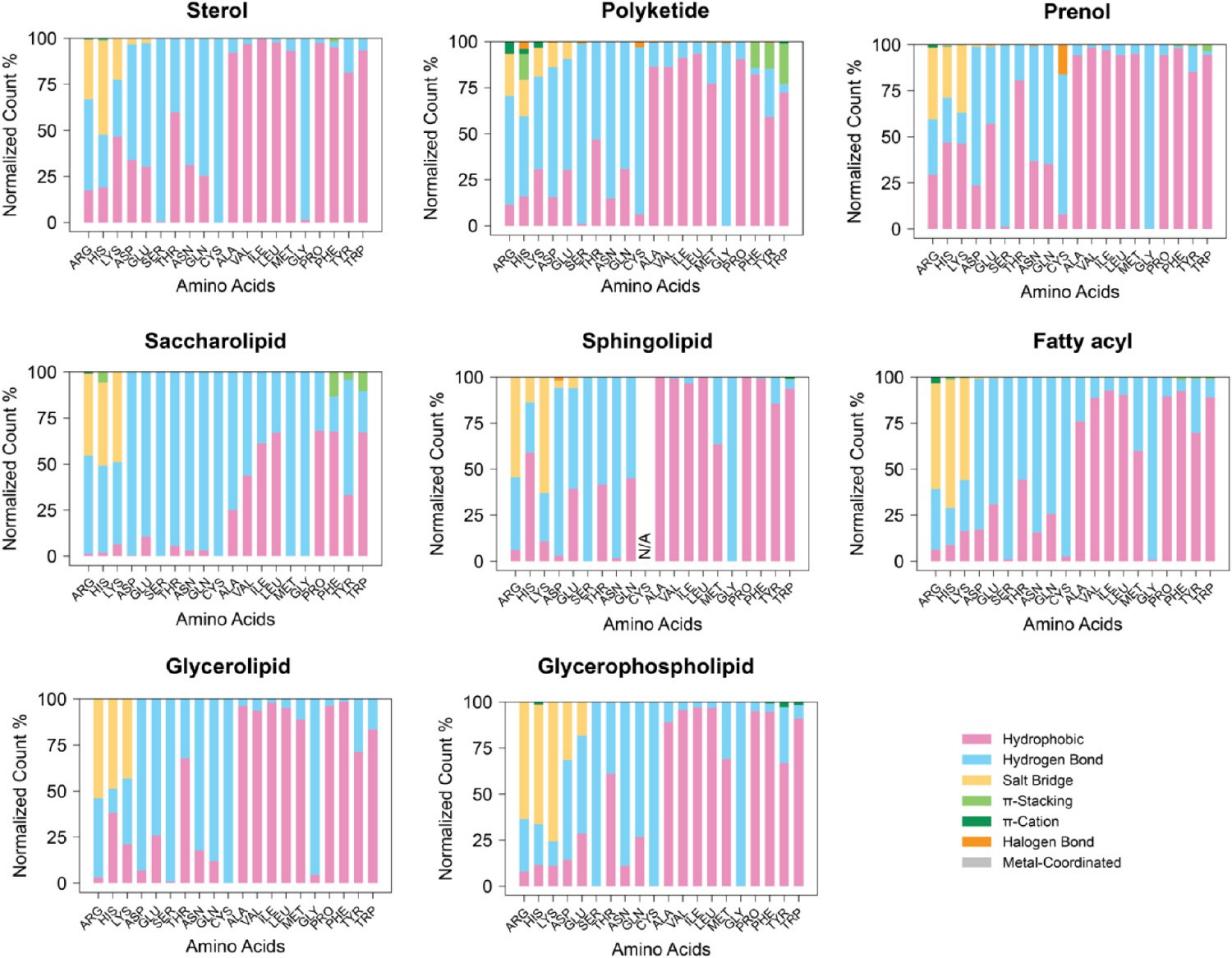
Comparison of amino acid types involved in different types of interactions with lipids across lipid–protein complexes in BioDolphin. Normalized interaction count (normalized for different interaction types within the same amino acid) for the eight classes of lipids. Interaction counts were obtained from a PLIP analysis of all lipid–protein complexes in the data set. N/A: not applicable since no interactions between Cys residues and sphingolipid atoms were detected.

#### Hydrogen Bonds

Hydrogen bonds were the second most common interaction type in lipid–protein complexes with a total of 76,686 contacts (Figure 2). Hydrogen bonding was predominantly mediated by side chains of polar and charged amino acids, such as Arg, Ser, Asp, Gln, Asn, Thr, and Tyr (Figure 4). Other residues, such as Glu, Trp, Leu, and Gly, participated to a lesser extent, often via interactions with backbone amide or carbonyl groups. While the exact ranking of residues varies between lipid classes, this pattern highlights that hydrogen bonding in lipid–protein complexes is largely driven by polar and charged side chains with some contribution from backbone atoms.^75^ We evaluated other characteristics associated with hydrogen bond strength: distance between atoms, angles between atoms, and the types of atoms involved.^75^ The median distance between hydrogen bond donor and acceptor atoms between protein and lipid atoms ranged from ≈3.1 to 3.5 Å (Figure S5). The median distance between hydrogen and acceptor atoms spanned ≈2.4 to 2.7 Å (Figure S6). These values are consistent with hydrogen bond distances observed in small molecule–protein complexes.^76^ Median hydrogen bond distances were slightly increased in glycerolipid–protein and glycerophospholipid–protein complexes, potentially because the interactions occur at hydrated interfaces, involving flexible and highly solvated headgroups. The most common acceptor atom types in hydrogen bonds between lipid and protein atoms were sp2 hybridized oxygens (i.e., carbonyls) and sp3 hybridized oxygens (i.e., hydroxyls) (Figure S7). There were notable lipid class-specific observations: fatty acyls showed an elevated count of carboxylate oxygens as hydrogen bond acceptor atoms, consistent with terminal carboxylate groups present in fatty acyls. The most common donor atom types in hydrogen bonds between lipid and protein atoms were sp2 hybridized nitrogen atoms (i.e., imines) and sp3 hybridized oxygens (i.e., hydroxyls) (Figure S8). The median angle at the hydrogen bond donor atom ranged from ≈135 to 143 ° (Figure S9). A large majority of hydrogen bonds originated from protein side chain atoms (Figure S10). However, backbone contributions also played a major role ranging from 30% of contributions in sterols and sphingolipids to 84% of contributions in fatty acyls. This indicates that amino acid side chains are major drivers of hydrogen bonding in lipid–protein interactions with additional backbone participation depending on the lipid class. Together, these data support that hydrogen bonds contribute significantly to lipid–protein interactions in a lipid class-specific manner through a unique mechanism relative to hydrophobic effects and van der Waals contacts.^77,78^

#### Salt Bridges

Salt bridges were the third most common interaction type in lipid–protein complexes with a total of 28,142 contacts (Figure 2). A clear pattern emerged when examining the amino acid contributors to salt bridges. In lipid classes with the highest salt bridge counts (glycerophospholipids, fatty acyls, and glycerolipids), salt bridges were almost entirely driven by residues with positively charged side chains, such as Arg, His, and Lys (Figure 4). Residues containing negatively charged side chains, such as Asp and Glu, also contributed to salt bridges to a lesser extent. This asymmetry is consistent with observations that most biological lipids are zwitterionic or anionic at physiological pH, favoring electrostatic interactions with cationic protein residues.^1^ There were notable differences between lipid classes. For example, polyketides did not engage in as many salt bridges as other lipid classes (Figure 4), likely due to the general lack of charged headgroups or strongly ionizable moieties. We evaluated the important features for salt bridge characteristics: distance between atoms and the types of atoms involved.^79^ The median salt bridge distance between lipid and protein atoms ranged from ≈4.2 to 4.9 Å (Figure S11). This median is slightly higher than ideal salt bridge geometries that occur in small molecule–protein complexes in the 2.8 Å to 3.9 Å range, indicating overall weaker salt bridges, but still within PLIP’s salt bridge distance maximum of 5.5 Å.^47,79,80^ The lipid chemical groups that formed salt bridges with protein residues included carboxylates, guanidines, phosphates, quaternary amines, sulfates, sulfonic acids, sulfoniums, and tertiary amines (Figure S12). Among these, carboxylates and phosphates were the most frequently represented. Some interactions were quite lipid class-specific, such as sulfonic acids in glycerolipids and tertiary amines in polyketides. Together, this analysis supports that salt bridge formation in lipid protein complexes is primarily driven by lipid headgroup chemistry and charge state, which could modulate interaction strength and fine-tune specificity across lipid classes.^81,82^

#### π-Stacking Interactions

π-stacking interactions were the fourth most common interaction type in lipid–protein complexes with a total of 2,182 contacts (Figure 2). π-stacking interactions were most common in polyketide–protein and saccharolipid–protein complexes where they occurred between amino acids with aromatic side chains, such as Phe, Tyr, Trp, and His, and the aromatic moieties of lipids (Figure 4). We next evaluated important features for π-stacking characteristics: the centroid distance (the distance between the geometric centers of two interacting aromatic rings), the π-stacking orientation, and the π-stacking angle.^83,84^ The centroid distance for π-stacking interactions between lipid atoms and protein atoms ranged from ≈3.8 to 5.3 Å (Figure S13). As a representative example of lipid class-specific differences, π-stacking interactions for sterol–protein complexes showed centroid distances skewed toward ≈5.1 Å, representative of weaker edge-to-face (T-shaped) arrangements (Figure S13 and Figure S14). Saccharolipid–protein and prenol–protein complexes exhibited centroid distances skewed toward ≈3.8 Å, representative of stronger parallel face-to-face arrangements (Figure S13 and Figure S14). Some assemblies, such as polyketide–protein and fatty acyl-protein complexes, showed distributions of both T-shaped and parallel π-stacking interactions (Figure S13 and Figure S14). This likely represents the diversity in the size and geometry of aromatic rings across different lipid classes. The median π-stacking angle ranged from ≈11.7° to 84°, with larger angles (>40°) corresponding to T-shaped arrangements and smaller angles (<40°) corresponding to parallel arrangements (Figure S15). Together, this analysis highlights clear lipid class-specific differences in π-stacking geometries that likely contribute to the selectivity and stability of the lipid–protein complexes.

#### π-Cation Interactions

π-Cation interactions were the fifth most common interaction type in lipid–protein complexes with a total of 790 contacts (Figure 2). π-Cation interactions were most common in polyketide–protein complexes where cations were mediated by positively charged side chains of Arg, Lys, and His, or π contributions from aromatic residues, such as Trp, Tyr, and Phe (Figure 4). We evaluated the features of π-cation interactions: distance, lipid group, and information on which molecule provides the charge.^85^ The median π-cation distance between lipid and protein atoms spanned ≈3.9 to 4.9 Å and showed class-specific differences (Figure S16). The lipid chemical groups that formed π-cation interactions with protein residues included aromatics, guanidines, quaternary amines, and tertiary amines (Figure S17). Among these, aromatics and quaternary amines were the most frequently involved across lipid classes. Some interactions were lipid class-specific, such as guanidines and tertiary amines present in π-cation interactions with polyketide–protein and prenol–protein complexes. For lipids usually lacking substantial charged groups, such as sterols, polyketides, prenols, and saccharolipids, the protein supplied the cation (Figure S18). For lipids that often contain intrinsically charged head groups, including sphingolipids and glycerophospholipids, the lipid provided the cation. The data provide evidence that, although relatively rare, π-cation interactions can contribute to lipid–protein recognition in a lipid class-dependent manner with the presence and origin of charged moieties dictating both interaction strength and geometry.^86,87^

#### Halogen Bonds

Halogen bonds were the sixth most common interaction type in lipid–protein complexes with a total of 121 contacts (Figure 2). Halogen bonds are directional noncovalent interactions involving a halogen atom (Cl, Br, I, or F) donor and an electron-rich acceptor (O, N, or S), similar to hydrogen bonds.^88^ While halogen bonds are rare in naturally occurring lipid–protein complexes due to the low abundance of halogen atoms in biological lipids, they can play important roles in interactions involving both natural and synthetic halogenated.^89^ Halogen bonds were found in prenol–protein and polyketide–protein complexes where Cys contributed most to the interactions due to its thiol side chain, which provides an electron-rich sulfur atom capable of forming favorable interactions with halogenated prenol and polyketide headgroups (Figure 4). We evaluated defining features of halogen bonds: distance, acceptor/donor type, and angle.^88^ The median distance between halogen bond donor and acceptor atoms between protein and lipid atoms across all lipid classes ranged from ≈2.9 to 3.8 Å (Figure S19). This is consistent with the halogen bond distances observed in small molecule–protein complexes.^76^ The most common acceptor atom types involved in lipid–protein halogen bonds were sp2 hybridized oxygens (i.e., carbonyls) and sp3 hybridized oxygens (i.e., hydroxyls) (Figure S120). Prenols displayed elevated numbers of sp3 hybridized sulfurs (i.e., thiols) and positively charged nitrogen, whereas fatty acyls were enriched in aromatic nitrogens and sp3 hybridized nitrogens (i.e., tetrahedral amines). The most common donor atom types in halogen bonds between lipid and protein atoms were bromine, chlorine, and fluorine (Figure S21). The median angle at the halogen bond acceptor atom ranged from ≈104 to 146°, while the donor atom angle ranged from ≈144 to 154° (Figure S22). Apart from sterol–protein and polyketide–protein complexes, the vast majority of halogen bonds originated from interactions with protein side chain atoms rather than the peptide backbone (Figure S23). This indicates that side chains are the primary drivers of halogen bonding in lipid–protein interactions, with some backbone participation depending on the lipid class. The analysis implies that halogen bonding in lipid–protein complexes is rare but does occur halogenated lipid moieties where it likely contributes to fine-tuning the interaction strength, geometry, or specificity.

#### Metal-Coordinated Interactions

Metal-coordinated interactions, in which the metal atom is part of the lipid structure, were the rarest type of interaction with a total of only 26 contacts (Figure 2). In very rare instances, metals may be coordinated by lipids or incorporated directly into the lipid chain. This phenomenon is rarely observed in natural lipids and more commonly a feature of synthetic lipid analogs.^90^ Metal-coordinated interactions were only found in prenol–protein and fatty acyl-protein complexes where they were mediated by Cys, His, and Asp residues (Figure 4). We evaluated important features for metal complex interactions: distances between atoms and the type of metal involved.^91^ The median metal complex distance between lipid and protein atoms across all lipid classes ranged from ≈2.2 to 2.8 Å (Figure S24). The metal types present in lipids that formed metal-coordinated interactions included gold, iron, and ruthenium. In fatty acyl-protein complexes, metals were found in myocrisin (CCD ID MYQ—a gold containing synthetic lipid analog used as an antirheumatic drug^92^), ferrioxamine B (CCD ID 0UE—a natural iron binding lipid that serves as a siderophore^93^), and desferrioxamine B (CCD ID BJ5—a natural iron binding lipid that serves as a siderophore^94^) (Figure S25). In prenol–protein complexes, ruthenium was present in *p*-cymene ruthenium chloride (CCD ID RU7—a synthetic ruthenium containing lipid used as an anticancer agent^95^) (Figure S25). These examples illustrate that even rare metal-coordinated interactions can mediate specialized lipid–protein recognition with functional consequences in both natural lipid biology and drug design.

### Comparison of Lipid–Protein Interactions across Protein Families

We next sought to obtain a big picture view of the similarities and differences between how the eight classes of lipids interact with proteins of unique folds and functions. Toward this, we utilized a tool called ProteinCartography.^49^ In cluster mode, ProteinCartography takes as input a set of PDB structures (i.e., all sterol–protein complexes), performs an all-versus-all structural comparison using Foldseek to calculate template modeling (TM)-scores, clusters the structures using the Leiden algorithm, and visualizes the resulting clusters with a t-distributed stochastic neighbor embedding (t-SNE) multivariate analysis plot.^49,58^ The t-SNE plots allow us to identify trends protein family specific structural landscapes across the eight different classes of lipids. Side-by-side with the ProteinCartography analysis, we evaluated (1) the secondary structure of protein atoms within 5 Å of the bound lipid, and (2) the PLIP-determined interaction profiles across unique protein family (PFAM) annotations.^96^ The results of these analyses for each lipid class are described below.

To discern whether certain lipid classes are dominated by a small number of protein families or folds, we quantified the distribution of the 40 most populated Leiden clusters derived from ProteinCartography, which serve as a proxy for protein family level redundancy, against the total number of PDB entries in the data set. A clear distinction emerged between lipid classes. The sphingolipid and saccharolipid data sets, which contained the fewest lipid–protein structures, were highly redundant: a small number of Leiden clusters dominate the data with fewer than 20 clusters accounting for ∼80–100% of all entries (Figure S26). This suggests that lipid classes for which experimental lipid–protein structures are relatively scarce are effectively represented by only a limited set of protein families or folds. All other lipid classes required substantially more clusters to achieve comparable coverage with at least 40 clusters needed to capture ∼78–92% of entries (Figure S26). This indicates that most lipid–protein complex data sets across different lipid classes are not dominated by a small number of protein families, but instead reflect a broader distribution of protein families and structural folds. Overall, while some lipid classes are strongly enriched in a few well-sampled families, most data sets exhibit substantial cluster diversity, indicating that the observed trends are not typically driven by repeated sampling of a limited set of protein folds or families.

#### Sterol Binding Proteins

Previous reports provide features of some classes of sterol binding proteins. For example, cholesterol tends to associate with proteins at structured interfaces where its C3 hydroxyl group forms hydrogen bonds with polar residues, while its steroid ring engages hydrophobic residues with interaction hotspots localized to C21 and C26 atoms.^97–99^ However, the broader structural landscape of sterol binding proteins remains to be characterized. In our data set, ProteinCartography analysis revealed that sterol binding proteins were represented by 62 Leiden clusters (LC) that included PFAMs spanning G-protein coupled receptors, lipocalins, cytochrome c oxidases, nuclear hormone receptors, ABC transporters, ion transports, TRP channels, cytochrome P450s, immunoglobulin fold containing proteins, lipid transport proteins, and more (Figure 5). Most proteins interacted with sterols primarily through α-helices or a combination of α-helices and loops, highlighted by LC04 (ex: cytochrome c oxidase subunit I), LC49 (ex: TRPM2 chanzyme), and LC33 (ex: V-type proton ATPase 16 kDa proteolipid subunit c) (Figure S27). A smaller subset of proteins engaged sterols via disordered regions and loops, such as LC20 (ex: ferrochelatase) and LC57 (ex: plasmepsin II) (Figure S27). Finally, a few protein folds interacted with sterols via residues on β-sheets or mixed α/β content, including LC28 (ex: IgG1 Kappa antibody), LC37 (ex: σ nonopioid intracellular receptor 1), and LC40 (ex: fatty acyl-binding protein 6) (Figure S27). While most sterol–protein interactions were driven by hydrophobic interactions, some interaction profiles were skewed when comparing sterol–protein interactions across different PFAMs (Figure S28). For example, cytochrome c oxidase subunit VIa (PF02046) exhibited an increased number of salt bridges with charged sterol alkyl side chain moieties via His residues, while estrogen receptor α (PF00105) showed increased π-stacking interactions with the steroid A and B rings via Phe residues. These differences may underlie selectivity of sterol recognition within and across diverse protein families.

**Figure 5:**
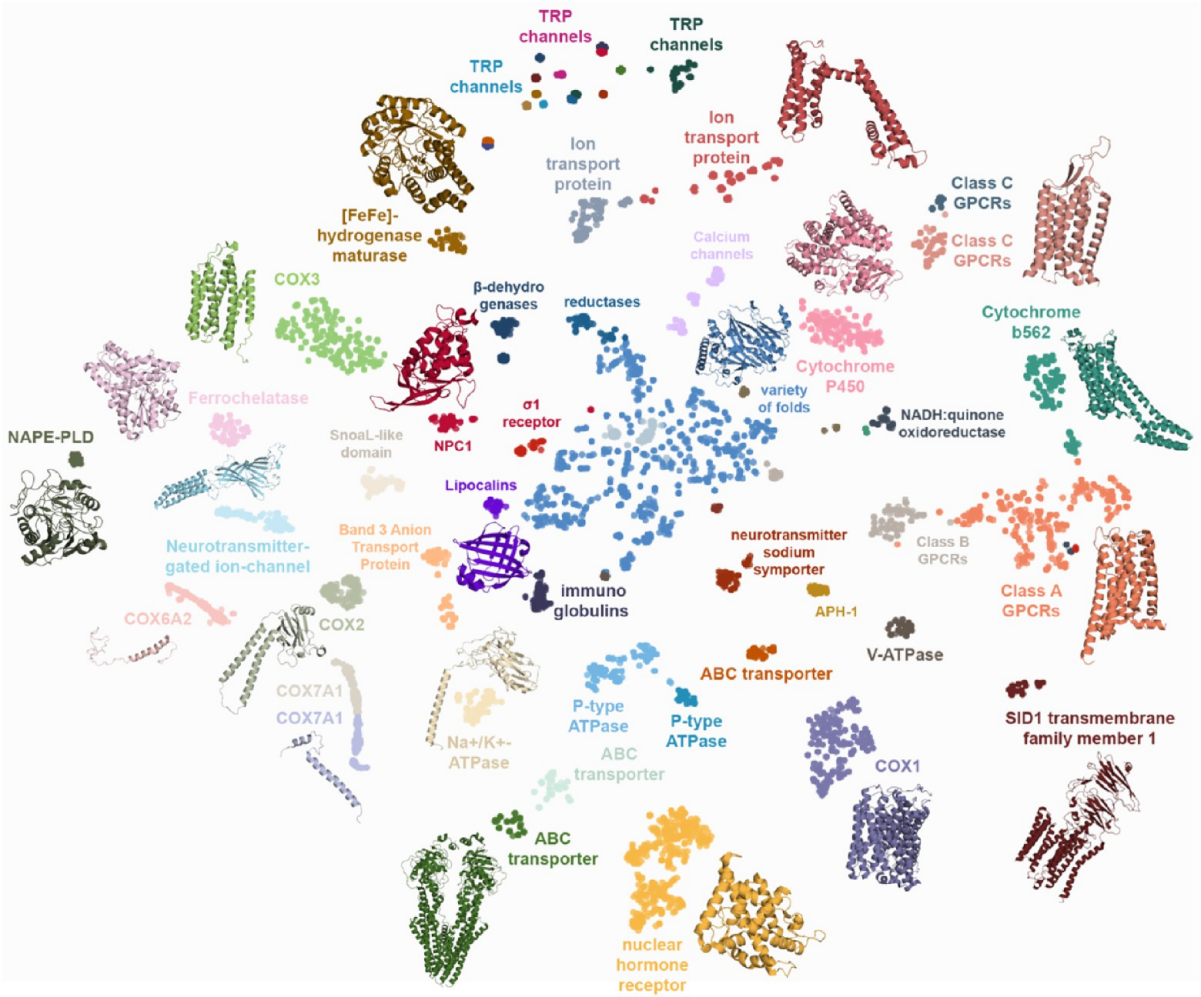
The structural landscape of sterol binding proteins. t-SNE plot of structure-based clustering of sterol binding proteins from BioDolphin using the ProteinCartography tool. The 62 Leiden clusters are colored by cluster assignment. Representative experimentally determined structures are displayed for selected clusters.

#### Polyketide Binding Proteins

A bulk of the information on polyketide binding proteins comes from work on polyketide-associated biosynthetic enzymes and polyketide-based antibiotic inhibitors.^100,101^ The broader structural landscape of polyketide binding proteins remains to be characterized. Polyketide binding proteins were represented by 34 Leiden clusters that included PFAMs spanning nuclear hormone receptors, biosynthetic enzymes (polyketide synthases, polymerases, isomerases, deacetylases, transferases, etc.), ion channels, efflux pumps, cytochrome P450s, ribosomal proteins, transthyretin, and more (Figure 6). Many proteins interacted with polyketides through a combination of α-helices, loops, and β-sheets, such as LC16 (ex: chalcone isomerase), LC24 (ex: serine protease chymase), and LC32 (ex: transcriptional activator LasR) (Figure S29). Another large set of proteins engaged polyketides via α-helices and loops, highlighted by LC05 (ex: V-type proton ATPase subunit c), LC15 (ex: potassium voltage-gated channel subfamily KQT member 2), and LC20 (ex: TRPV2). Finally, a few protein families interacted mainly through β-sheets, such as LC07 (ex: transthyretin). While most polyketide–protein interactions were driven by hydrophobic interactions and hydrogen bonds, some types of interactions were skewed when comparing polyketide–protein interactions across different PFAMs (Figure S30). For example, HIUase/transthyretin (PF00576) showed an elevated frequency of π-cation interactions via polyketide aromatic rings with Arg residues. Zinc finger in Ran binding protein (PF00641) and C3HC4 type zinc finger (PF13920) showed a complex network of interactions including salt bridges, halogen bonds, and π-stacking in addition to hydrophobic interactions coordinated by Ser and His residues. These trends suggest that polyketide-binding proteins employ diverse structural motifs to accommodate chemically versatile ligands and may tune interaction types according to protein family.

**Figure 6:**
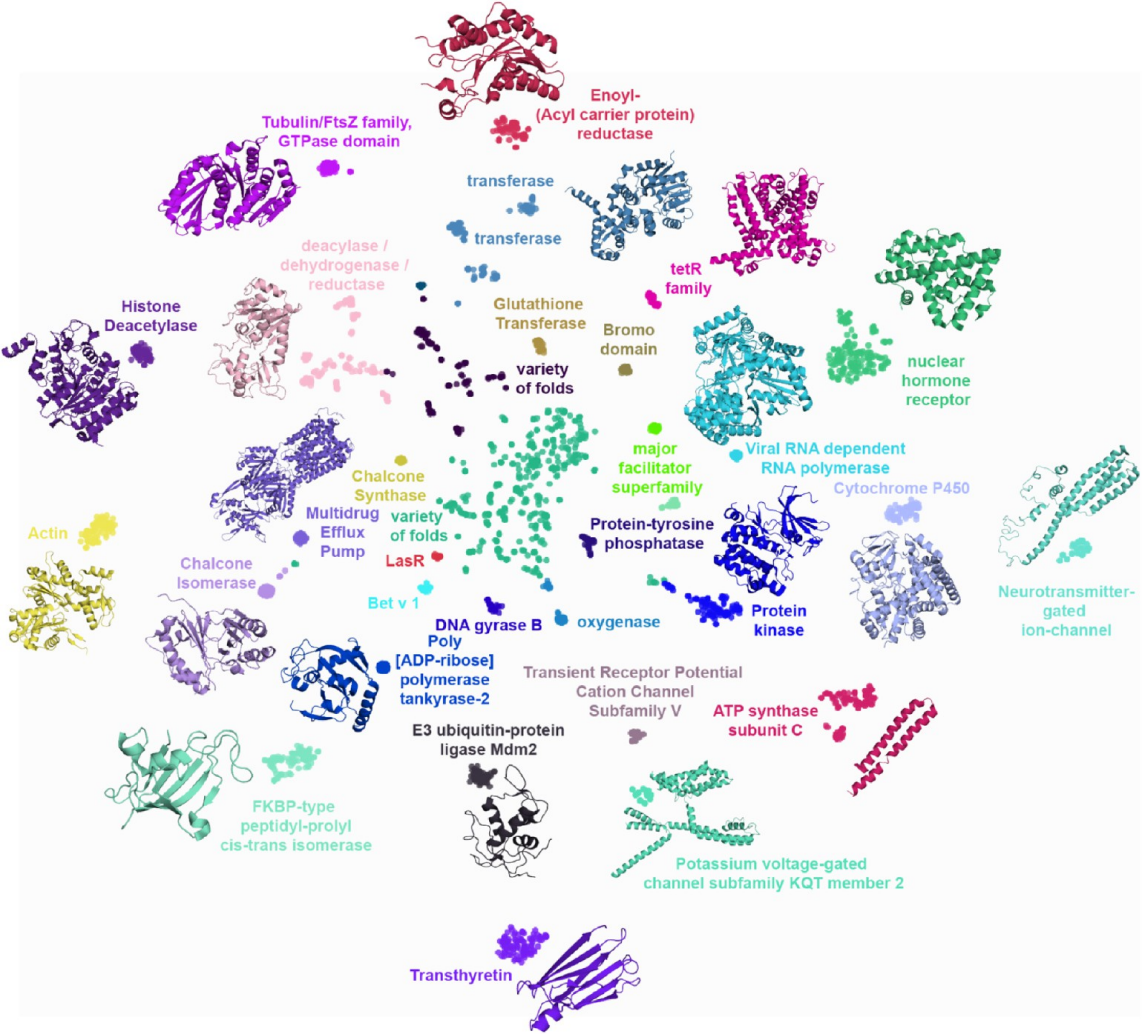
The structural landscape of polyketide binding proteins. t-SNE plot of structure-based clustering of polyketide binding proteins from BioDolphin using the ProteinCartography tool. The 34 Leiden clusters are colored by cluster assignment. Representative experimentally determined structures are displayed for selected clusters.

#### Prenol Binding Proteins

Examples of well-known prenol binding proteins include biosynthetic enzymes involved in isoprenoid synthesis and prenol-associated post-translation modifications (i.e., prenylation).^102,103^ The broader structural landscape of prenol binding proteins remains to be characterized. Prenol binding proteins were represented by 112 Leiden clusters that included PFAMs spanning G-protein coupled receptors, light harvesting assemblies (photosystem I, photosystem II, antenna complex, chlorophyll A-B binding proteins), cytochrome P450s, biosynthetic enzymes (farnesyltransferases, geranylgeranyltransferases, synthases, synthetases, reductases), and more (Figure 7). Most proteins interacted with prenols through a combination of α-helices and loops, highlighted by LC006 (ex: chlorophyll A-B binding protein), LC032 (ex: FPP farnesyltransferase), and LC052 (ex: rhodopsin) (Figure S31). A small percentage of PFAMs interacted with minor contributions from β-sheets, such as LC20 (ex: orange carotenoid-binding protein), LC36 (ex: cellular retinol-binding protein II), and LC43 (ex: β-lactoglobulin) (Figure S31). Most prenol–protein interactions across different PFAMs were driven by hydrophobic interactions with minor contributions from other interaction types (Figure S32). Chlorophyll A-B binding protein (PF00504) exhibited a slightly elevated frequency of hydrogen bonds with prenol hydroxyl groups via Asp side chain and backbone amide atoms, while photosystem I psaA/psaB (PF00223) and photosynthetic reaction center (PF00124) showed a minor occurrence of π-stacking interactions with prenol aromatic rings coordinated by Phe and Trp residues. These results suggest that, although prenols exhibit chemical diversity, the structural and interaction landscape of their binding proteins is relatively constrained. This convergence indicates that proteins accommodate prenol diversity within a limited set of structural strategies.

**Figure 7:**
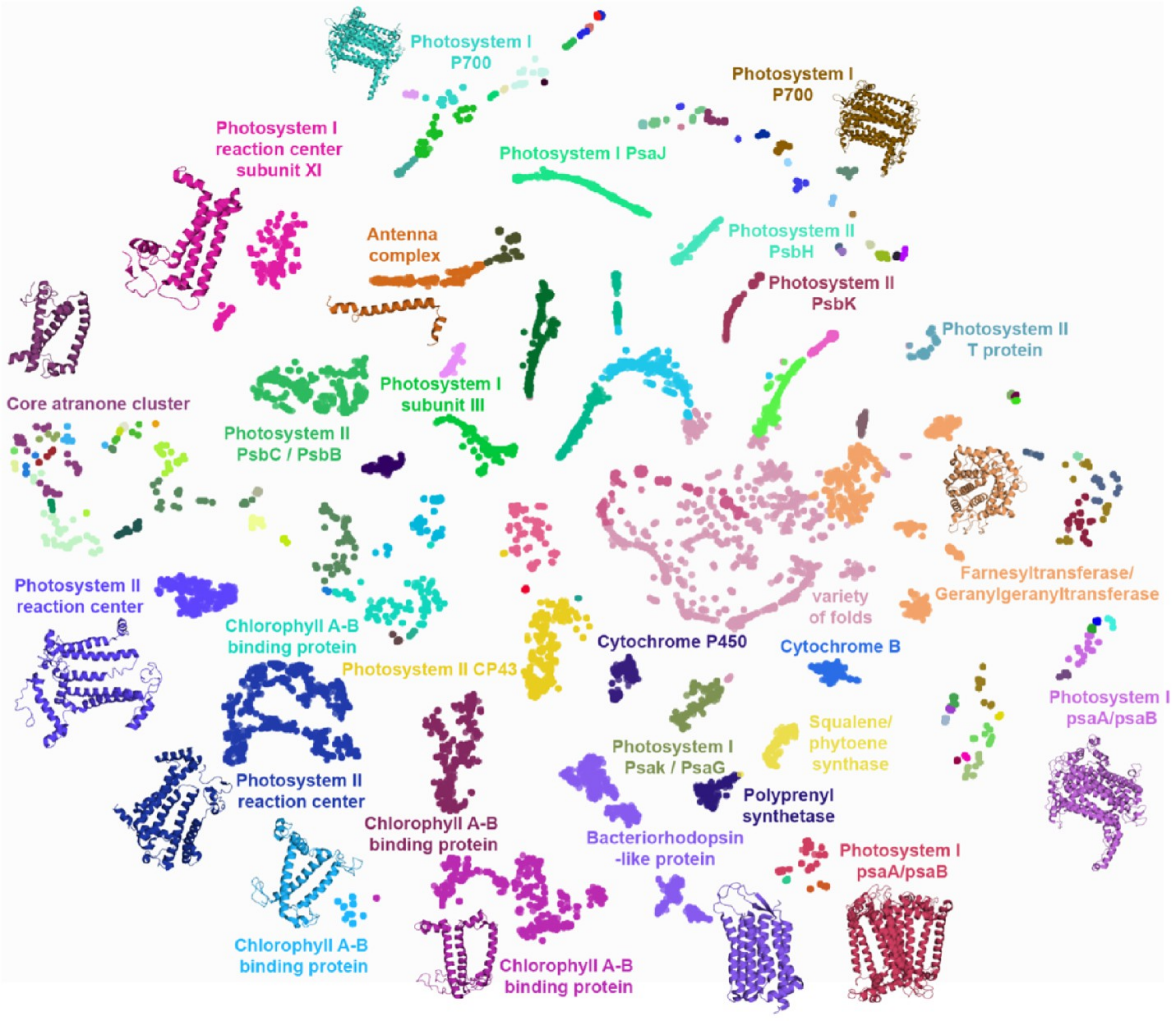
The structural landscape of prenol binding proteins. t-SNE plot of structure-based clustering of prenol binding proteins from BioDolphin using the ProteinCartography tool. The 112 Leiden clusters are colored by cluster assignment. Representative experimentally determined structures are displayed for selected clusters.

#### Saccharolipid Binding Proteins

Saccharolipids are comparatively underexplored, likely due to their restricted phylogenetic distribution and high structural complexity. The most well-studied saccharolipid binding proteins are bacterial transporters, enzymes involved in lipopolysaccharide synthesis and Toll-like receptors that trigger immune responses.^104,105^ However, the broader structural landscape of saccharolipid binding proteins remains to be characterized. Saccharolipid binding proteins were represented by 15 Leiden clusters that included PFAMs spanning a variety of biosynthetic enzymes (transferases, synthases, deacetylases, epimerases, phosphorylases, etc.), flagellar L-ring protein, lymphocyte antigen 96 (forms a complex with Toll-like receptors), and more (Figure 8). Most proteins interacted with saccharolipids through a combination of loops and β-sheets, such as LC06 (ex: UDP-N-acetylglucosamine acyltransferase), LC08 (ex: glycosyl transferase), LC12 (ex: lymphocyte antigen 96), and LC13 (ex: UDP-3-O-[3-hydroxymyristoyl] N-acetylglucosamine deacetylase) (Figure S33). Another set of proteins engaged saccharolipids via α-helices and loops, highlighted by LC00 (ex: UDP-2,3-diacylglucosamine pyrophosphatase) and LC14 (ex: UDP-2,3-diacylglucosamine hydrolase) (Figure S33). Relative to other lipid classes, saccharolipid–protein interactions were highly enriched with hydrogen bonds across different PFAMs (Figure S34). Some notable outliers were identified. For example, calcineurin-like phosphoesterase (PF00149) exhibited an elevated frequency of hydrophobic interactions with saccharolipid hydrocarbon chains via Leu, Val, Ala, and Phe residues. These results suggest that saccharolipid binding proteins are structurally and chemically adapted to complement polar headgroups with hydrogen bonding dominating interactions and pocket geometries relatively constrained, reflecting precise recognition requirements.

**Figure 8:**
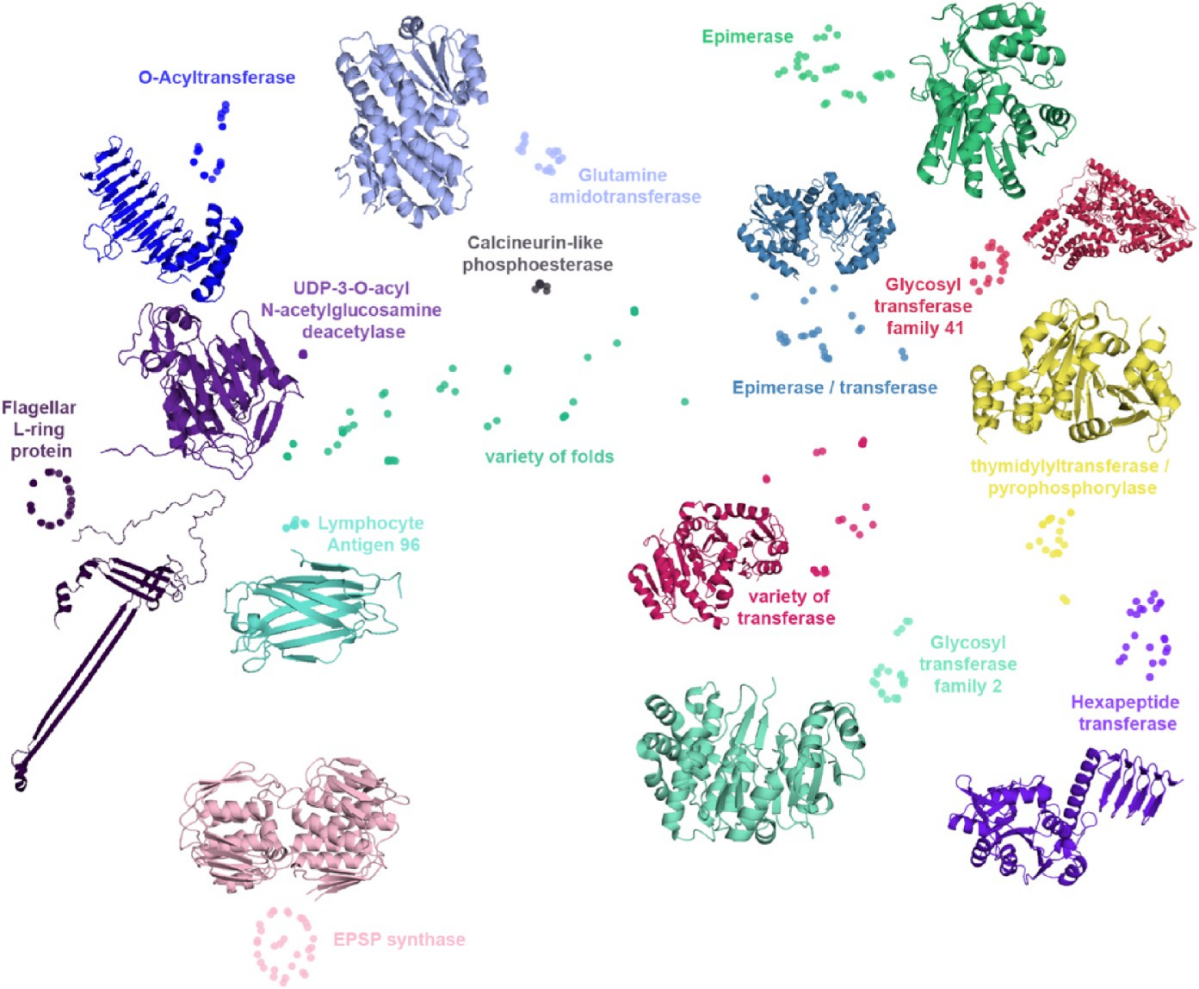
The structural landscape of saccharolipid binding proteins. t-SNE plot of structure-based clustering of saccharolipid binding proteins from BioDolphin using the ProteinCartography tool. The 15 Leiden clusters are colored by cluster assignment. Representative experimentally determined structures are displayed for selected clusters.

#### Sphingolipid Binding Proteins

Information on sphingolipid binding proteins comes from work on receptors, effectors, enzymes, and transporters involved in sphingolipid metabolism, signaling, and membrane structure.^106,107^ The broader structural landscape of sphingolipid binding proteins remains to be characterized. In our data set, sphingolipid binding proteins were represented by 11 Leiden clusters that included PFAMs spanning the immunoreceptors, lipid transfer proteins, transporters/channels, globins, biosynthetic enzymes (ceramidases, transferases, kinases), ORMDL family proteins, and more (Figure 9). Almost all proteins interacted with sphingolipids through a combination of α-helices and/or loops, such as LC03 (ex: ceramide-1-phosphate transfer protein), LC06 (ex: sphingosine 1-phosphate receptor 1), and LC09 (ex: ORM1) (Figure S35). A minor population of proteins engaged sphingolipids also with contributions from β-sheets, highlighted by LC00 (ex: immunoreceptor CD1a), LC02 (ex: ceramide transfer protein), and LC05 (ex: sphingosine kinase 1) (Figure S35). Most sphingolipid–protein interactions across different PFAMs were driven by hydrophobic interactions; however, some interactions were skewed when comparing sphingolipid–protein interactions across different PFAMs (Figure S36). For example, globin family proteins (PF00042) showed an increased content of salt bridges with the negatively charged sphingolipid headgroup with positively charged Arg side chain. Thus, sphingolipid-binding proteins predominantly engage ligands through α-helices and loops via hydrophobic contacts, with selective salt bridges and polar interactions enabling charged headgroup recognition, allowing structurally constrained proteins to accommodate chemically diverse sphingolipid headgroups and hydrophobic tails.

**Figure 9:**
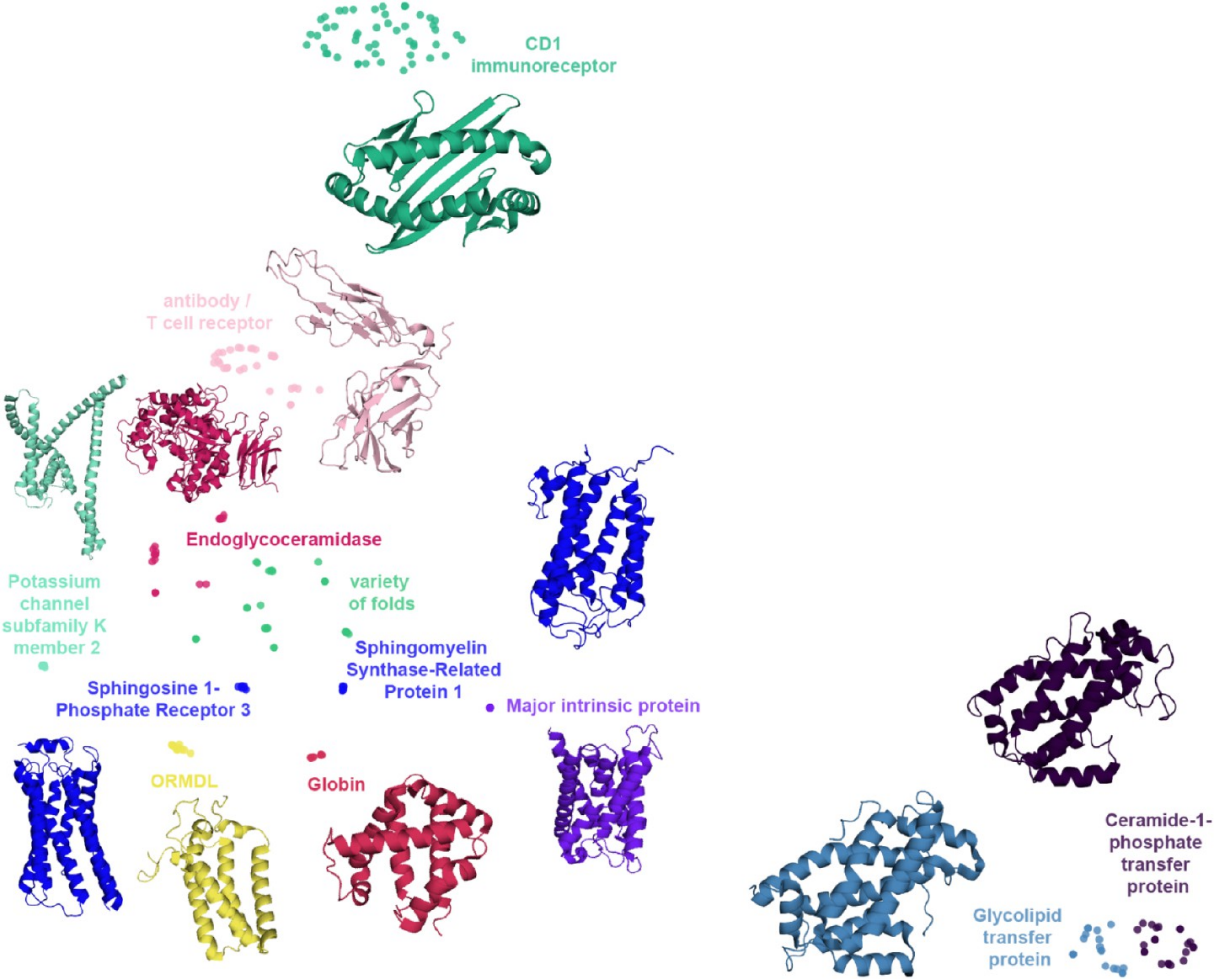
The structural landscape of sphingolipid binding proteins. t-SNE plot of structure-based clustering of sphingolipid binding proteins from BioDolphin using the ProteinCartography tool. The 11 Leiden clusters are colored by cluster assignment. Representative experimentally determined structures are displayed for selected clusters.

#### Fatty Acyl Binding Proteins

Previous literature identified a wide range of fatty acyl binding proteins, such as fatty acyl-binding transport proteins, signaling receptors, and membrane proteins.^108–110^ However, the broader structural landscape of fatty acyl binding proteins remains to be characterized. In our data set, fatty acyl binding proteins were represented by 102 Leiden clusters that included PFAMs spanning G-protein coupled receptors, connexins, albumins, ion channels, enzymes (oxygenases, transferases, hydrolases, kinases, etc.), nuclear hormone receptors, fatty acyl binding proteins, cytochrome P450s, and more (Figure 10). Many proteins interacted with fatty acyls through a combination of α-helices and loops, such as LC020 (ex: two pore calcium channel protein 1), LC031 (ex: cytochrome P450 152A1), and LC060 (ex: serum albumin) (Figure S37). An intermediate amount of protein families interacted also with β-sheets, such as LC000 (ex: fatty acyl translocase platelet glycoprotein 4), LC027 (ex: vitamin B12 receptor), and LC041 (ex: muscle fatty acyl binding protein) (Figure S37). Most fatty acyl-protein interactions across different PFAMs were driven by hydrophobic interactions; however, some types of interactions were skewed when comparing fatty acyl-protein interactions across different PFAMs (Figure S38). For example, neurotransmitter-gated ion channels (PF02931/PF02932) and serum albumin (PF00273) showed an elevated frequency of hydrogen bonds and salt bridges with fatty acyl carboxylate groups via positively charged Arg and Lys side chains. These results indicate that fatty acyl-binding proteins exploit a combination of α-helices, loops, and hydrophobic pockets to engage ligands, with selective salt bridges and hydrogen bonds providing headgroup specificity.

**Figure 10:**
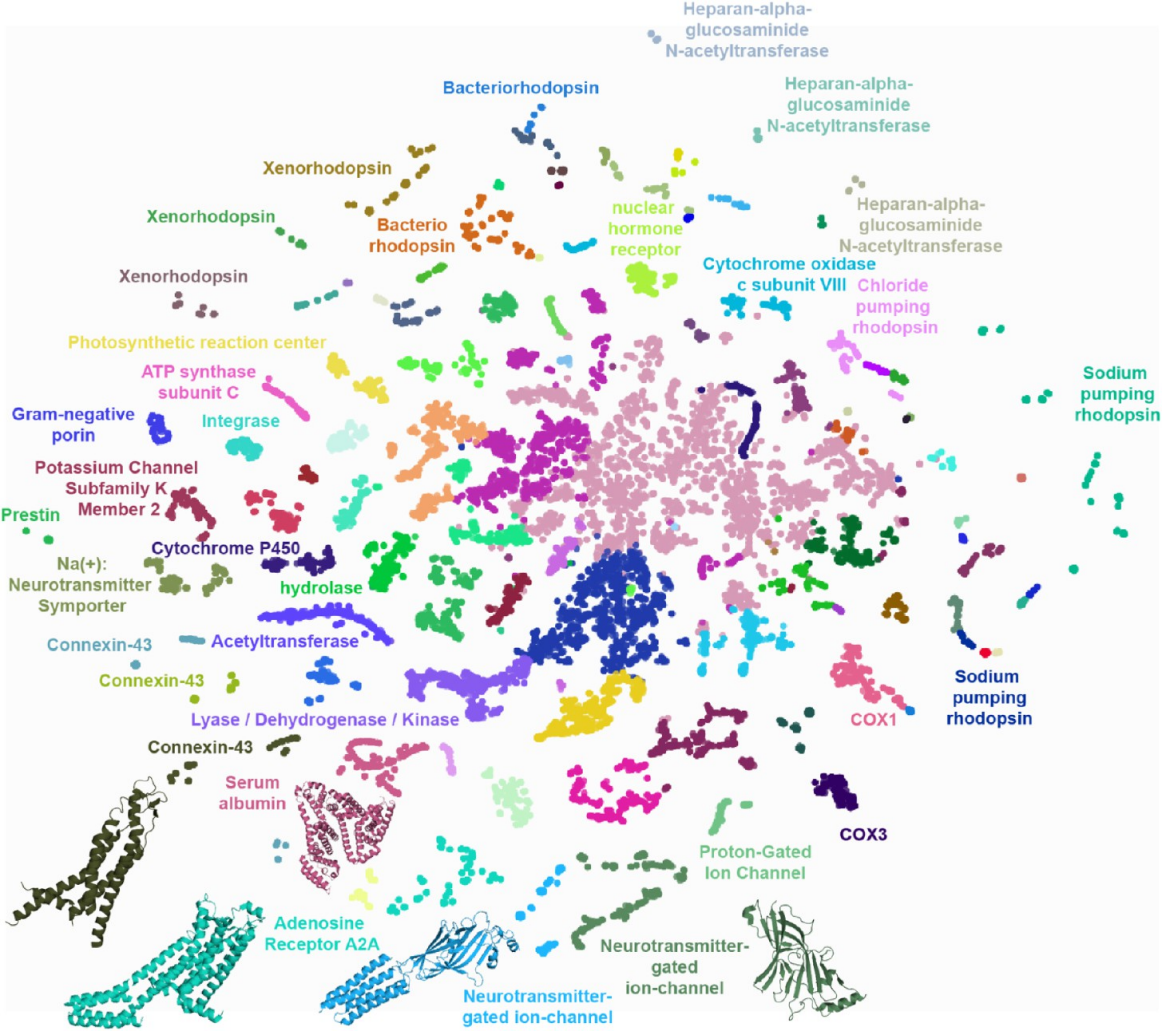
The structural landscape of fatty acyl binding proteins. t-SNE plot of structure-based clustering of fatty acyl binding proteins from BioDolphin using the ProteinCartography tool. The 102 Leiden clusters are colored by cluster assignment. Representative experimentally determined structures are displayed for selected clusters.

#### Glycerolipid Binding Proteins

Information on glycerolipid binding proteins comes from work on proteins involved in glycerolipid synthesis and storage, including their role in energetics and metabolism.^111,112^ The broader structural landscape of glycerolipid binding proteins remains to be characterized. In our data set, glycerolipid binding proteins were represented by 68 Leiden clusters that included PFAMs spanning G-protein coupled receptors, light harvesting assemblies (photosystem I, photosystem II), biosynthetic enzymes (transferases, permeases, flippases, oxygenases, peptidases), transporters, and more (Figure 11). Many proteins interacted with glycerolipids through a combination of α-helices and loops, such as LC07 (ex: COX2), LC30 (ex: tryptophan-rich sensory protein), and LC57 (ex: pannexin-1 channel) (Figure S35). A few protein families interacted also with β-sheets, such as LC35/LC45 (ex: outer membrane protein F), LC49 (ex: σ nonopioid intracellular receptor 1), and LC61 (ex: intimin) (Figure S39). Most glycerolipid–protein interactions across different PFAMs were driven by hydrophobic interactions; however, some types of interactions were skewed when comparing glycerolipid–protein interactions across different PFAMs (Figure S40). For example, photosystem II proteins (PF00421) exhibited an increased presence of hydrogen bonds via glycolipid hydroxyl groups via Asn and Tyr side chains. PsbL (PF02419) showed a slightly elevated occurrence of hydrogen bond and salt bridge interactions with glycolipid hydroxyl groups coordinated by Trp and Arg side chains. These results suggest that glycerolipid-binding proteins employ a diverse set of structural elements and interaction types to accommodate glycerolipid ligands.

**Figure 11:**
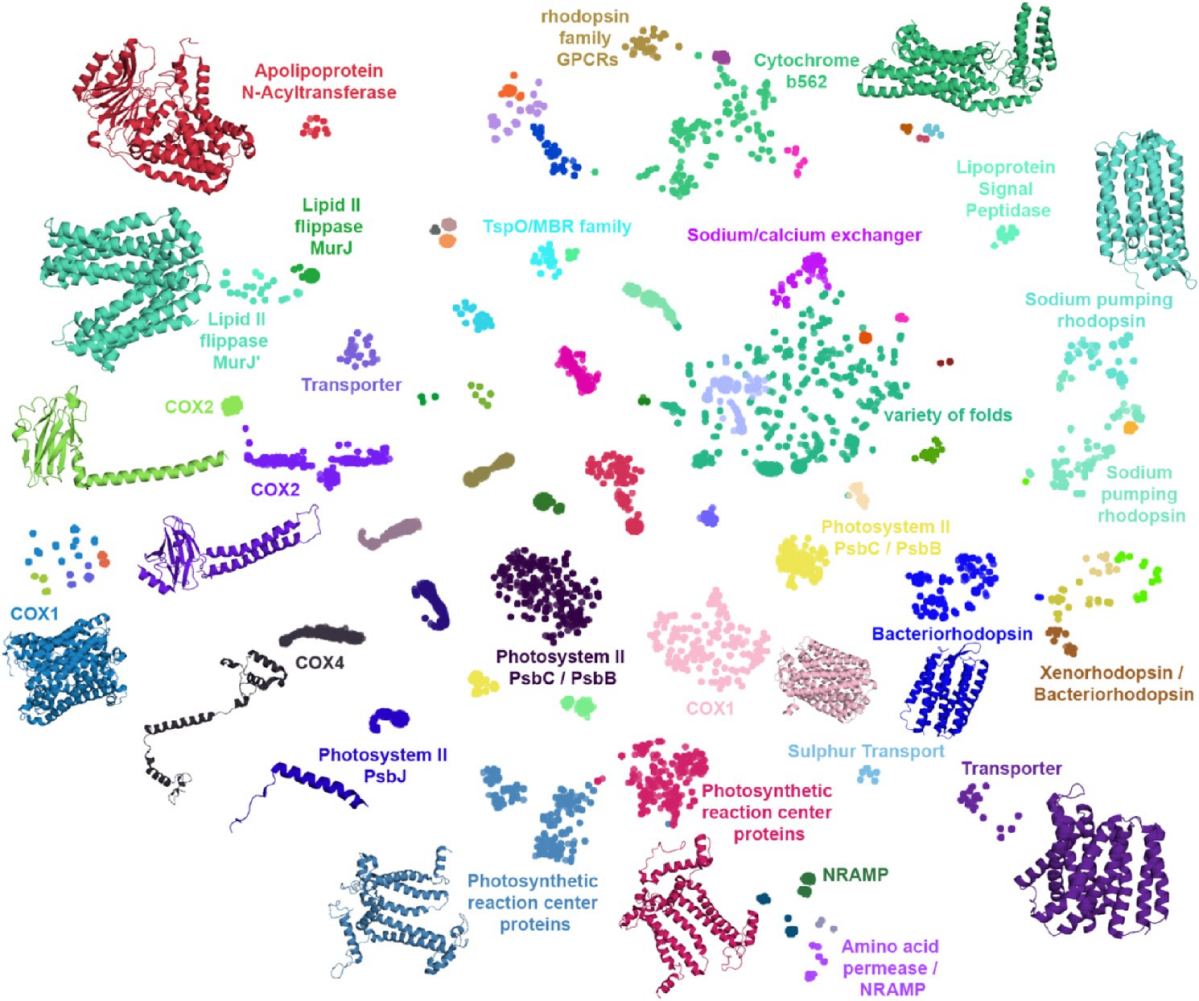
The structural landscape of glycerolipid binding proteins. t-SNE plot of structure-based clustering of glycerolipid binding proteins from BioDolphin using the ProteinCartography tool. The 68 Leiden clusters are colored by cluster assignment. Representative experimentally determined structures are displayed for selected clusters.

#### Glycerophospholipid Binding Proteins

Previous literature has highlighted integral membrane proteins, peripheral membrane proteins, lipid transfer proteins, and signaling receptors as glycerophospholipid binding proteins.^74,113–116^ However, the broader structural landscape of glycerophospholipid binding proteins remains to be characterized. In our data set, glycerophospholipid binding proteins were represented by 113 Leiden clusters that included PFAMs spanning transporters, efflux pumps, connexins, lipid transfer proteins, various receptors, enzymes (dehydrogenases, oxidoreductases, oxygenases), TRP channels, gated ion channels, and more (Figure 12). Many proteins interacted with glycerophospholipids through a combination of α-helices and loops, including LC002 (ex: lipid scramblase TMEM16), LC030 (ex: nicotinic acetylcholine receptor), and LC050 (ex: TRPV5) (Figure S41). A few protein families also interacted with β-sheets, such as LC000 (ex: GM2-activator protein), LC006 (ex: NADH ubiquinone oxidoreductase), and LC011 (ex: CD1b immunoreceptor) (Figure S41). Some types of interactions were skewed when comparing glycerophospholipid–protein interactions across different PFAMs (Figure S42). For example, photosynthetic reaction center protein (PF00124) and photosystem II protein (PF00421) showed an elevated occurrence of hydrogen bonds with charged glycerophospholipid headgroups via Arg and Ser side chains. These results suggest that glycerophospholipid-binding proteins leverage consistent structural motifs while selectively using hydrogen bonds and salt bridges to engage charged headgroups. This combination allows proteins to accommodate the chemical diversity of glycerophospholipids while maintaining generalizable binding strategies important for membrane structure, signaling, and lipid transport.

**Figure 12:**
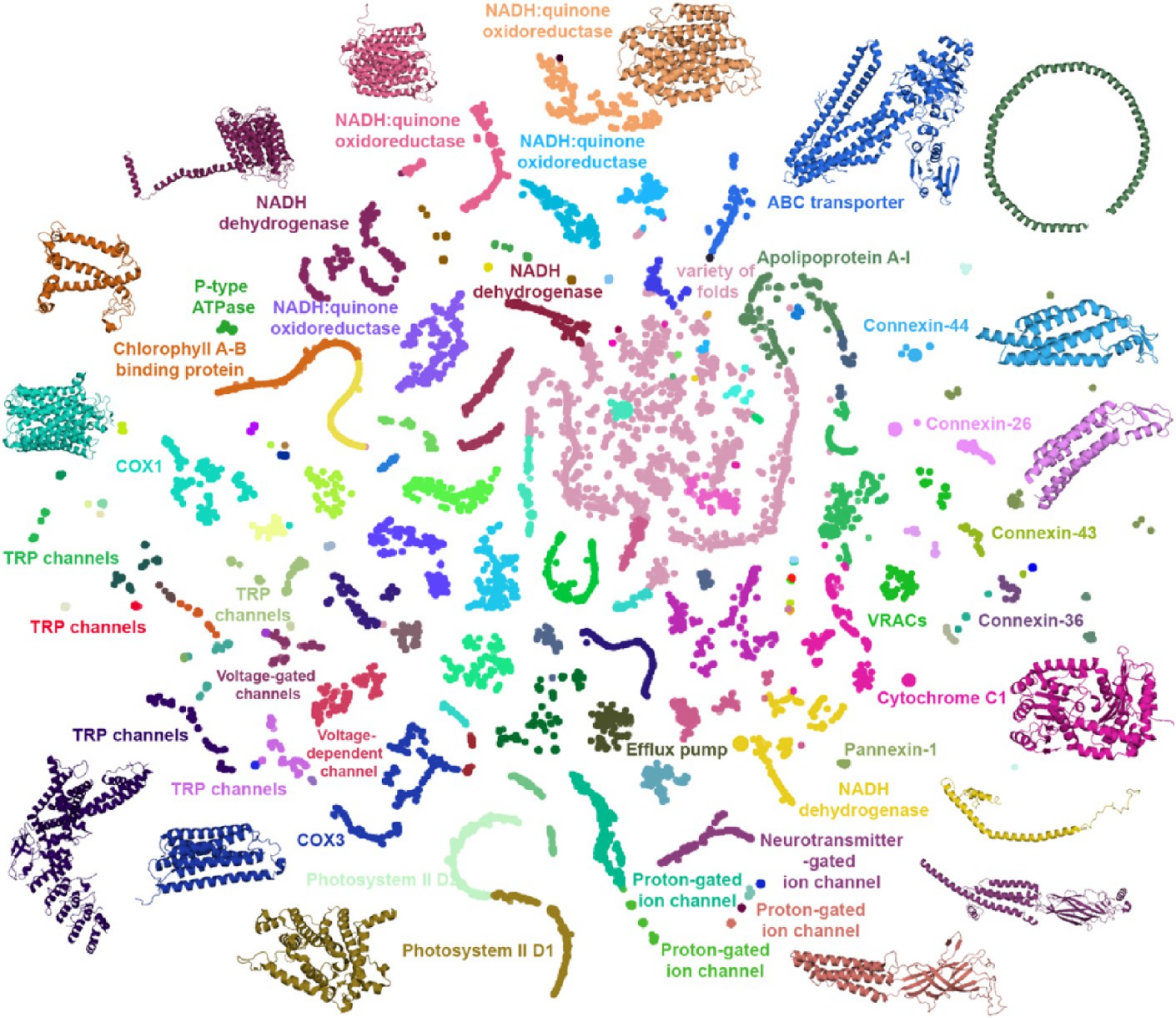
The structural landscape of glycerophospholipid binding proteins. t-SNE plot of structure-based clustering of glycerophospholipid binding proteins from BioDolphin using the ProteinCartography tool. The 113 Leiden clusters are colored by cluster assignment. Representative experimentally determined structures are displayed for selected clusters.

### Similarities and Differences in Interactions of Different Proteins with the Same Lipids

We next evaluated the similarities and differences for how proteins of distinct folds and functions engage with the same lipid species. We generated lipid–protein interaction “heat maps”. PLIP-identified lipid–protein contacts spanning multiple complexes (i.e., all proteins that bind to cholesterol) were aggregated, interaction counts per atom normalized to percentage relative to the highest count, and projected onto the lipid’s three-dimensional structure. This approach enabled direct visualization of conserved versus variable interaction hotspots across structurally diverse protein partners (Figure 13). The interaction heat maps revealed broadly consistent patterns of both polarity-driven and hydrophobicity-driven lipid–protein contacts that vary significantly between lipid classes.

**Figure 13:**
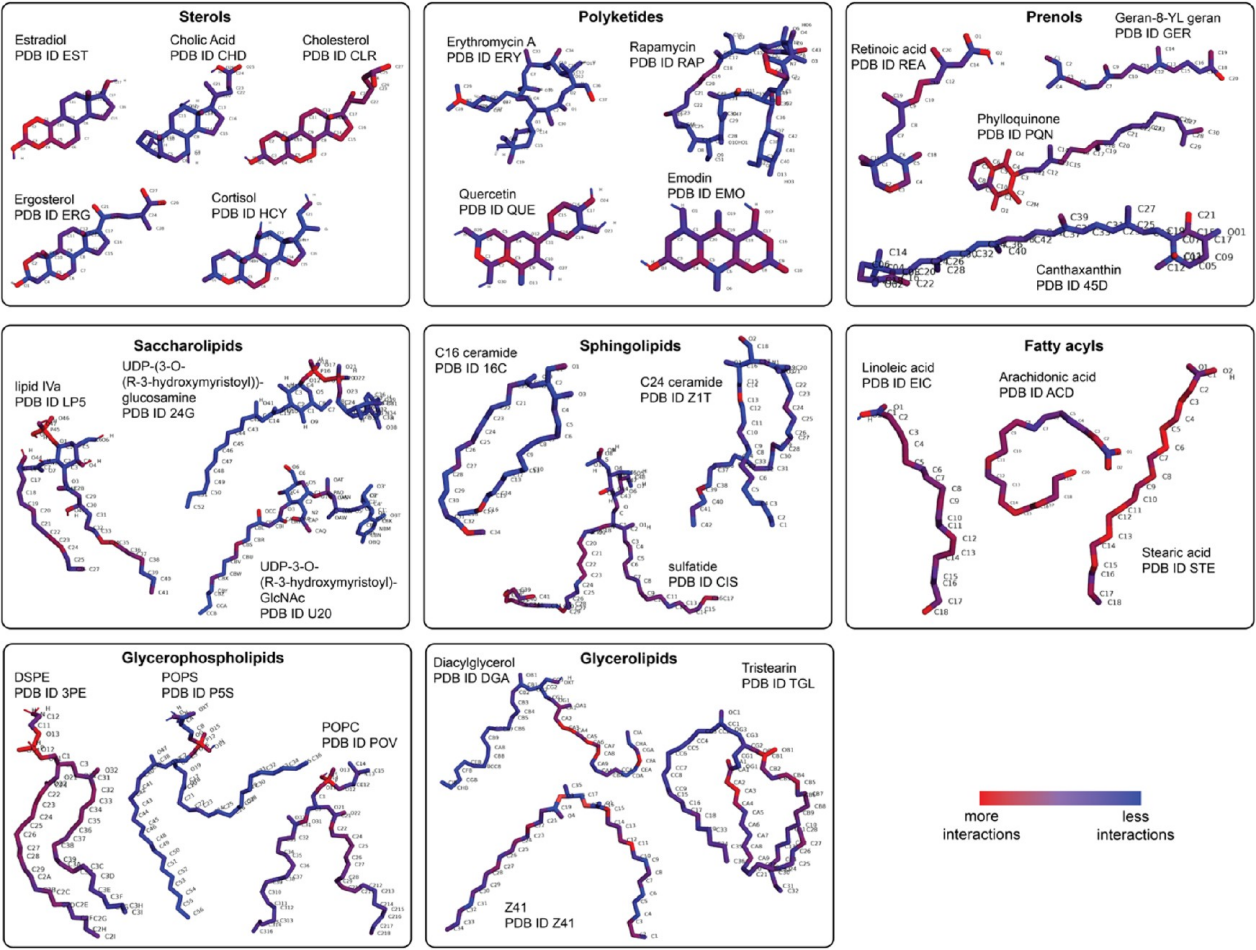
Interaction heat maps for the different classes of lipids. PLIP-identified lipid–protein contacts spanning multiple complexes (i.e., all proteins that bind to cholesterol) were aggregated, interaction counts per atom normalized to percentage relative to the highest count, and projected onto the lipid’s three-dimensional structure. The PDB CCD ID and name of each lipid is provided. The 3D structure of each lipid is colored according to interaction frequency with atoms that interact most frequently in red and atoms that interact least in blue. Atoms are labeled based on their respective PDB CCD ID nomenclature.

In sterols, most interactions cluster to atoms on the A and B steroid rings, which frequently participated in hydrophobic contacts with protein atoms (Figure 13 and Figure S43). The hydroxyl group on the A ring was also a consistent “hotspots”, forming hydrogen bonds that anchored the lipid into protein cavities. In contrast, the C and D steroid rings were “coldspots” forming fewer contacts. Sterol side chain groups often participated in interactions and typically formed hydrophobic or hydrophilic contacts that likely contribute to binding specificity. Polyketides displayed a more distributed interaction pattern where specific hydroxyl groups played key roles (Figure 13 and Figure S44). Polar functional groups, such as hydroxyl and carbonyls, that lined polyketide scaffolds were frequent interaction hotspots, whereas extended hydrophobic scaffolds engaged less commonly and more uniformly. Prenols typically exhibited interaction hotspots concentrated at terminal functional groups (i.e., carboxyls or quinones) with long isoprenoid chains contributing hydrophobic contacts uniformly (Figure 13 and Figure S45). In saccharolipids, glycerolipids, and glycerophospholipids, strong interaction enrichment was present at polar or charged phosphate, carbohydrate, and hydroxyl head groups, which served as dominant anchoring points, while acyl chains exhibited more diffuse contact frequencies (Figure 13 and Figure S46–S50). Sphingolipids showed strong interaction enrichment at the polar or charged head groups (i.e., amide, hydroxyl, and sulfate moieties) and adjacent carbons (Figure 13 and Figure S47). In addition, carbons at the terminal ends of the acyl chains frequently participated in contacts, suggesting that distal tail interactions contribute to binding specificity rather than serving purely nonspecific binding roles. Fatty acyls exhibited strong interaction enrichment at the carboxylate headgroup, consistent with polar anchoring (Figure 13 and Figure S48). In addition, the aliphatic chain typically showed extensive and broadly distributed hydrophobic contacts along their length, much more uniformly than observed for other lipid classes, highlighting a pronounced reliance on continuous chain burial within protein pockets. Overall, lipid–protein recognition profiles across different lipid classes exhibit clear trends, and appear to be driven by a combination of polar headgroup anchoring combined with adaptable, nonspecific tail accommodation within hydrophobic environments.

## Discussion

Lipid–protein interactions are rapidly emerging as key players in most biochemical pathways, and there is a growing interest in harnessing their intrinsic properties for biotechnology and medicine.^4,12,116^ Although the Protein Data Bank contains over ten thousand atomic structures containing bound lipids, lipid–protein complexes have primarily been studied in isolation, such that overarching principles for how proteins recognize chemically distinct lipid classes remain incompletely understood. Lipids are a uniquely diverse group of biomolecules that exhibit an impressively broad range of chemistries, sizes, and biological functions (Figure 1).^20–22^ They interact with proteins using a plethora of mechanisms. Some are bound and transported by soluble proteins through nonannular interactions within partially buried binding pockets, while others participate in annular nonspecific interactions with membrane-embedded proteins.^4,15^ These properties make it difficult to decipher nature’s rules for lipid recognition. Here, we leverage BioDolphin toward a systematic, atomic-level approach to the analysis of 113,782 lipid–protein pairs spanning the eight major lipid classes.^45^ By combining interaction fingerprints with quantification of binding pocket chemistry and geometry, we establish nature’s rules of lipid recognition, revealing conserved and lipid class-specific interaction hotspots among structurally diverse proteins.

Our results highlight several key findings with implications in lipid biochemistry. First, lipid–protein recognition is often, but not always, fine-tuned: protein binding pockets are carefully optimized for lipid size, shape, polarity, and chemistry to allow specific interaction patterns among different classes of lipids. Second, while hydrophobic interactions, hydrogen bonds, and salt bridges are the most abundant, their proportions are lipid class-dependent, which reflects both the structural diversity of lipids and the chemical complementarity of protein binding pockets (Figures 2, 3 and Figure 4). Third, conserved and divergent interaction hotspots on lipid molecules suggest that polar head groups are typically responsible for anchoring specificity, whereas hydrophobic tails are responsible for fine-tuning interactions across a variety of cavities with varying surface areas (Figure 13). Finally, protein family specific patterns support that different lipid chemistries can be accommodated by different protein structural elements using a range of strategies, which emphasizes the modularity of nature’s roles for lipid recognition and functional specialization (Figures 5–12). Apart from informing fundamental lipid biology, the trends identified here have the potential to inform a basis to harness and manipulate lipid–protein interactions. This could include *de novo* design of new-to-nature soluble or membrane-embedded lipid binding proteins,^117,118^ inhibiting lipid–protein interactions by targeting conserved properties of disease-associated lipid–protein complexes,^119^ embedding or anchoring proteins into specialized lipid nanoparticles,^12^ and the design of synthetic lipid analogs that bind to target proteins with enhanced affinity and specificity.^120^

Compared to other biomolecular interfaces, lipid–protein interactions occupy a distinctive physicochemical niche, combining polar headgroup anchoring with adaptable hydrophobic tail engagement to enable both specificity and versatility. Previous reports reveal that small molecule–protein interactions in the PDB are dominated by hydrophobic, hydrogen bond, and salt bridge contacts.^121^ Likewise, protein–protein interfaces are often stabilized by hydrophobic contacts (hydrophobic effect and van der Waals contacts), hydrogen bonds, and salt bridges.^122–124^ These patterns mirror the typical interaction profiles observed across many lipid classes, including sterols, prenols, sphingolipids, and fatty acyls (Figure 2). In nucleic acid–protein complexes, hydrogen bonds tend to dominate with van der Waals and electrostatic interactions tuning interfaces, reflecting the charged nature of DNA/RNA backbones and the balanced aromatic/polar properties of nucleobases.^125–127^ Global analyses of carbohydrate–protein interactions has shown that the major driving force for binding is the hydrogen bond, which is a result of the high density of hydroxyl groups on carbohydrate molecules.^128^ These specific interactions are often accompanied by CH-π and van der Waals interactions between the carbohydrate carbon skeleton.^129^ Carbohydrate–protein interaction profiles are highly consistent with saccharolipid–protein interaction profiles, which similarly exhibit extensive hydrogen bonding due to their carbohydrate head groups. Binding pockets and interface areas differ among biomolecular complexes: small molecules often bind to small, well-defined but promiscuous pockets,^130^ nucleic acids usually interact with large, charged but shallow protein surfaces,^131^ carbohydrates typically engage moderately sized, polar protein pockets,^128^ and protein–protein interfaces involve large, mostly hydrophobic surfaces.^122–124^ Our results indicate that lipid–protein interfaces are likely one of the most variable biomolecular interactions landscapes in nature in terms of their interaction patterns and the geometry of complementary protein pockets. This reflects the massive variability in lipid chemistry and topology. These comparisons imply a high degree of sensitivity to biomolecular class and biochemical function.

There are several notable limitations to this study. First, our data set is limited to experimentally solved lipid–protein structures where lipids are resolved. Many known lipid-binding proteins have been solved either without bound lipids, or with bound lipids where atomic models could not be built due to lipid flexibility or heterogeneity.^132^ Another limitation is that 39.4% of entries in the data set contain lipids for which a subset of atoms could not be modeled due to limited resolution and/or lipid heterogeneity, and 241 CCD IDs (out of 2605 total) did not contain a structure with a fully modeled lipid. Consequently, the resulting interaction profiles may be biased by unresolved lipid regions in PDB structures, particularly for lipids with relatively few available experimental structures or those that exhibit substantial conformational heterogeneity. In addition, some lipid classes, such as saccharolipids and sphingolipids (Figures 8 and 9), were less represented in the data set, which could reflect either limitations in crystallization or a lower natural abundance in cells. In addition, while this work does not directly address how proteins associate with membranes or other lipid-scaffolded structures, the principles of lipid recognition identified here are likely informative for understanding protein–membrane interactions. Second, our study presents a relatively simple cheminformatics approach to defining the lipid–protein interactome. We did not evaluate the energetics of the complexes nor quantify the contributions of different interaction types to the total binding energy. These important aspects should be addressed in future work, for example through deconvolution of interaction energies using the Rosetta force field.^133^ Third, the analyses were performed on static structures (often from X-ray and cryo-EM models) and do not capture the inherent flexibility of both lipid and protein atoms reported in the literature.^134–136^ Large-scale studies of lipid–protein dynamics would be invaluable to the field, and could be achieved through molecular dynamics simulations, predicting conformational ensembles of lipid–protein complexes with AI-based tools, or through high-throughput experimental measurements of conformational exchange.^137–139^ Finally, certain molecular interaction types, such as water bridges and weak hydrogen bonds (for example, X–H···A where X can be C) were not represented in our PLIP or dpocket analysis, despite their biological relevance to lipid–protein recognition.^140,141^

## Conclusions

Overall, this work provides a comprehensive, systems-level view of the lipid–protein interactome, revealing how proteins recognize eight chemically distinct lipid classes through physicochemical principles and context-dependent structural adaptations. Across thousands of lipid–protein complexes spanning all major lipid classes, we show that lipid recognition is governed by finely tuned complementarity between lipid chemistry and protein pocket geometry, where polar head groups often mediate anchoring and specificity, while hydrophobic tails contribute adaptable interactions that stabilize binding across diverse structural environments. At the same time, the observed protein family- and lipid class-dependent variations highlight the modularity and functional specialization that underlie nature’s strategies for lipid recognition. Together, these findings establish a molecular framework for understanding how lipid binding, selectivity, and complex stability are encoded at atomic resolution. Beyond advancing fundamental lipid biology, the principles identified here provide a foundation for the rational design of synthetic lipids, engineered lipid-binding proteins, and chemical probes, offering new opportunities to understand, predict, and manipulate lipid-mediated biology for biotechnology and therapeutic applications.

## Supplementary Material



## Data Availability

In-house scripts to reproduce the results, together with source data from the PLIP, dpocket, and ProteinCartography analyses, are provided via GitHub at https://github.com/mcshanlab/Puri-et-al_Dpocket_PLIP_ProteinCartography_Lipid-Protein_Analysis.

## Notes

Supplementary figures detailing lipid–protein interaction classifications; structural and statistical characterization across lipid classes; protein family and structural clustering analyses; representative examples from the BioDolphin data set (DOCX)
