# Supplementary material for: A Systematic Analysis of Lipid–Protein Interactions in the Protein Data Bank
Source: Biochemistry. 2026 Jun 18;65(13):2123–46. doi: 10.1021/acs.biochem.6c00170 (PMC13348031; doi:10.1021/acs.biochem.6c00170)
Supplement: Supplementary file 1 [file bi6c00170_si_001.docx]

**Supporting Information**

**A systematic analysis of lipid-protein interactions in the Protein Data Bank**Nandita Puri^1^, Andrew C. McShan^1*^

^1^School of Chemistry and Biochemistry, Georgia Institute of Technology, Atlanta, GA 30332,

USA

*Correspondence: Andrew C. McShan ([andrew.mcshan@chemistry.gatech.edu](mailto:andrew.mcshan@chemistry.gatech.edu))

Table of Contents:

Supplementary Figures start from page S2

**
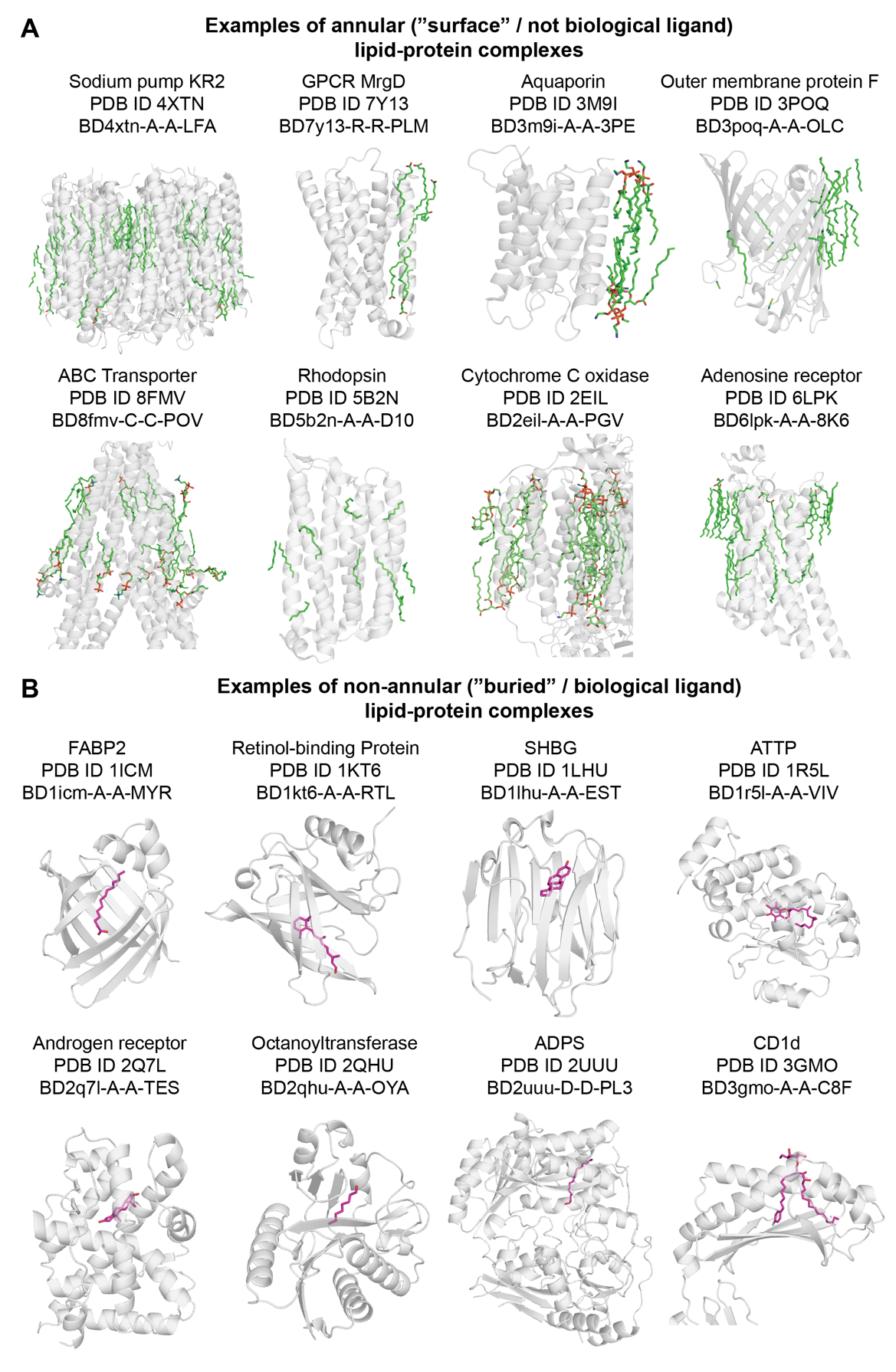
**

**Figure S1.** Representative examples of A. annular (surface, not biological ligands) and B. non-annular (buried, biological ligand) lipid-protein interactions. Annular lipids are shown as green sticks. Non-annular lipids are shown as magenta sticks. Proteins are shown as gray cartoon. The PDB ID code and the BioDolphin ID for each example is given.

**Figure S2.** Lipid-protein interaction profiles as a function of subcellular localization. Normalized number of interactions for lipid-protein complexes across each subcellular location defined by GO Cellular Component identification number. Interaction counts were normalized by dividing counts of each interaction type by the highest count. The number of PDB entries analyzed for each subcellular location is denoted by the n value. Only the top 10 abundant subcellular localizations are plotted for clarity.

**Figure S2 continued.** Lipid-protein interaction profiles as a function of subcellular localization. Normalized number of interactions for lipid-protein complexes across each subcellular location defined by GO Cellular Component identification number. Interaction counts were normalized by dividing counts of each interaction type by the highest count. The number of PDB entries analyzed for each subcellular location is denoted by the n value. Only the top 10 abundant subcellular localizations are plotted for clarity.

**Figure S2 continued.** Lipid-protein interaction profiles as a function of subcellular localization. Normalized number of interactions for lipid-protein complexes across each subcellular location defined by GO Cellular Component identification number. Interaction counts were normalized by dividing counts of each interaction type by the highest count. The number of PDB entries analyzed for each subcellular location is denoted by the n value. Only the top 10 abundant subcellular localizations are plotted for clarity.

**Figure S2 continued.** Lipid-protein interaction profiles as a function of subcellular localization. Normalized number of interactions for lipid-protein complexes across each subcellular location defined by GO Cellular Component identification number. Interaction counts were normalized by dividing counts of each interaction type by the highest count. The number of PDB entries analyzed for each subcellular location is denoted by the n value. Only the top 10 abundant subcellular localizations are plotted for clarity.

**Figure S2 continued.** Lipid-protein interaction profiles as a function of subcellular localization. Normalized number of interactions for lipid-protein complexes across each subcellular location defined by GO Cellular Component identification number. Interaction counts were normalized by dividing counts of each interaction type by the highest count. The number of PDB entries analyzed for each subcellular location is denoted by the n value. Only the top 10 abundant subcellular localizations are plotted for clarity.

**Figure S2 continued.** Lipid-protein interaction profiles as a function of subcellular localization. Normalized number of interactions for lipid-protein complexes across each subcellular location defined by GO Cellular Component identification number. Interaction counts were normalized by dividing counts of each interaction type by the highest count. The number of PDB entries analyzed for each subcellular location is denoted by the n value. Only the top 10 abundant subcellular localizations are plotted for clarity.

**Figure S2 continued.** Lipid-protein interaction profiles as a function of subcellular localization. Normalized number of interactions for lipid-protein complexes across each subcellular location defined by GO Cellular Component identification number. Interaction counts were normalized by dividing counts of each interaction type by the highest count. The number of PDB entries analyzed for each subcellular location is denoted by the n value. Only the top 10 abundant subcellular localizations are plotted for clarity.

**Figure S2 continued.** Lipid-protein interaction profiles as a function of subcellular localization. Normalized number of interactions for lipid-protein complexes across each subcellular location defined by GO Cellular Component identification number. Interaction counts were normalized by dividing counts of each interaction type by the highest count. The number of PDB entries analyzed for each subcellular location is denoted by the n value. Only the top 10 abundant subcellular localizations are plotted for clarity.


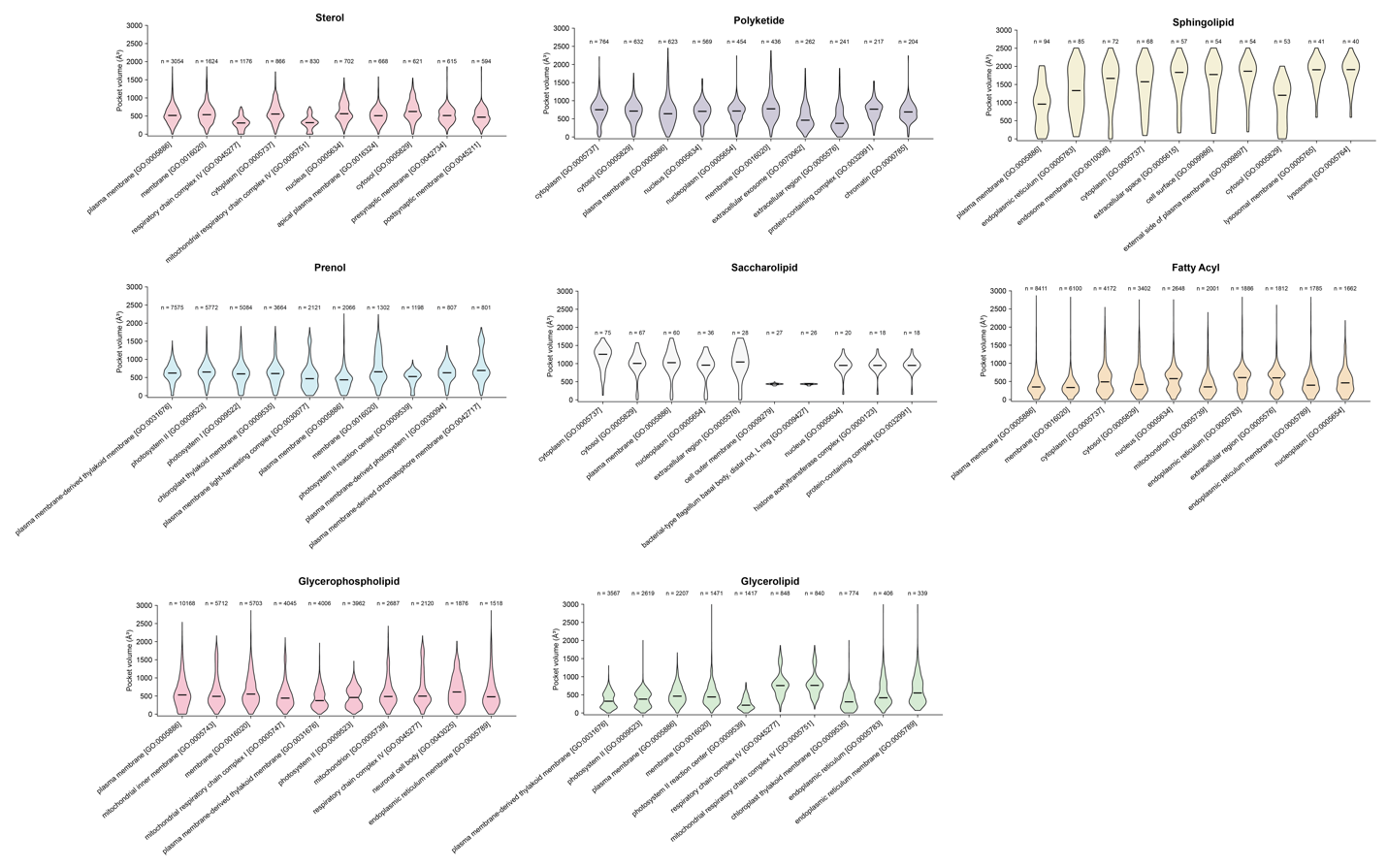


**Figure S3.** Results from dpocket analysis of protein pockets containing lipids classified within the eight lipid classes based on their subcellular location. Violin plots of dpocket descriptors are shown with median values represented as horizontal black lines. The number of lipid-bound protein pockets analyzed for each lipid class for each subcellular location (n) is provided. Subcellular location is defined in Gene Ontology (GO) using the Cellular Component (GO-CC) ontology obtained from UniProt. For clarity *p*-values are not plotted but were calculated and are provided in files located on the GitHub link associated with the manuscript. Pairwise differences between pocket features between lipid classes were assessed using a two-sided Mann–Whitney U test. *p*-values were adjusted for multiple comparisons using the Benjamini–Hochberg procedure (FDR < 0.05) as implemented in statsmodels. ns = not significant. * = *p* < 0.05 ; ** = *p* < 0.01 ; *** = *p* < 0.001.


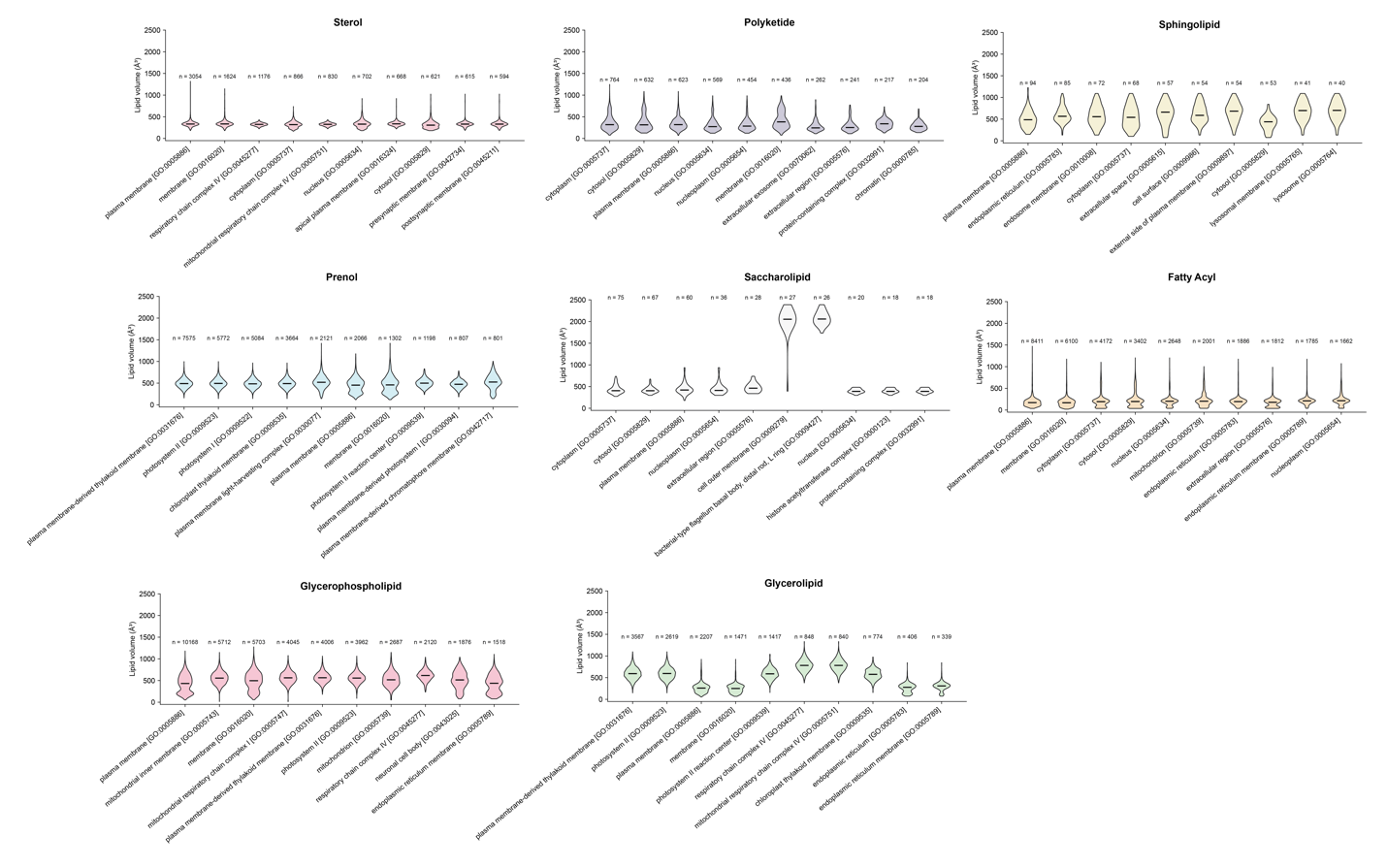


**Figure S3 continued.** Results from dpocket analysis of protein pockets containing lipids classified within the eight lipid classes based on their subcellular location. Violin plots of dpocket descriptors are shown with median values represented as horizontal black lines. The number of lipid-bound protein pockets analyzed for each lipid class for each subcellular location (n) is provided. Subcellular location is defined in Gene Ontology (GO) using the Cellular Component (GO-CC) ontology obtained from UniProt. For clarity *p*-values are not plotted but were calculated and are provided in files located on the GitHub link associated with the manuscript. Pairwise differences between pocket features between lipid classes were assessed using a two-sided Mann–Whitney U test. *p*-values were adjusted for multiple comparisons using the Benjamini–Hochberg procedure (FDR < 0.05) as implemented in statsmodels. ns = not significant. * = *p* < 0.05 ; ** = *p* < 0.01 ; *** = *p* < 0.001.


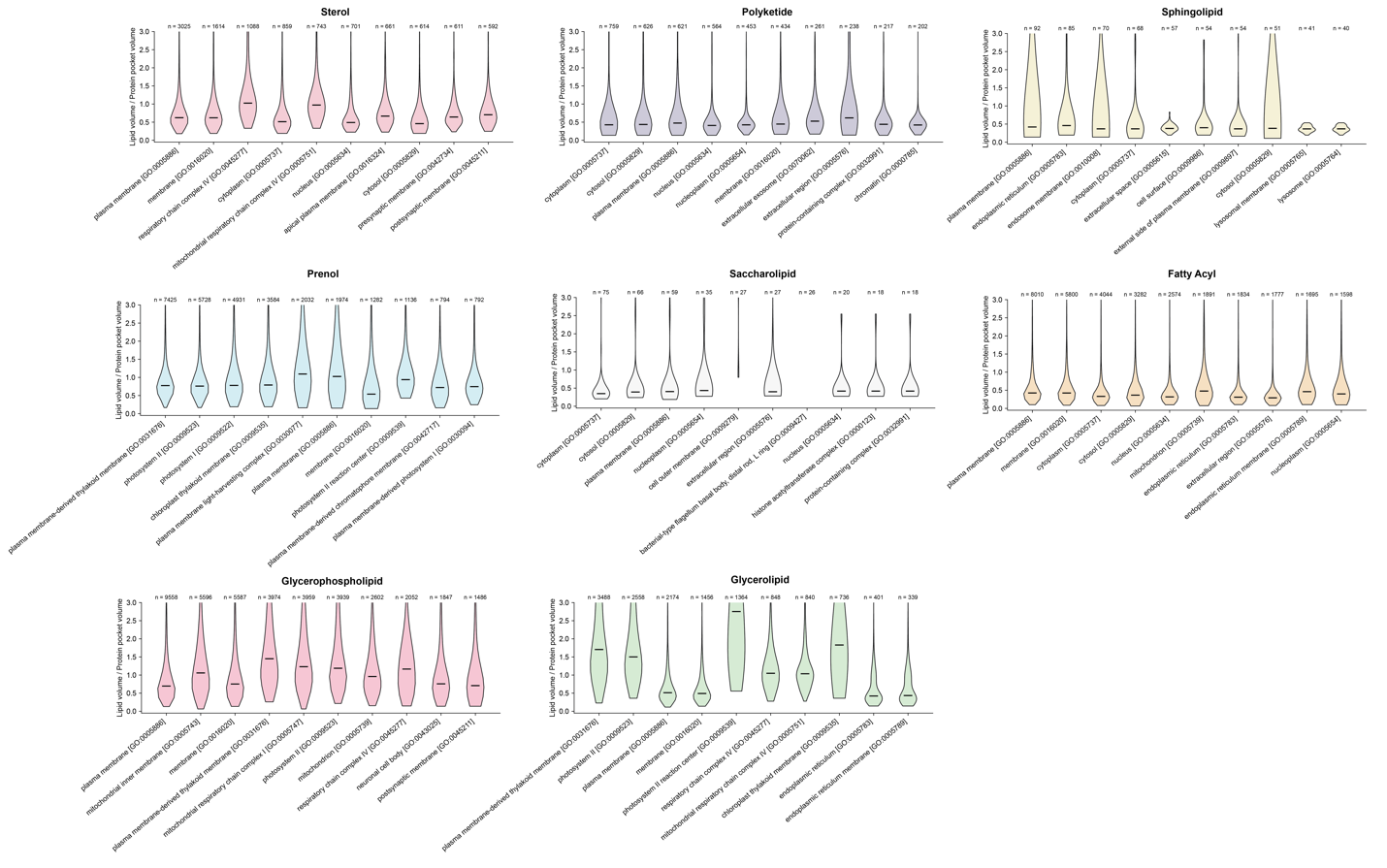


**Figure S3 continued.** Results from dpocket analysis of protein pockets containing lipids classified within the eight lipid classes based on their subcellular location. Violin plots of dpocket descriptors are shown with median values represented as horizontal black lines. The number of lipid-bound protein pockets analyzed for each lipid class for each subcellular location (n) is provided. Subcellular location is defined in Gene Ontology (GO) using the Cellular Component (GO-CC) ontology obtained from UniProt. For clarity *p*-values are not plotted but were calculated and are provided in files located on the GitHub link associated with the manuscript. Pairwise differences between pocket features between lipid classes were assessed using a two-sided Mann–Whitney U test. *p*-values were adjusted for multiple comparisons using the Benjamini–Hochberg procedure (FDR < 0.05) as implemented in statsmodels. ns = not significant. * = *p* < 0.05 ; ** = *p* < 0.01 ; *** = *p* < 0.001.


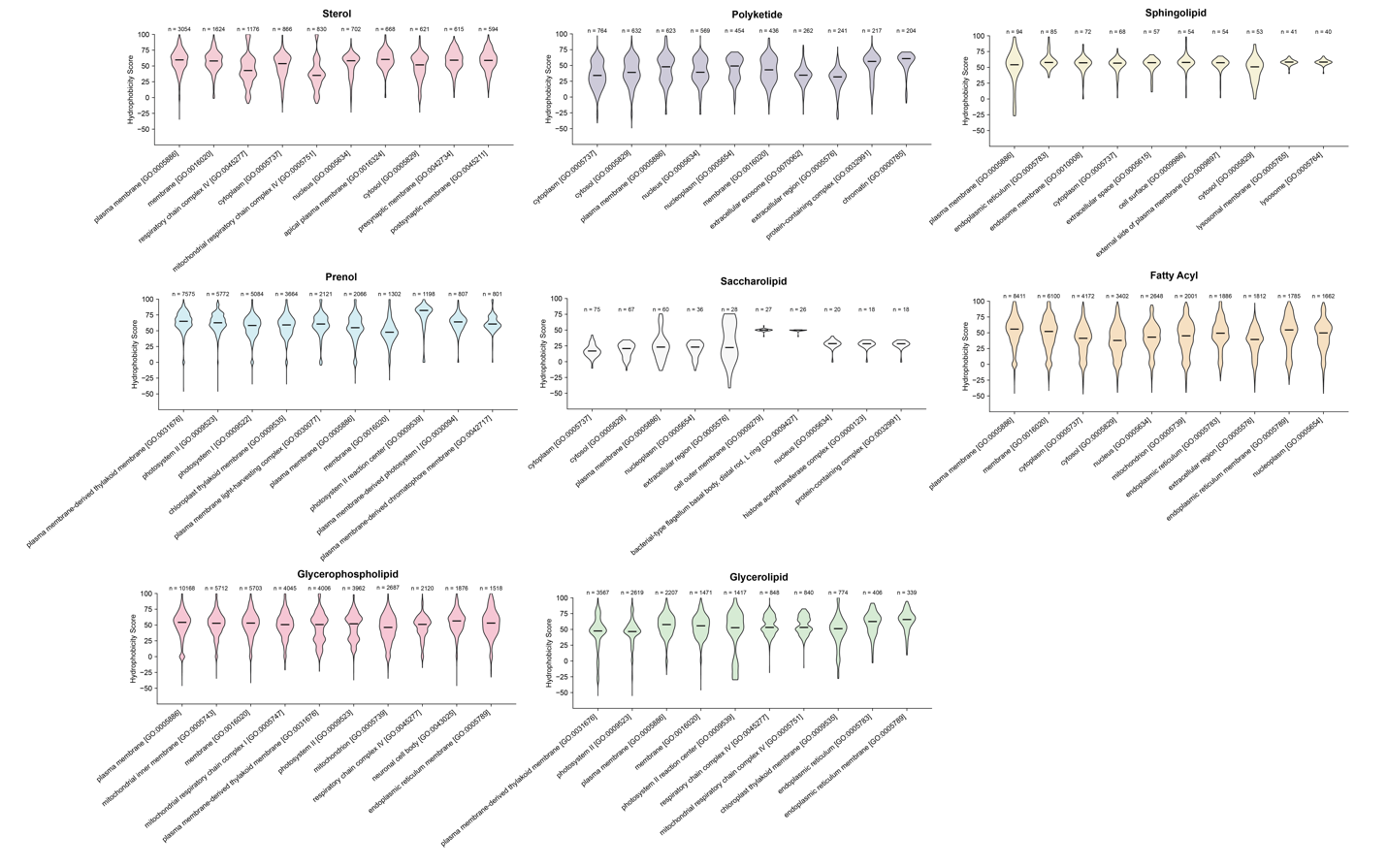


**Figure S3 continued.** Results from dpocket analysis of protein pockets containing lipids classified within the eight lipid classes based on their subcellular location. Violin plots of dpocket descriptors are shown with median values represented as horizontal black lines. The number of lipid-bound protein pockets analyzed for each lipid class for each subcellular location (n) is provided. Subcellular location is defined in Gene Ontology (GO) using the Cellular Component (GO-CC) ontology obtained from UniProt. For clarity *p*-values are not plotted but were calculated and are provided in files located on the GitHub link associated with the manuscript. Pairwise differences between pocket features between lipid classes were assessed using a two-sided Mann–Whitney U test. *p*-values were adjusted for multiple comparisons using the Benjamini–Hochberg procedure (FDR < 0.05) as implemented in statsmodels. ns = not significant. * = *p* < 0.05 ; ** = *p* < 0.01 ; *** = *p* < 0.001.


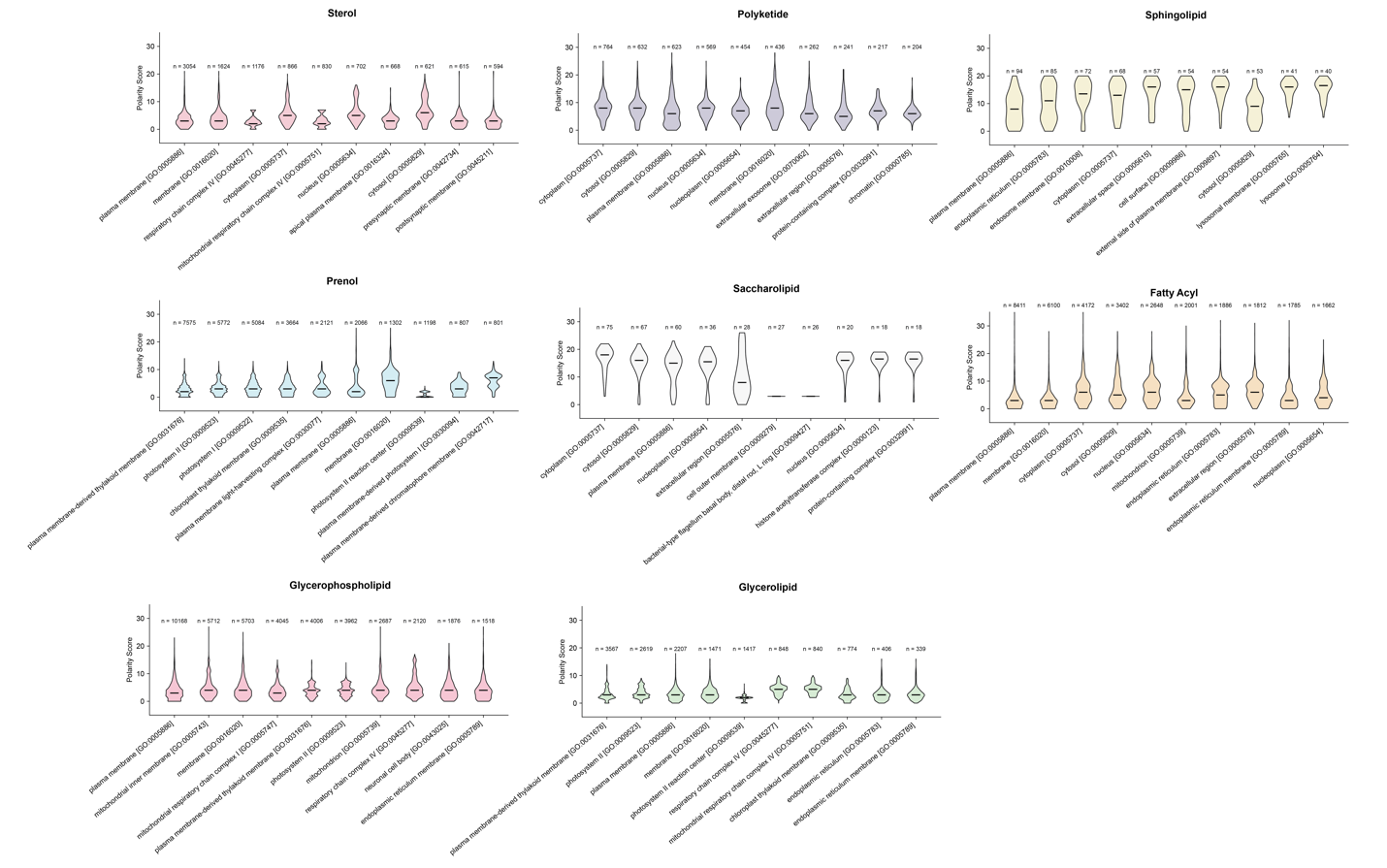


**Figure S3 continued.** Results from dpocket analysis of protein pockets containing lipids classified within the eight lipid classes based on their subcellular location. Violin plots of dpocket descriptors are shown with median values represented as horizontal black lines. The number of lipid-bound protein pockets analyzed for each lipid class for each subcellular location (n) is provided. Subcellular location is defined in Gene Ontology (GO) using the Cellular Component (GO-CC) ontology obtained from UniProt. For clarity *p*-values are not plotted but were calculated and are provided in files located on the GitHub link associated with the manuscript. Pairwise differences between pocket features between lipid classes were assessed using a two-sided Mann–Whitney U test. *p*-values were adjusted for multiple comparisons using the Benjamini–Hochberg procedure (FDR < 0.05) as implemented in statsmodels. ns = not significant. * = *p* < 0.05 ; ** = *p* < 0.01 ; *** = *p* < 0.001.


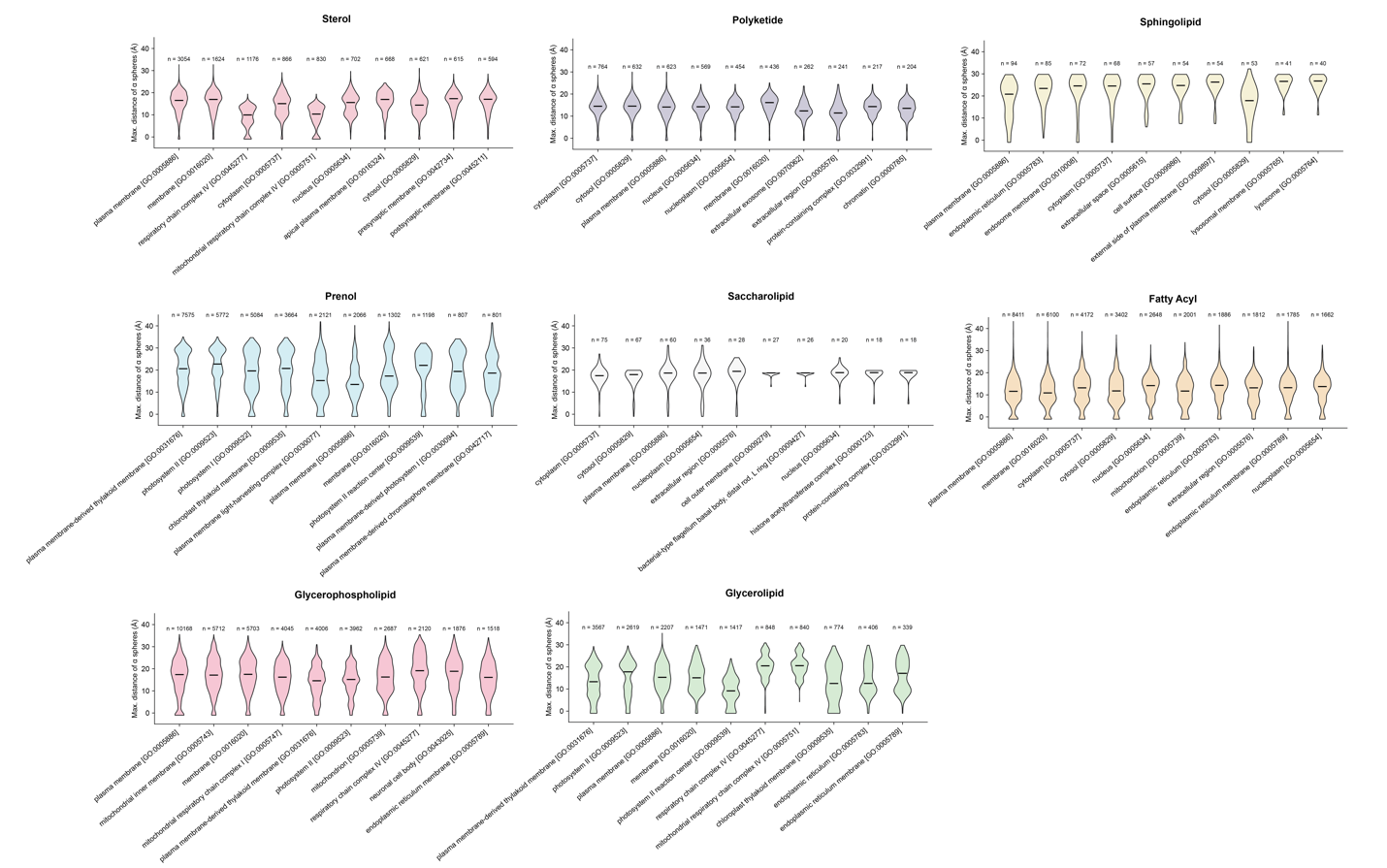


**Figure S3 continued.** Results from dpocket analysis of protein pockets containing lipids classified within the eight lipid classes based on their subcellular location. Violin plots of dpocket descriptors are shown with median values represented as horizontal black lines. The number of lipid-bound protein pockets analyzed for each lipid class for each subcellular location (n) is provided. Subcellular location is defined in Gene Ontology (GO) using the Cellular Component (GO-CC) ontology obtained from UniProt. For clarity *p*-values are not plotted but were calculated and are provided in files located on the GitHub link associated with the manuscript. Pairwise differences between pocket features between lipid classes were assessed using a two-sided Mann–Whitney U test. *p*-values were adjusted for multiple comparisons using the Benjamini–Hochberg procedure (FDR < 0.05) as implemented in statsmodels. ns = not significant. * = *p* < 0.05 ; ** = *p* < 0.01 ; *** = *p* < 0.001.


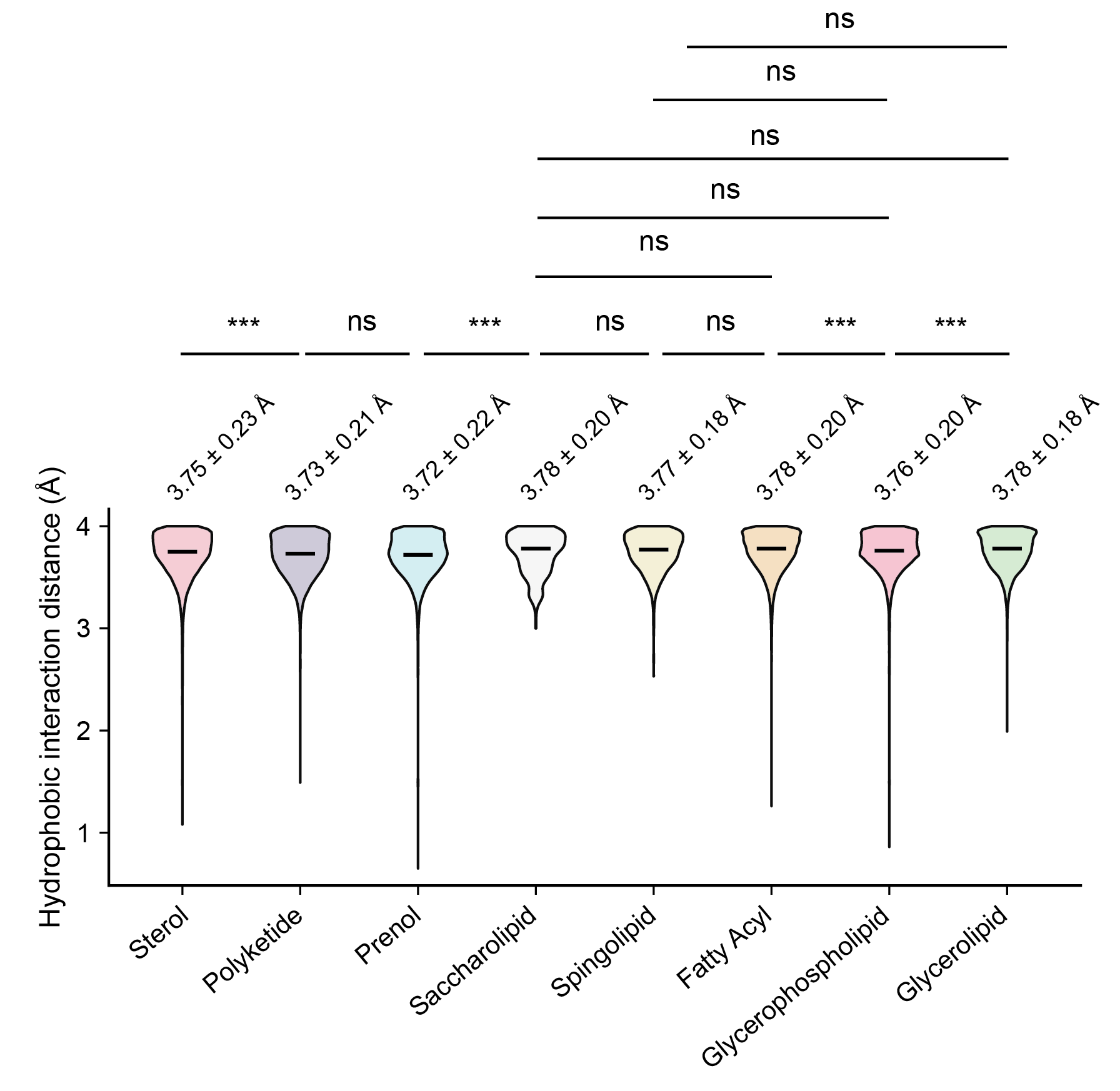


**Figure S4.** **Distances of hydrophobic interactions across lipid-protein complexes in BioDolphin.** Distances of hydrophobic interactions in lipid-protein complexes across the eight classes of lipids. Hydrophobic interactions and their distances were obtained from a PLIP analysis of all 113,782 entries in the BioDolphin database. The median ± standard deviation hydrophobic interaction distance is shown for each class of lipid. Pairwise differences between hydrophobic interaction distances between lipid classes were assessed using a two-sided Mann–Whitney U test. *p*-values were adjusted for multiple comparisons using the Benjamini–Hochberg procedure (FDR < 0.05) as implemented in statsmodels. ns = not significant. * = *p* < 0.05 ; ** = *p* < 0.01 ; *** = *p* < 0.001. Statistical significance (*, **, or ***) is denoted only for select datasets for clarity. All not significant datasets are denoted.


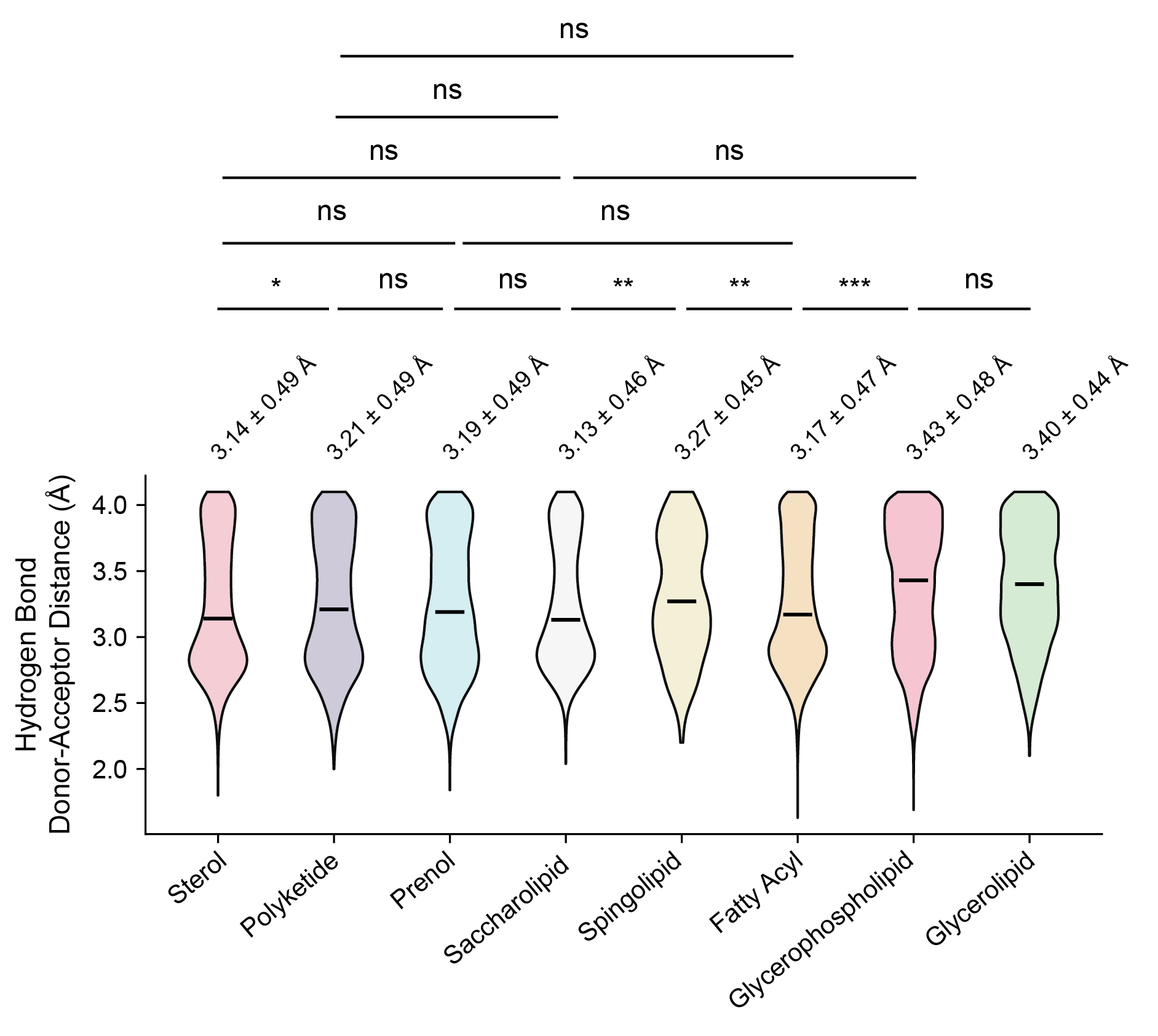


**Figure S5.** **Distance between donor and acceptor atoms in hydrogen bonds across lipid-protein complexes in BioDolphin.** Distances between hydrogen bond donor and acceptor atoms (“DIST_D-A”) in lipid-protein complexes across the eight classes of lipids. Distances were obtained from a PLIP analysis of all 113,782 entries in the BioDolphin database. The median ± standard deviation D-A distance is shown for each class of lipid. Pairwise differences between hydrogen bond donor and acceptor atom distances between lipid classes were assessed using a two-sided Mann–Whitney U test. *p*-values were adjusted for multiple comparisons using the Benjamini–Hochberg procedure (FDR < 0.05) as implemented in statsmodels. ns = not significant. * = *p* < 0.05 ; ** = *p* < 0.01 ; *** = *p* < 0.001. Statistical significance (*, **, or ***) is denoted only for select datasets for clarity. All not significant datasets are denoted.


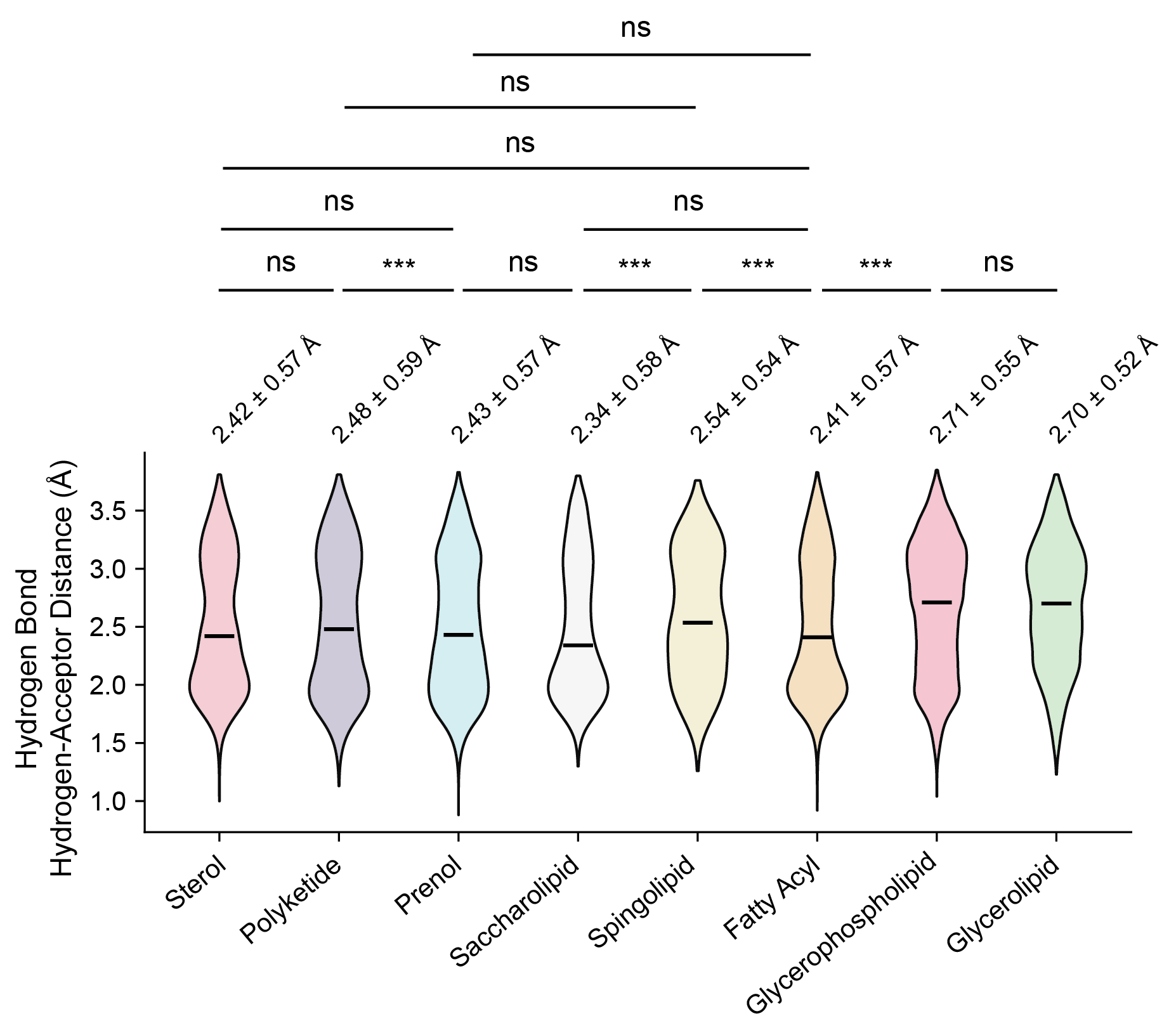


**Figure S6.** **Distance between hydrogen and acceptor atoms in hydrogen bonds across lipid-protein complexes in BioDolphin.** Distance between hydrogen bond hydrogen and acceptor atom (“DIST_H-A”) in lipid-protein complexes across the eight classes of lipids. Distances were obtained from a PLIP analysis of all 113,782 entries in the BioDolphin database. The median ± standard deviation H-A distance is shown for each class of lipid. Pairwise differences between hydrogen bond hydrogen and acceptor atom distances between lipid classes were assessed using a two-sided Mann–Whitney U test. *p*-values were adjusted for multiple comparisons using the Benjamini–Hochberg procedure (FDR < 0.05) as implemented in statsmodels. ns = not significant. * = *p* < 0.05 ; ** = *p* < 0.01 ; *** = *p* < 0.001. Statistical significance (*, **, or ***) is denoted only for select datasets for clarity. All not significant datasets are denoted.

**
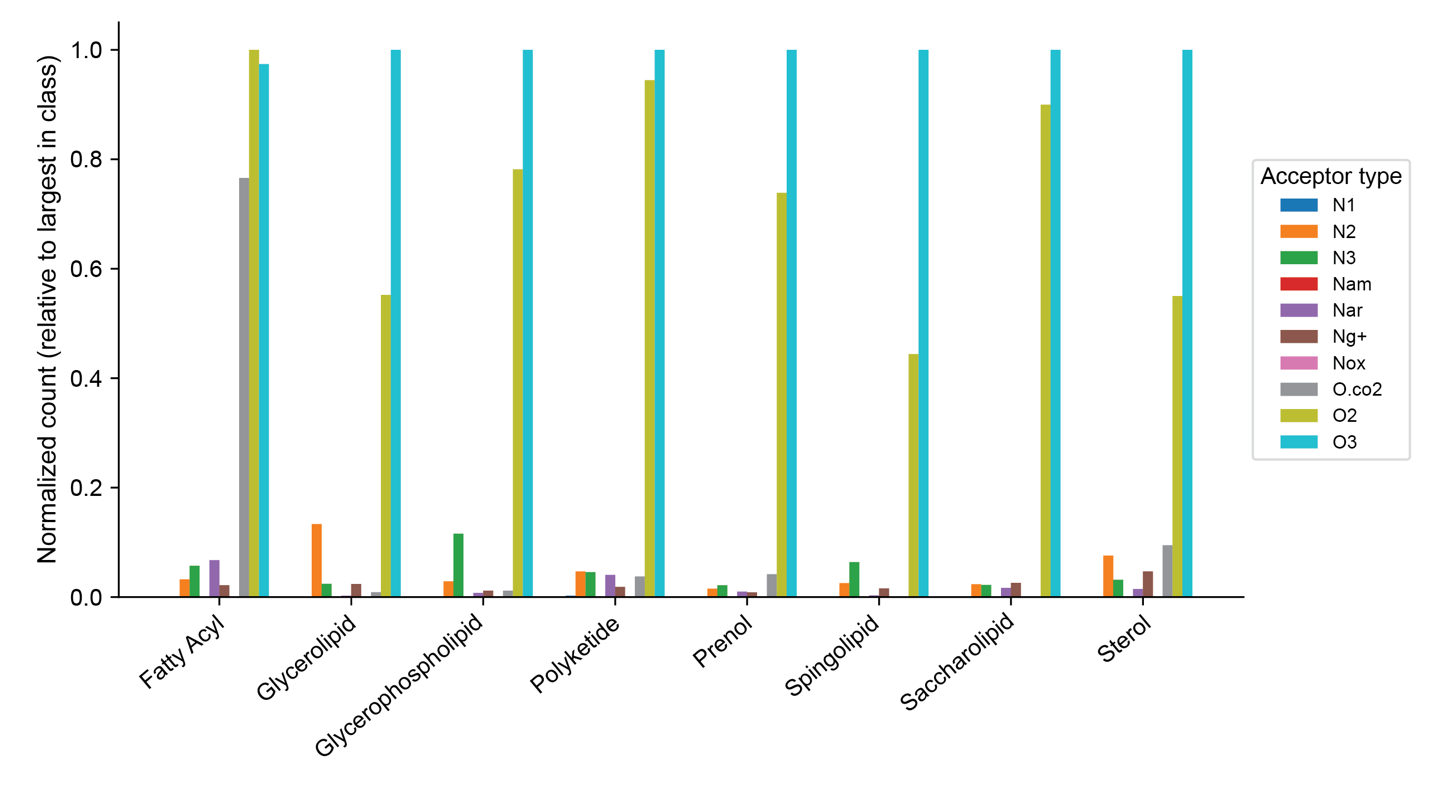
**

**Figure S7.** **Acceptor atom types in hydrogen bonds across lipid-protein complexes in BioDolphin.** Acceptor atom types (“ACCEPTORTYPE”) in lipid-protein complexes across the eight classes of lipids. Acceptor atom types were obtained and normalized (relative to the highest count in each lipid class) from a PLIP analysis of all 113,782 entries in the BioDolphin database. The acceptor atom type names correspond to Open Babel's atom typing system that uses SMARTS patterns in a configuration file (atomtyp.txt) as follows: N1 - sp1 hybridized nitrogen (terminal, i.e., nitriles); N2 - sp2 hybridized nitrogen (double-bonded, i.e., imines); N3 - sp3 hybridized nitrogen (tetrahedral, i.e., amines); Nam - Nitrogen in amide group (i.e., N-C=O); Nar - aromatic nitrogen; Ng+ - positively charged nitrogen in generic conjugated system; Nox - nitrogen bonded to oxygen (i.e., nitroso); O.co2 - carboxylate oxygen (i.e., -COO⁻); O2 - sp2 hybridized oxygen (i.e., carbonyl); O3 - sp3 hybridized oxygen (i.e., hydroxyl).


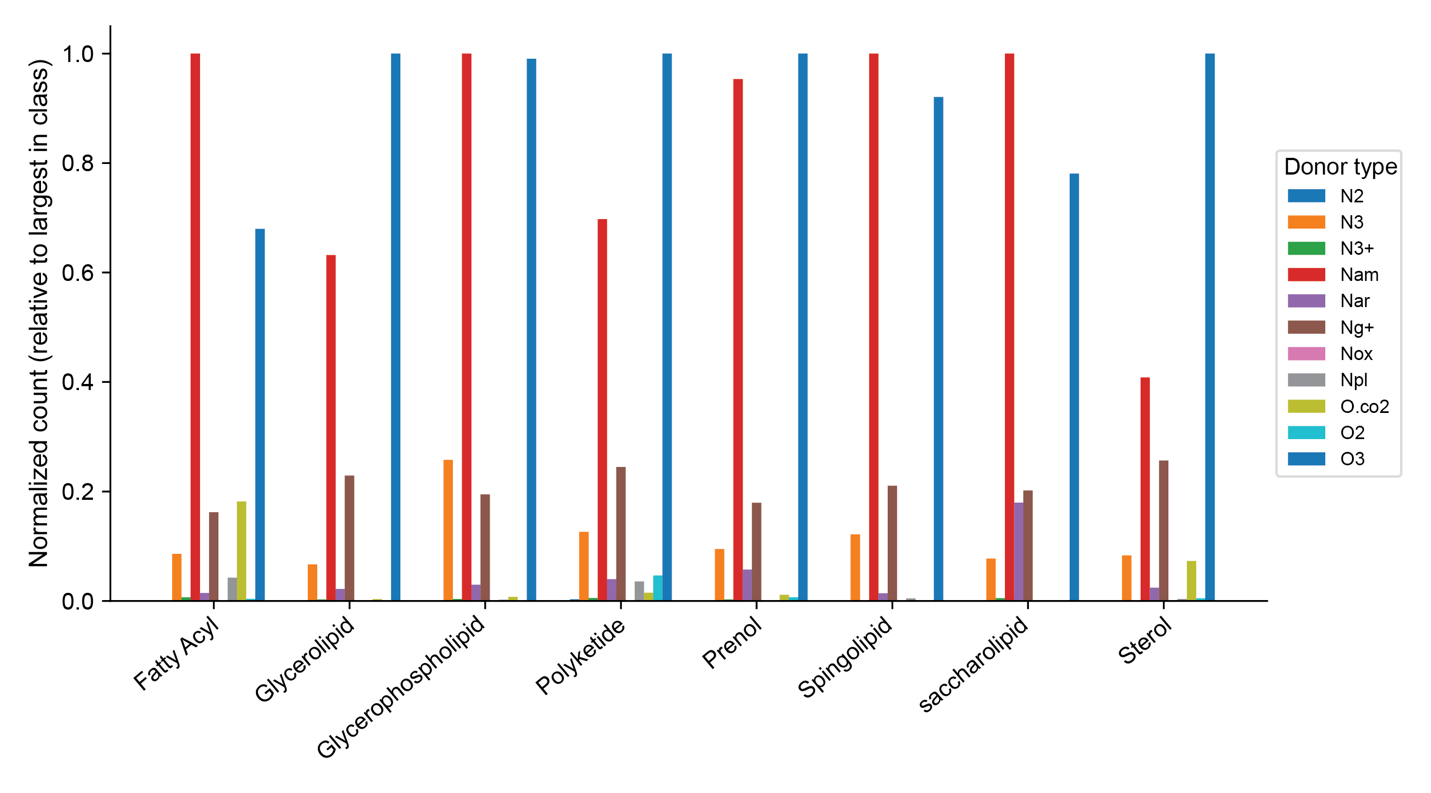


**Figure S8.** **Donor atom types in hydrogen bonds across lipid-protein complexes in BioDolphin.** Donor atom types (“DONORTYPE”) in lipid-protein complexes across the eight classes of lipids. Donor atom types were obtained and normalized (relative to the highest count in each lipid class) from a PLIP analysis of all 113,782 entries in the BioDolphin database. The donor atom type names correspond to Open Babel's atom typing system that uses SMARTS patterns in a configuration file (atomtyp.txt) as follows: N2 - sp2 hybridized nitrogen (double-bonded, i.e., imines); N3 - sp3 hybridized nitrogen (tetrahedral, i.e., amines); N3+ - positively charged sp³ nitrogen (tetrahedral, i.e., ammonium-like nitrogen); Nam - Nitrogen in amide group (i.e., N-C=O); Nar - aromatic nitrogen; Ng+ - positively charged nitrogen in generic conjugated system; Nox - nitrogen bonded to oxygen (i.e., nitroso); Npl - planar sp2 nitrogen (i.e., pyrrole); O.co2 - carboxylate oxygen (i.e., -COO⁻); O2 - sp2 hybridized oxygen (i.e., carbonyl); O3 - sp3 hybridized oxygen (i.e., hydroxyl).


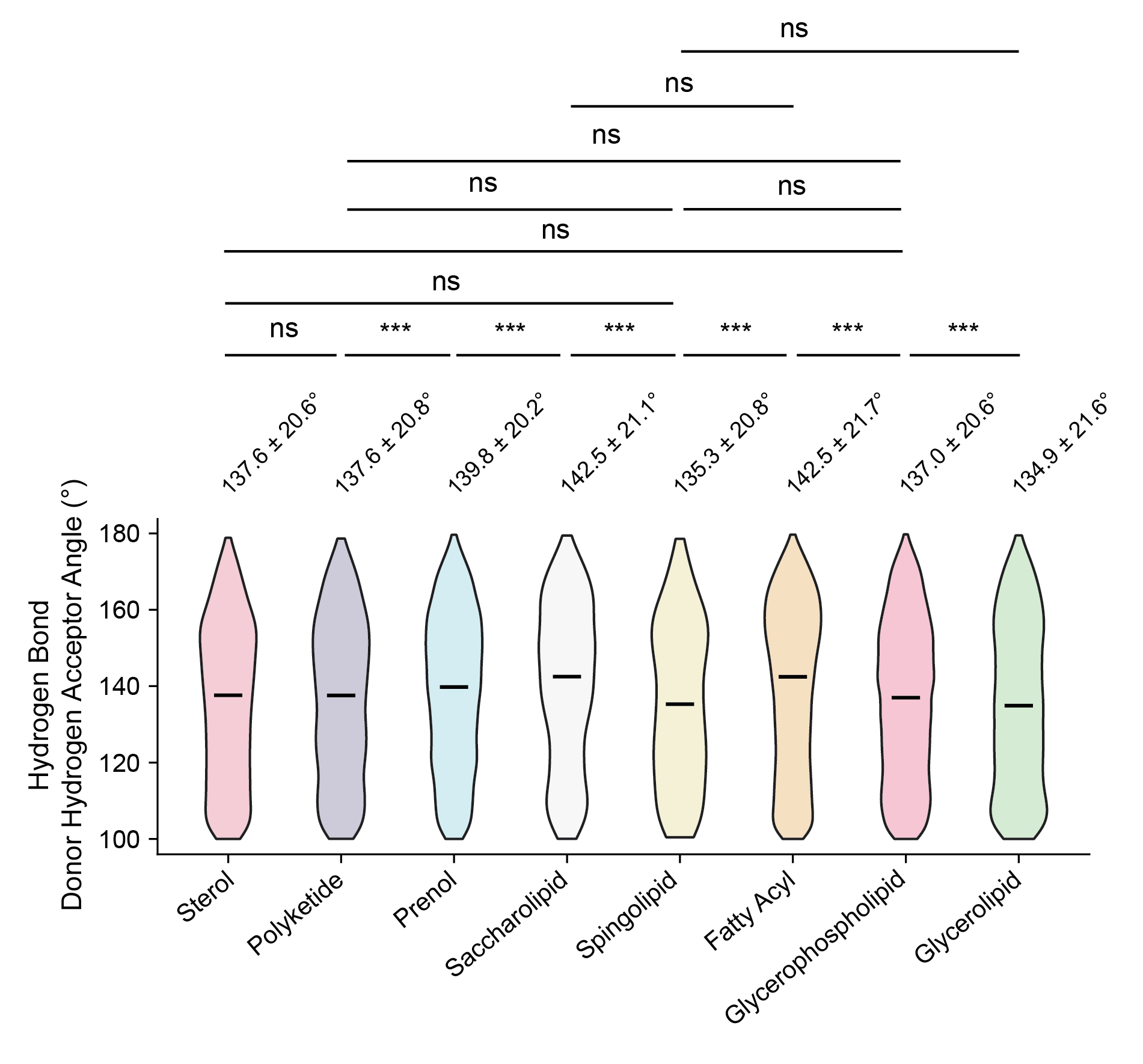


**Figure S9.** **Donor angle in hydrogen bonds across lipid-protein complexes in BioDolphin.** Angle at the donor atom of hydrogen bonds (“DON_ANGLE”) in lipid-protein complexes across the eight classes of lipids. Donor angles were obtained from a PLIP analysis of all 113,782 entries in the BioDolphin database. The median ± standard deviation donor angle is shown for each class of lipid. PLIP uses a 100 ° minimum angle at the hydrogen bond donor (D-H...A). Pairwise differences between hydrogen bond donor angles between lipid classes were assessed using a two-sided Mann–Whitney U test. *p*-values were adjusted for multiple comparisons using the Benjamini–Hochberg procedure (FDR < 0.05) as implemented in statsmodels. ns = not significant. * = *p* < 0.05 ; ** = *p* < 0.01 ; *** = *p* < 0.001. Statistical significance (*, **, or ***) is denoted only for select datasets for clarity. All not significant datasets are denoted.


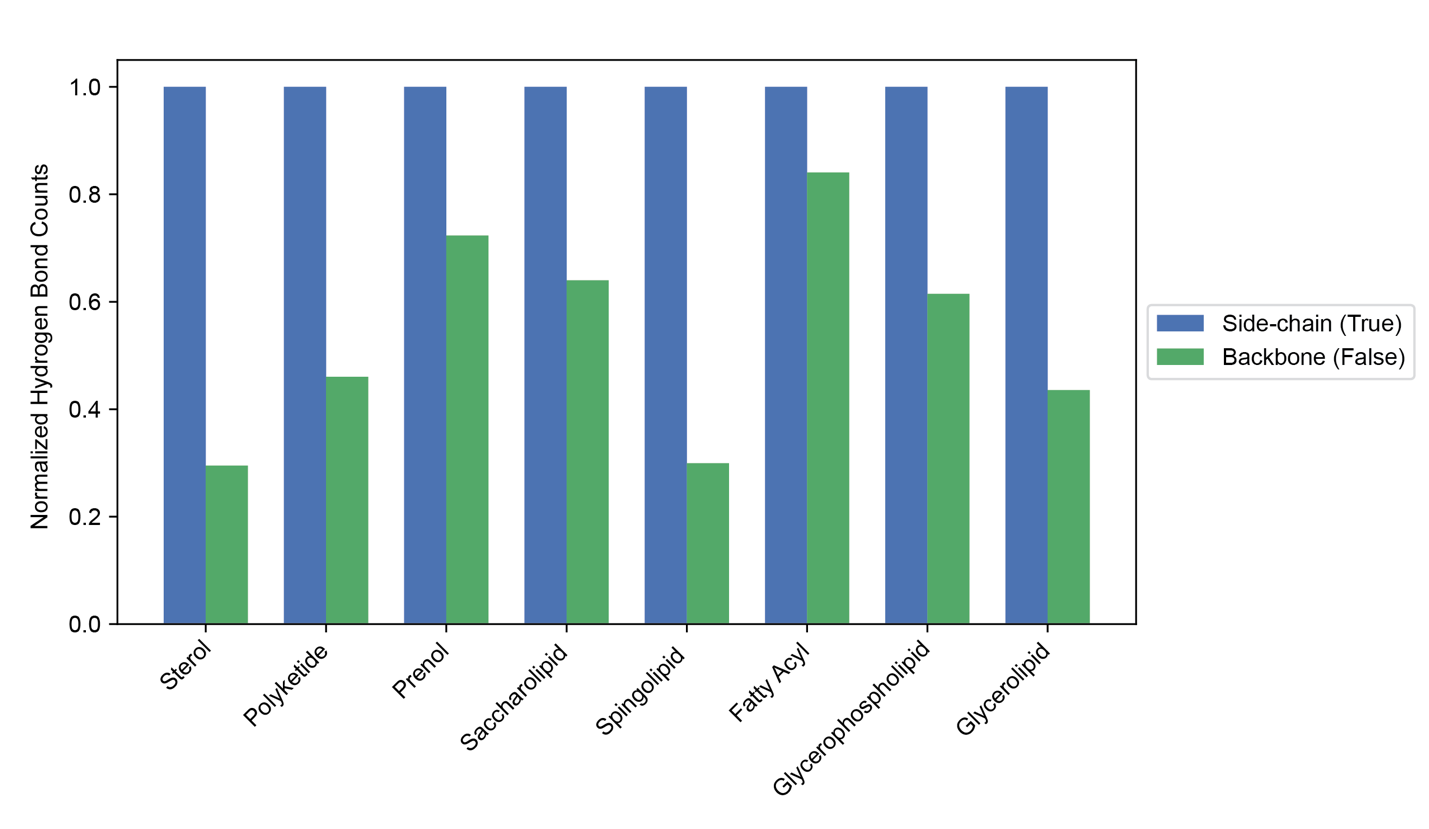


**Figure S10.** **Characterizing hydrogen bonds of lipid atoms to side-chain vs backbone atoms of proteins across lipid-protein complexes in BioDolphin.** Characterizing the location of hydrogen bonds (side-chain or not) between lipid atoms and protein atoms (“SIDECHAIN”) in lipid-protein complexes across the eight classes of lipids. The identity of the hydrogen bond (side-chain or backbone atoms) from a PLIP analysis of all 113,782 entries in the BioDolphin database. Counts were normalized relative to the highest count in each lipid class.


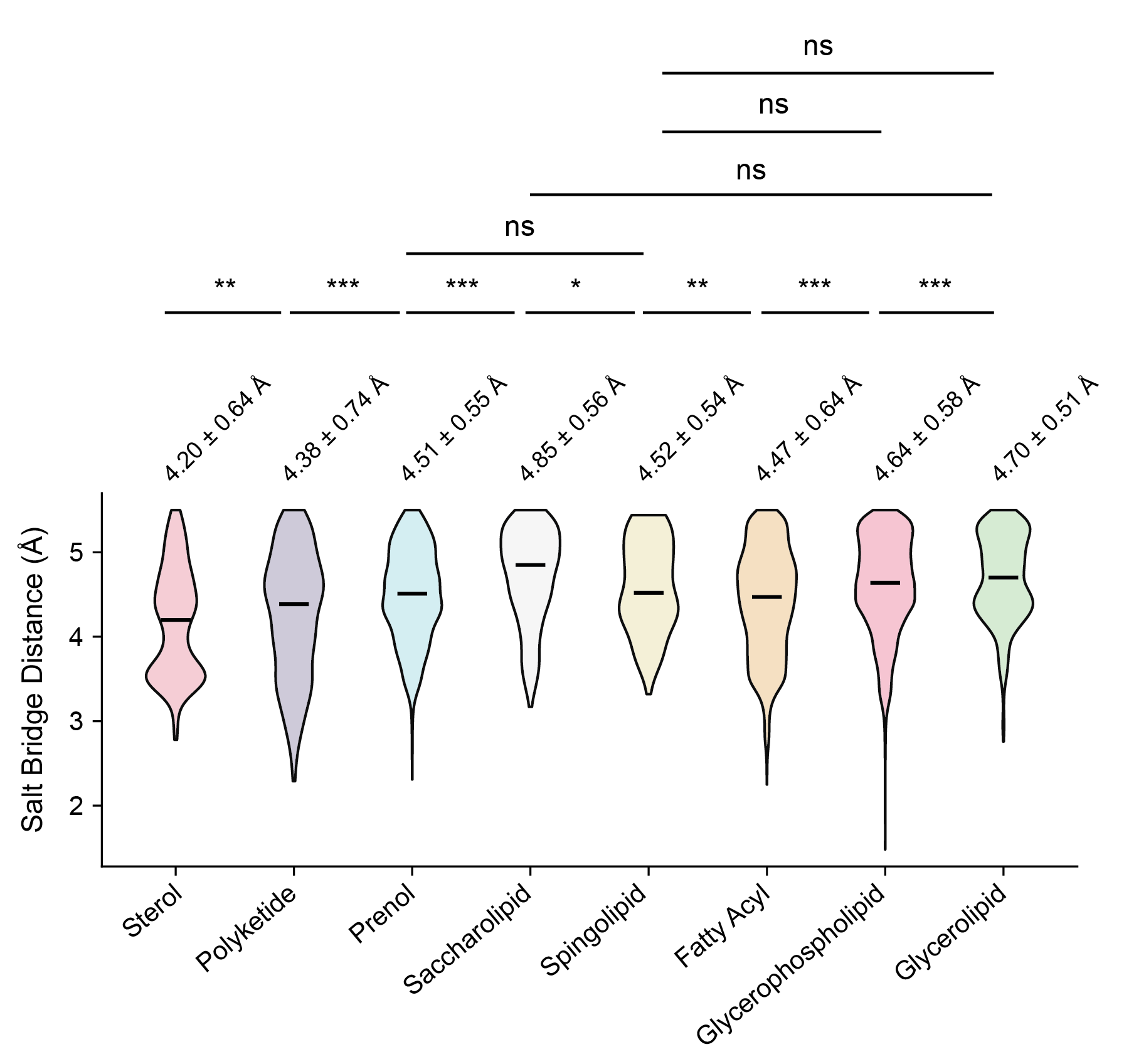


**Figure S11.** **Distances of salt bridge interactions across lipid-protein complexes in BioDolphin.** Distances of salt bridges in lipid-protein complexes across the eight classes of lipids. Salt bridge interactions and their distances were obtained from a PLIP analysis of all 113,782 entries in the BioDolphin database. The median ± standard deviation salt bridge interaction distance is shown for each class of lipid. Pairwise differences between salt bridge distances between lipid classes were assessed using a two-sided Mann–Whitney U test. *p*-values were adjusted for multiple comparisons using the Benjamini–Hochberg procedure (FDR < 0.05) as implemented in statsmodels. ns = not significant. * = *p* < 0.05 ; ** = *p* < 0.01 ; *** = *p* < 0.001. Statistical significance (*, **, or ***) is denoted only for select datasets for clarity. All not significant datasets are denoted.


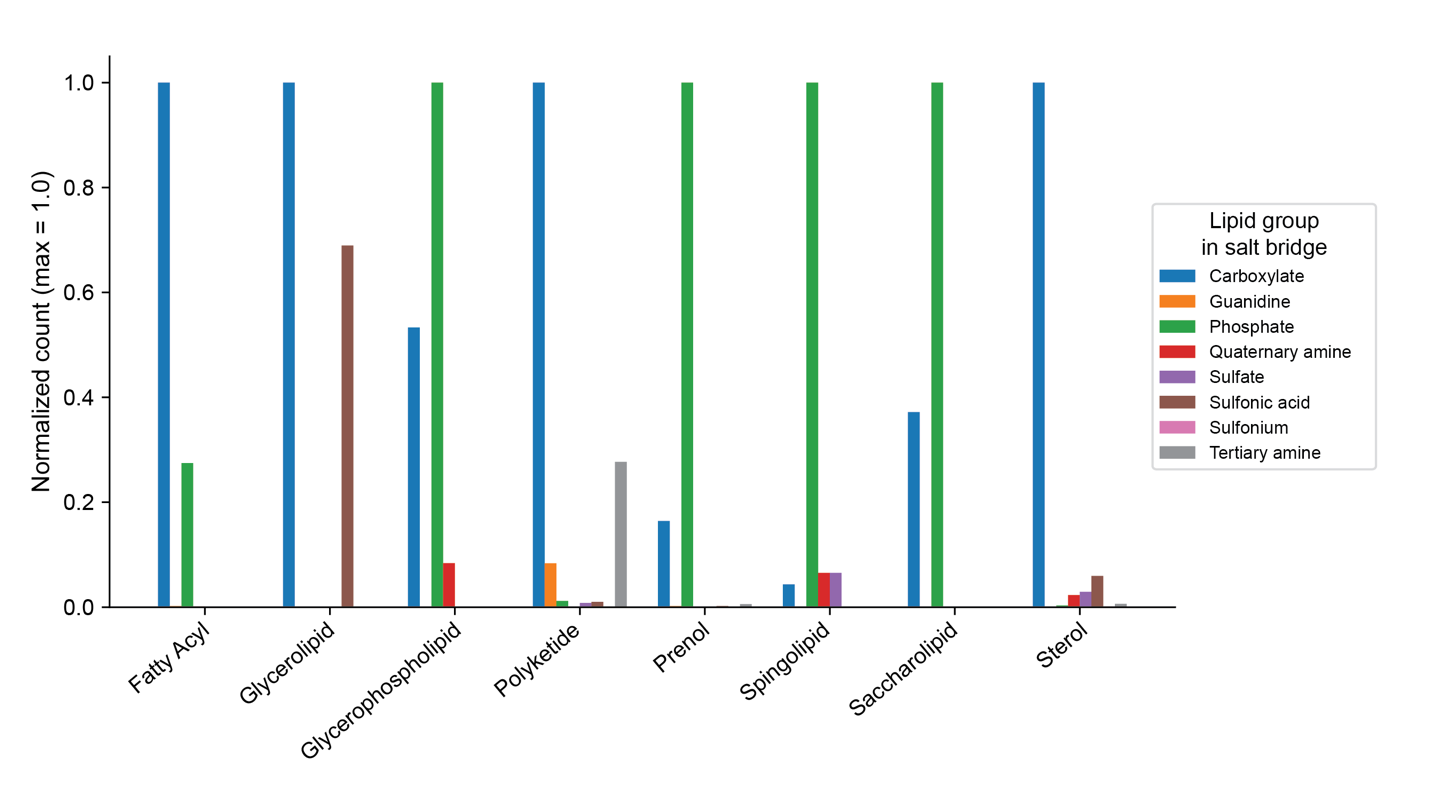


**Figure S12.** **Groups of lipids involved in salt bridge interactions across lipid-protein complexes in BioDolphin.** Lipid chemical group involved in salt bridges (“LIG_GROUP”) in lipid-protein complexes across the eight classes of lipids. Lipid groups involved in salt bridges were obtained and normalized (relative to the highest count in each lipid class) from a PLIP analysis of all 113,782 entries in the BioDolphin database.


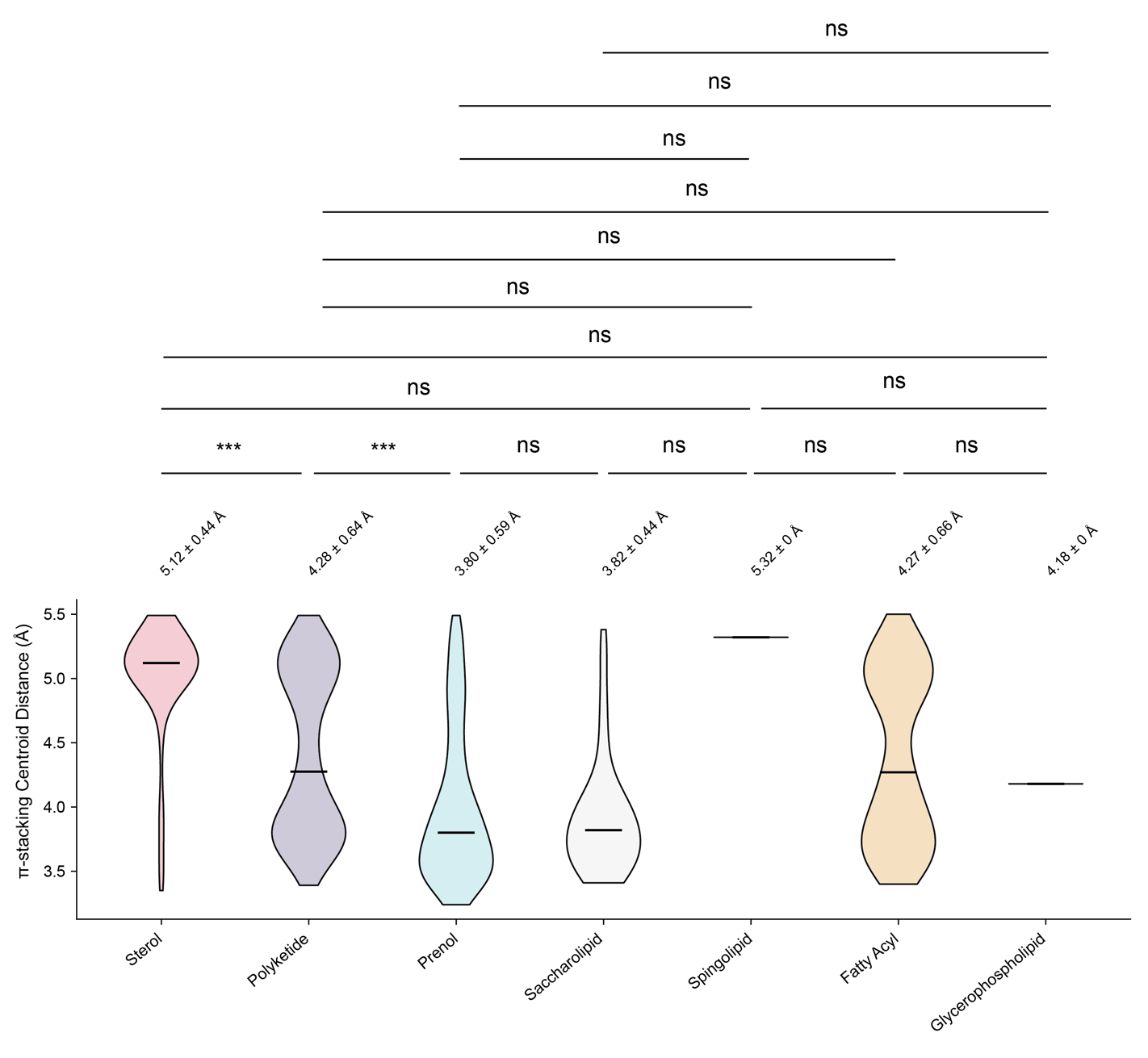


**Figure S13.** **Centroid distance for π-stacking interactions across lipid-protein complexes in BioDolphin.** Centroid distance for π-stacking interactions (“CENTDIST”) between lipid and protein atoms in lipid-protein complexes across the eight classes of lipids. π-stacking interactions and their centroid distance were obtained from a PLIP analysis of all 113,782 entries in the BioDolphin database. The median ± standard deviation π-stacking centroid distances is shown for each class of lipid. Note that PLIP did not identify any π-stacking interactions for glycerolipid-protein complexes. Pairwise differences between π-stacking centroid distances between lipid classes were assessed using a two-sided Mann–Whitney U test. *p*-values were adjusted for multiple comparisons using the Benjamini–Hochberg procedure (FDR < 0.05) as implemented in statsmodels. ns = not significant. * = *p* < 0.05 ; ** = *p* < 0.01 ; *** = *p* < 0.001. Statistical significance (*, **, or ***) is denoted only for select datasets for clarity. All not significant datasets are denoted.


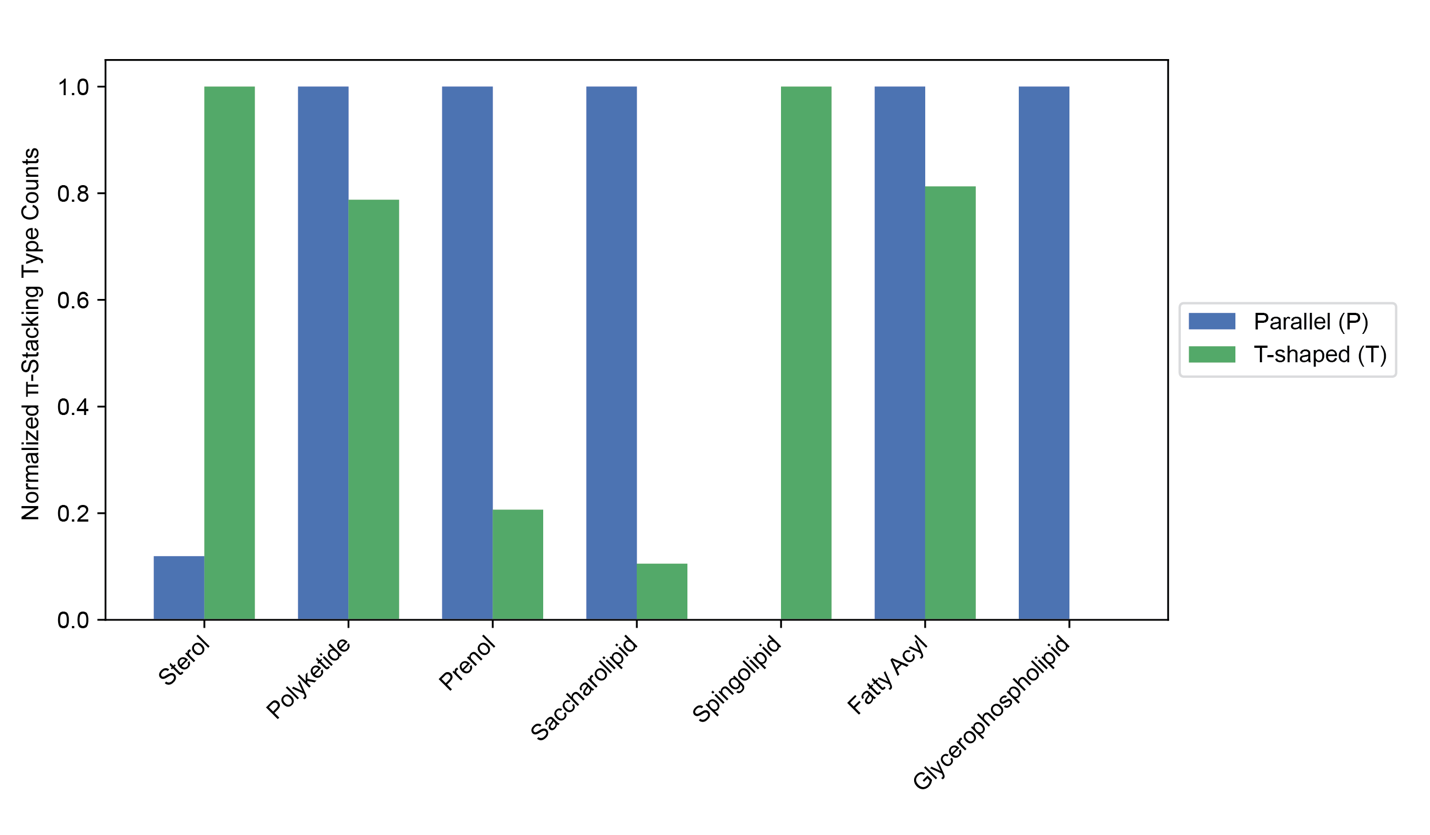


**Figure S14.** **Type of π-stacking interactions across lipid-protein complexes in BioDolphin.** Type of π-stacking interactions (“TYPE”) in lipid-protein complexes across the eight classes of lipids. π-stacking interactions were obtained and normalized (relative to the highest count in each lipid class) from a PLIP analysis of all 113,782 entries in the BioDolphin database. Note that PLIP did not identify any π-stacking interactions for glycerolipid-protein complexes.


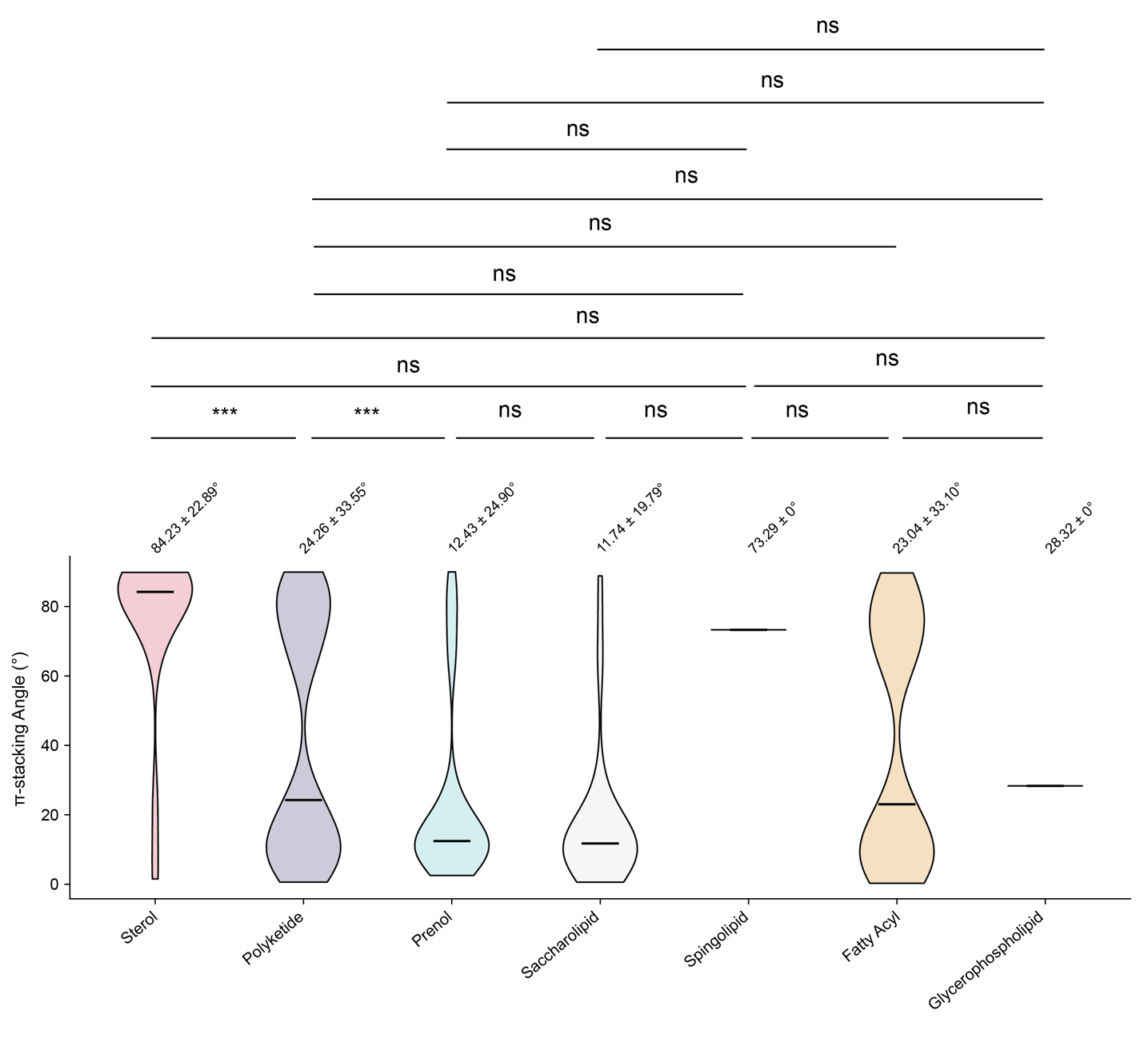


**Figure S15.** **Angle of π-stacking interactions across lipid-protein complexes in BioDolphin.** Angle of π-stacking interactions (“ANGLE”) between lipid and protein atoms in lipid-protein complexes across the eight classes of lipids. π-stacking interactions and their angles were obtained from a PLIP analysis of all 113,782 entries in the BioDolphin database. The median ± standard deviation π-stacking angle is shown for each class of lipid. Note that PLIP did not identify any π-stacking interactions for glycerolipid-protein complexes. Pairwise differences between π-stacking angles between lipid classes were assessed using a two-sided Mann–Whitney U test. *p*-values were adjusted for multiple comparisons using the Benjamini–Hochberg procedure (FDR < 0.05) as implemented in statsmodels. ns = not significant. * = *p* < 0.05 ; ** = *p* < 0.01 ; *** = *p* < 0.001. Statistical significance (*, **, or ***) is denoted only for select datasets for clarity. All not significant datasets are denoted.


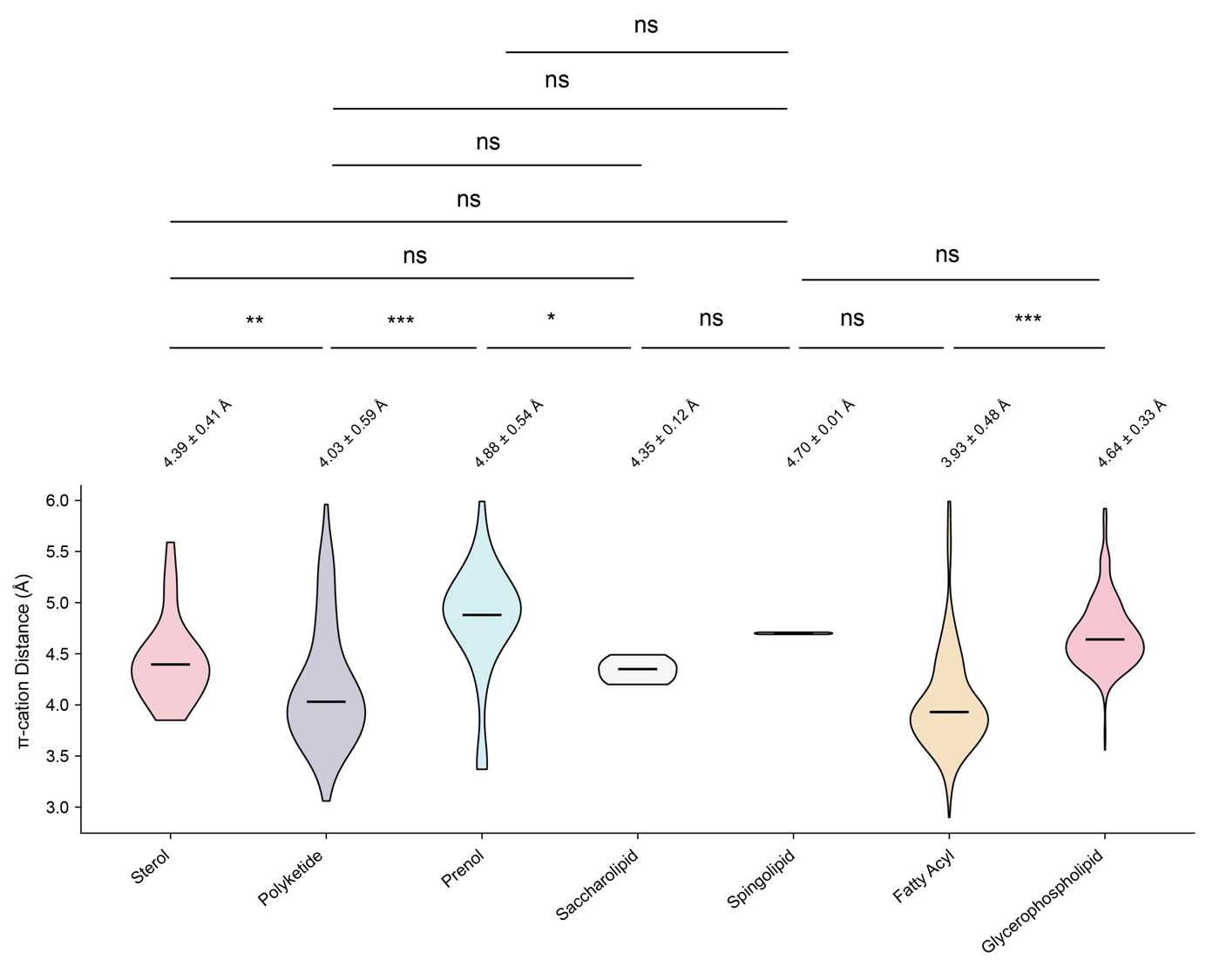


**Figure S16.** **Distance for π-cation interactions across lipid-protein complexes in BioDolphin.** Distance for π-cation interactions (“DIST”) between lipid and protein atoms in lipid-protein complexes across the eight classes of lipids. π-cation interactions and their distance were obtained from a PLIP analysis of all 113,782 entries in the BioDolphin database. The median ± standard deviation π-cation distances is shown for each class of lipid. Note that PLIP did not identify any π-cation interactions for glycerolipid-protein complexes. Pairwise differences between π-cation interaction distances between lipid classes were assessed using a two-sided Mann–Whitney U test. *p*-values were adjusted for multiple comparisons using the Benjamini–Hochberg procedure (FDR < 0.05) as implemented in statsmodels. ns = not significant. * = *p* < 0.05 ; ** = *p* < 0.01 ; *** = *p* < 0.001. Statistical significance (*, **, or ***) is denoted only for select datasets for clarity. All not significant datasets are denoted.


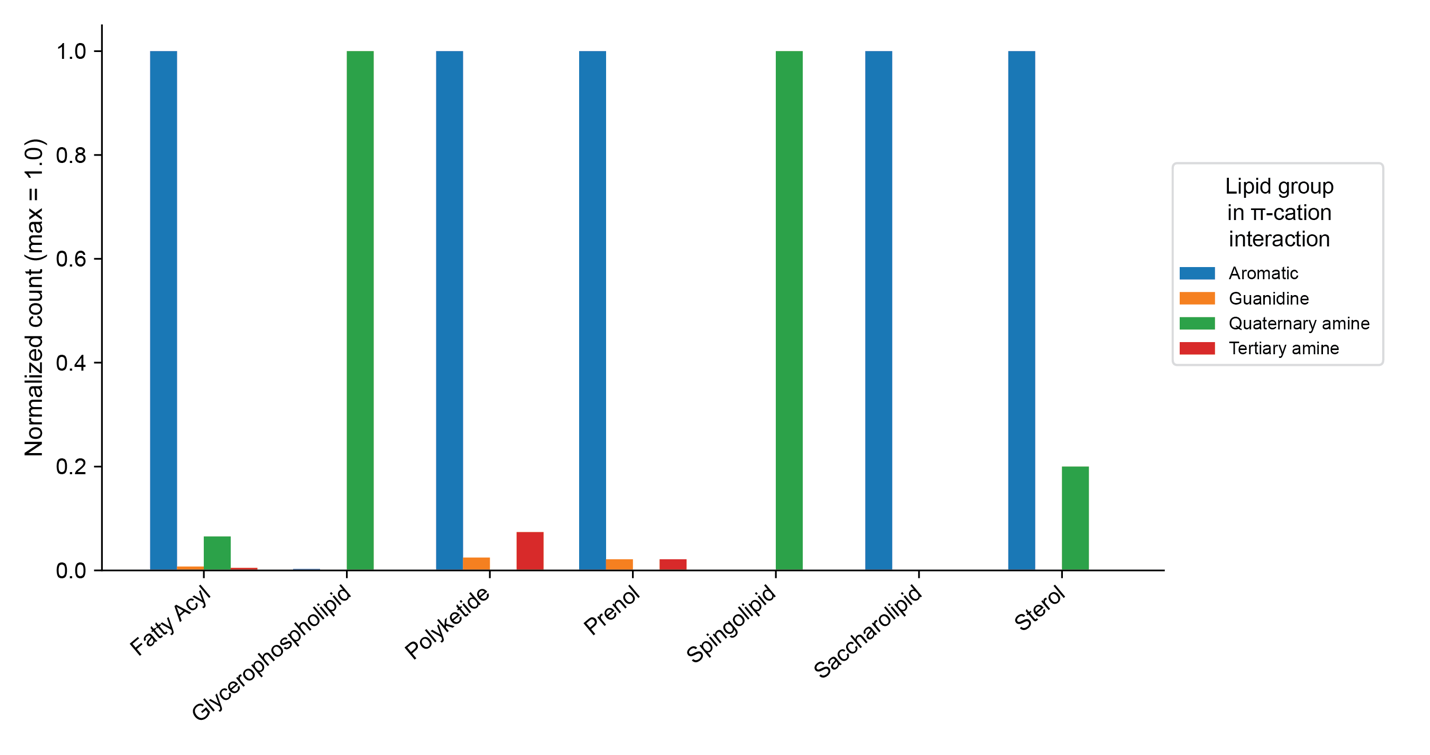


**Figure S17.** **Groups of lipids involved in π-cation interactions across lipid-protein complexes in BioDolphin.** Lipid chemical group involved in π-cation interactions (“LIG_GROUP”) in lipid-protein complexes across the eight classes of lipids. Lipid groups involved in π-cation interactions were obtained and normalized (relative to the highest count in each lipid class) from a PLIP analysis of all 113,782 entries in the BioDolphin database. Note that PLIP did not identify any π-cation interactions for glycerolipid-protein complexes.


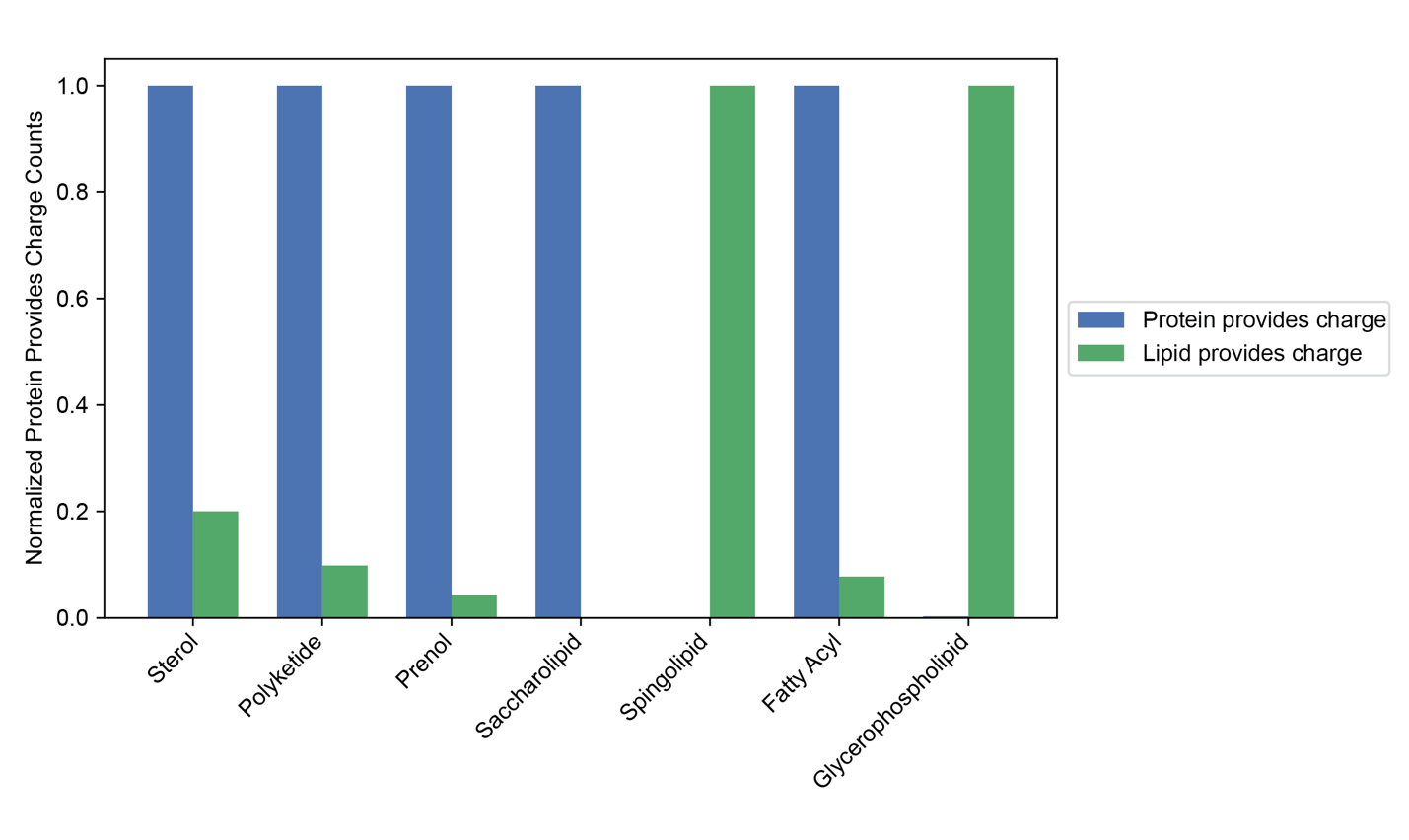


**Figure S18.** **Information on what molecule provides the charge in π-cation interactions across lipid-protein complexes in BioDolphin.** Classification of whether the protein or the lipid provides the charge in π-cation interactions (“PROTCHARGED”) in lipid-protein complexes across the eight classes of lipids. Charge information in π-cation interactions was obtained and normalized (relative to the highest count in each lipid class) from a PLIP analysis of all 113,782 entries in the BioDolphin database. Note that PLIP did not identify any π-cation interactions for glycerolipid-protein complexes.


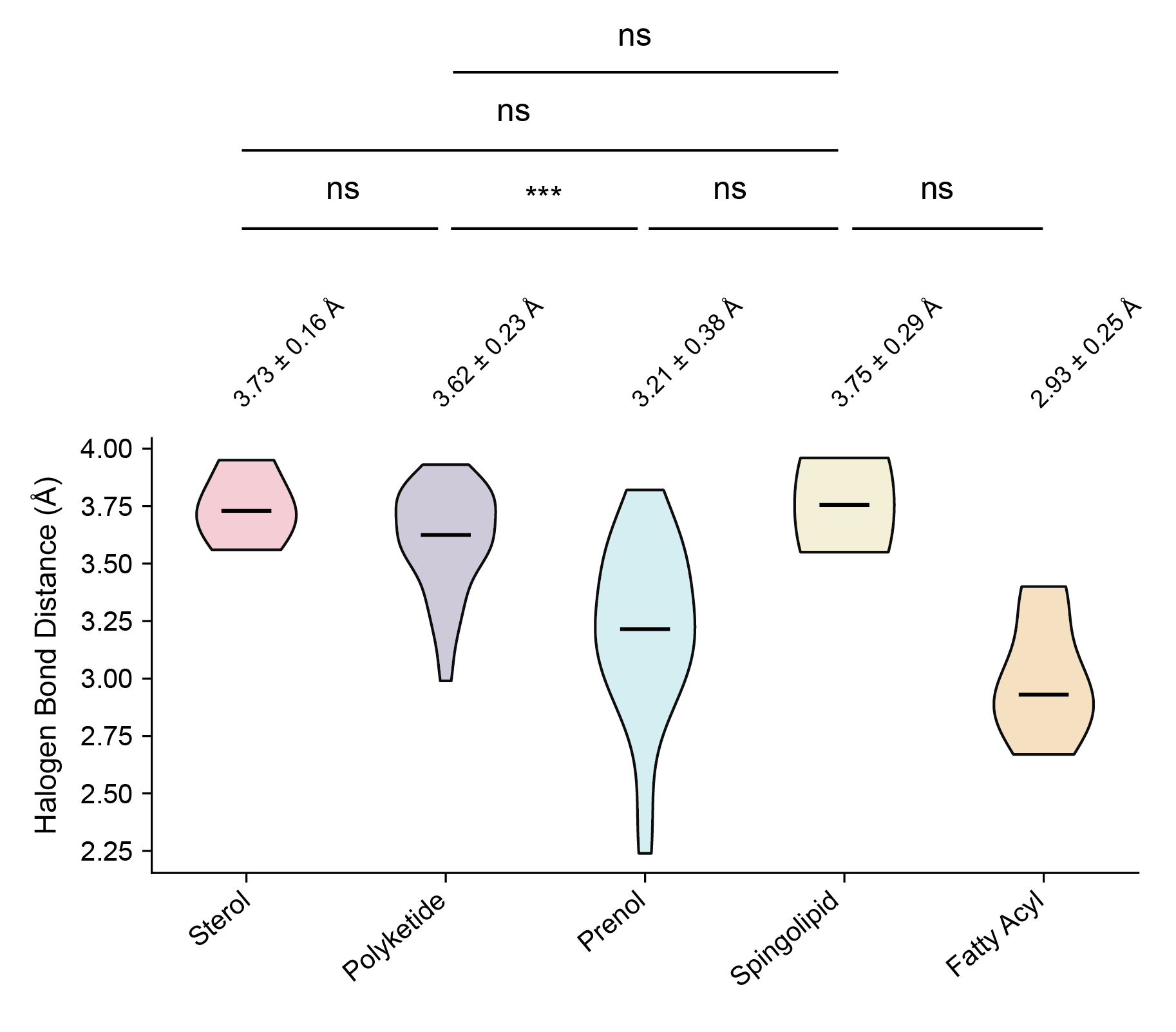


**Figure S19.** **Halogen bond distance across lipid-protein complexes in BioDolphin.** Distances between halogen donor and acceptor atoms (“DIST”) in lipid-protein complexes across the eight classes of lipids. Distances were obtained from a PLIP analysis of all 113,782 entries in the BioDolphin database. The median ± standard deviation halogen bond distance is shown for each class of lipid. Note that PLIP did not identify any halogen bond interactions for saccharolipid-protein, glycerolipid-protein, or glycerophospholipid-protein complexes. Pairwise differences between halogen bond interaction distances between lipid classes were assessed using a two-sided Mann–Whitney U test. *p*-values were adjusted for multiple comparisons using the Benjamini–Hochberg procedure (FDR < 0.05) as implemented in statsmodels. ns = not significant. * = *p* < 0.05 ; ** = *p* < 0.01 ; *** = *p* < 0.001. Statistical significance (*, **, or ***) is denoted only for select datasets for clarity. All not significant datasets are denoted.


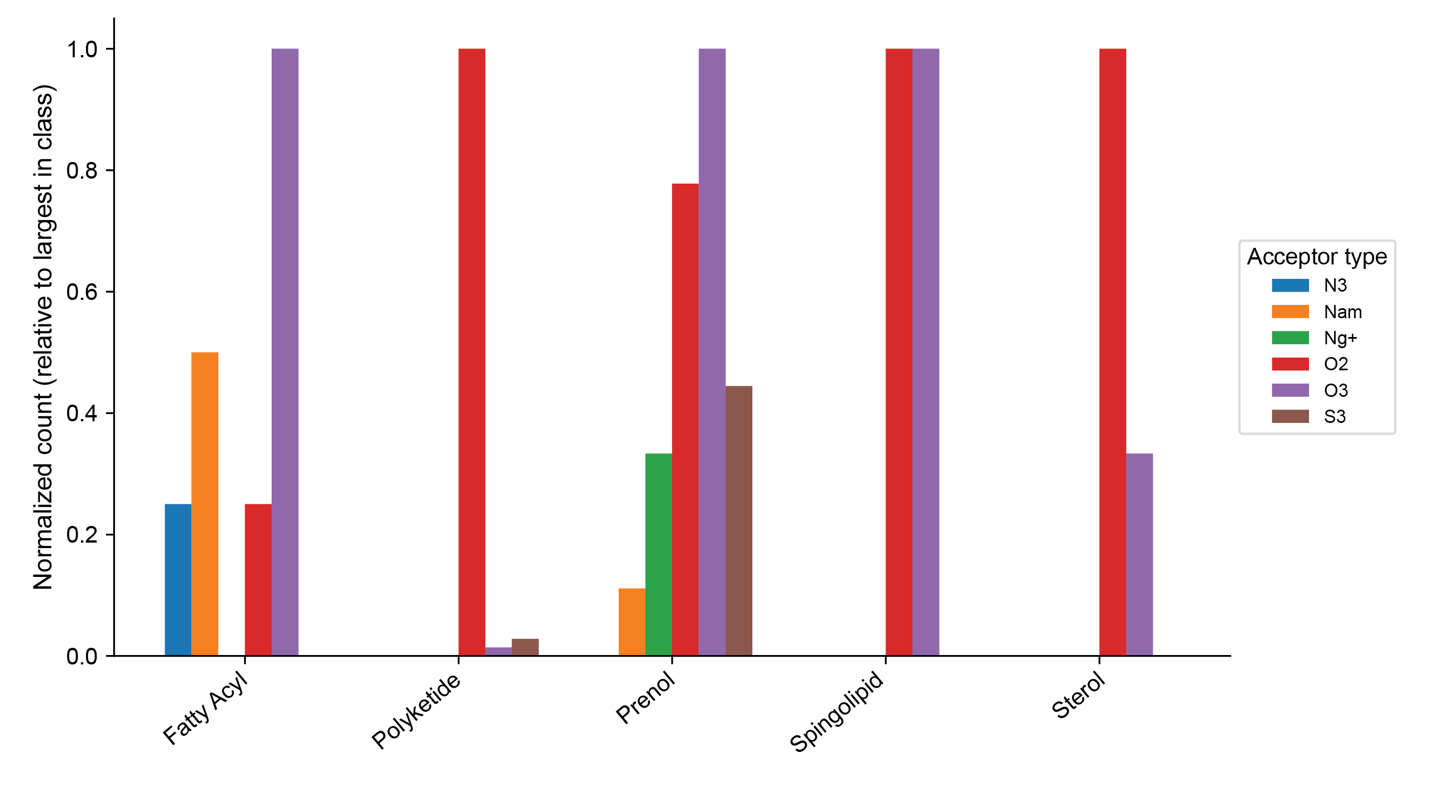


**Figure S20.** **Acceptor atom types in halogen bonds across lipid-protein complexes in BioDolphin.** Acceptor atom types in halogen bonds (“ACCEPTORTYPE”) in lipid-protein complexes across the eight classes of lipids. Acceptor atom types were obtained and normalized (relative to the highest count in each lipid class) from a PLIP analysis of all 113,782 entries in the BioDolphin database. The acceptor atom type names correspond to Open Babel's atom typing system that uses SMARTS patterns in a configuration file (atomtyp.txt) as follows: N3 - sp3 hybridized nitrogen (tetrahedral, i.e., amines); Nam - Nitrogen in amide group (i.e., N-C=O); Nar - aromatic nitrogen; Ng+ - positively charged nitrogen in generic conjugated system; O2 - sp2 hybridized oxygen (i.e., carbonyl); O3 - sp3 hybridized oxygen (i.e., hydroxyl); S3 - sp3 hybridized sulfur (i.e., thiols). Note that PLIP did not identify any halogen bond interactions for saccharolipid-protein, glycerolipid-protein, or glycerophospholipid-protein complexes.


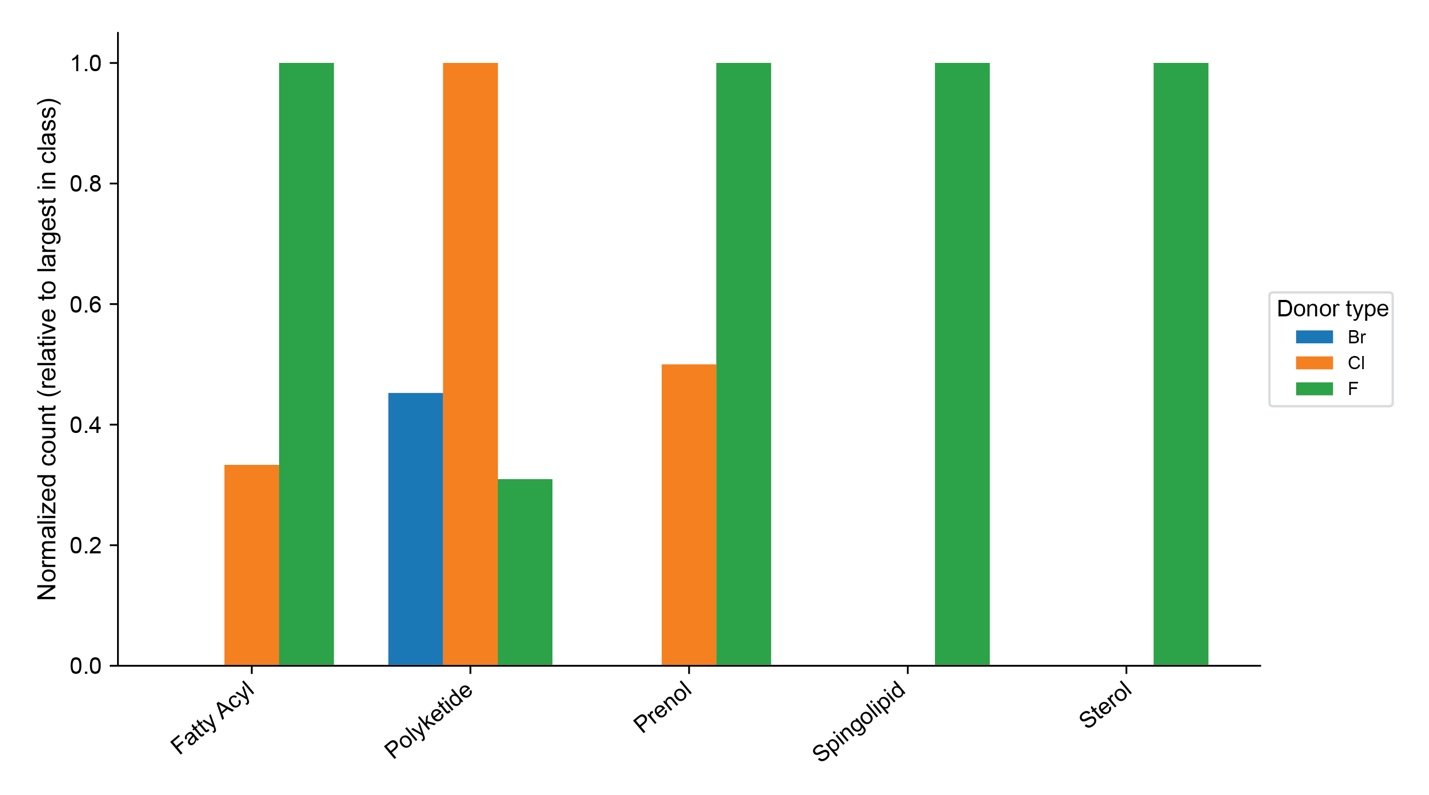


**Figure S21.** **Donor atom types in halogen bonds across lipid-protein complexes in BioDolphin.** Donor atom types in halogen bonds (“DONORTYPE”) in lipid-protein complexes across the eight classes of lipids. Donor atom types were obtained and normalized (relative to the highest count in each lipid class) from a PLIP analysis of all 113,782 entries in the BioDolphin database. Note that PLIP did not identify any halogen bond interactions for saccharolipid-protein, glycerolipid-protein, or glycerophospholipid-protein complexes.


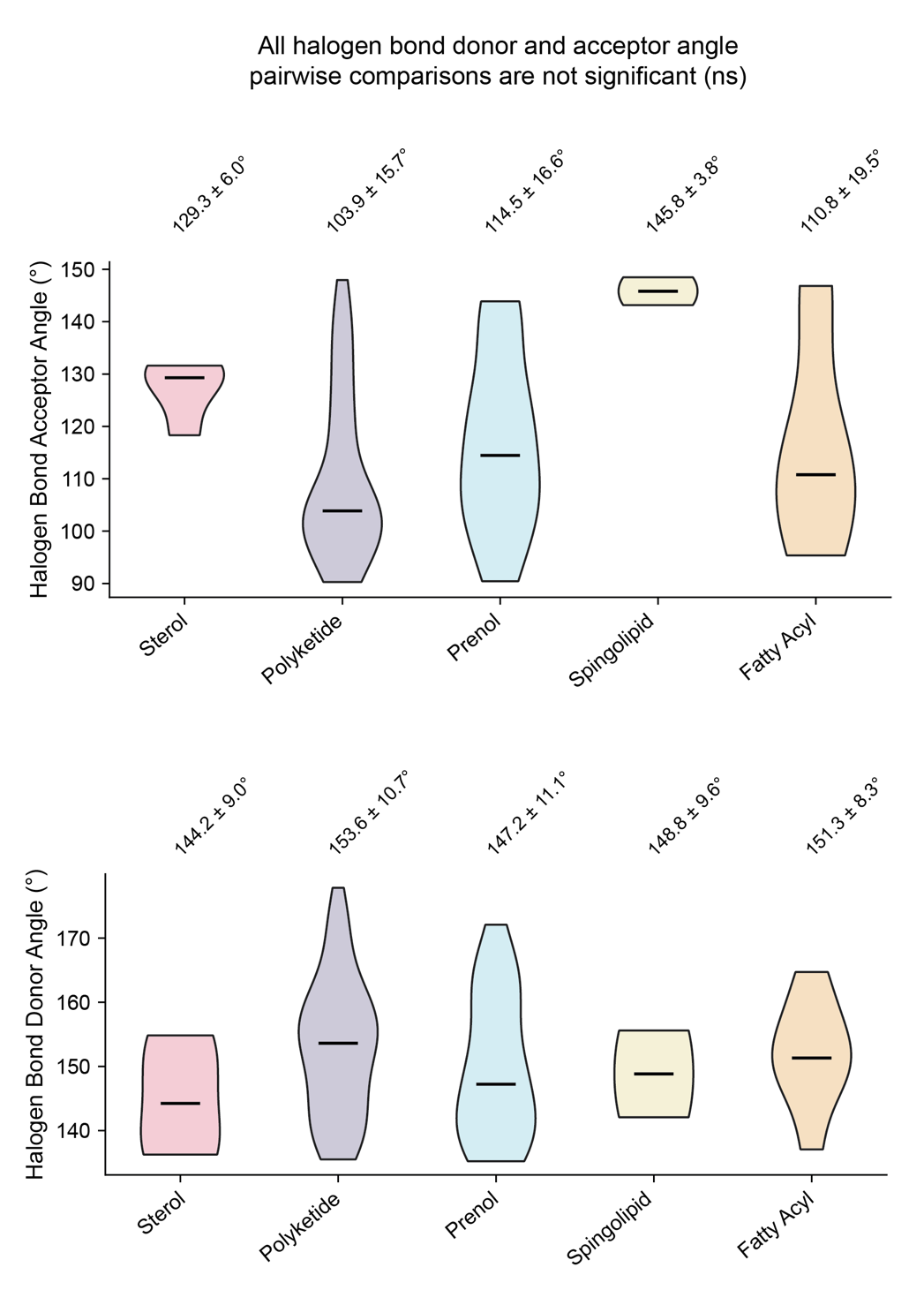


**Figure S22.** **Donor and acceptor angles in halogen bonds across lipid-protein complexes in BioDolphin.** Angle at the donor atom and acceptor atom of halogen bonds (“DON_ANGLE” and “ACC_ANGLE”) in lipid-protein complexes across the eight classes of lipids. Angles were obtained from a PLIP analysis of all 113,782 entries in the BioDolphin database. The median ± standard deviation halogen bond angle is shown for each class of lipid. Note that PLIP did not identify any halogen bond interactions for saccharolipid-protein, glycerolipid-protein, or glycerophospholipid-protein complexes. Pairwise differences between halogen bond donor and accept angles between lipid classes were assessed using a two-sided Mann–Whitney U test. *p*-values were adjusted for multiple comparisons using the Benjamini–Hochberg procedure (FDR < 0.05) as implemented in statsmodels. ns = not significant. * = *p* < 0.05 ; ** = *p* < 0.01 ; *** = *p* < 0.001. In case, all comparisons are considered not significant.


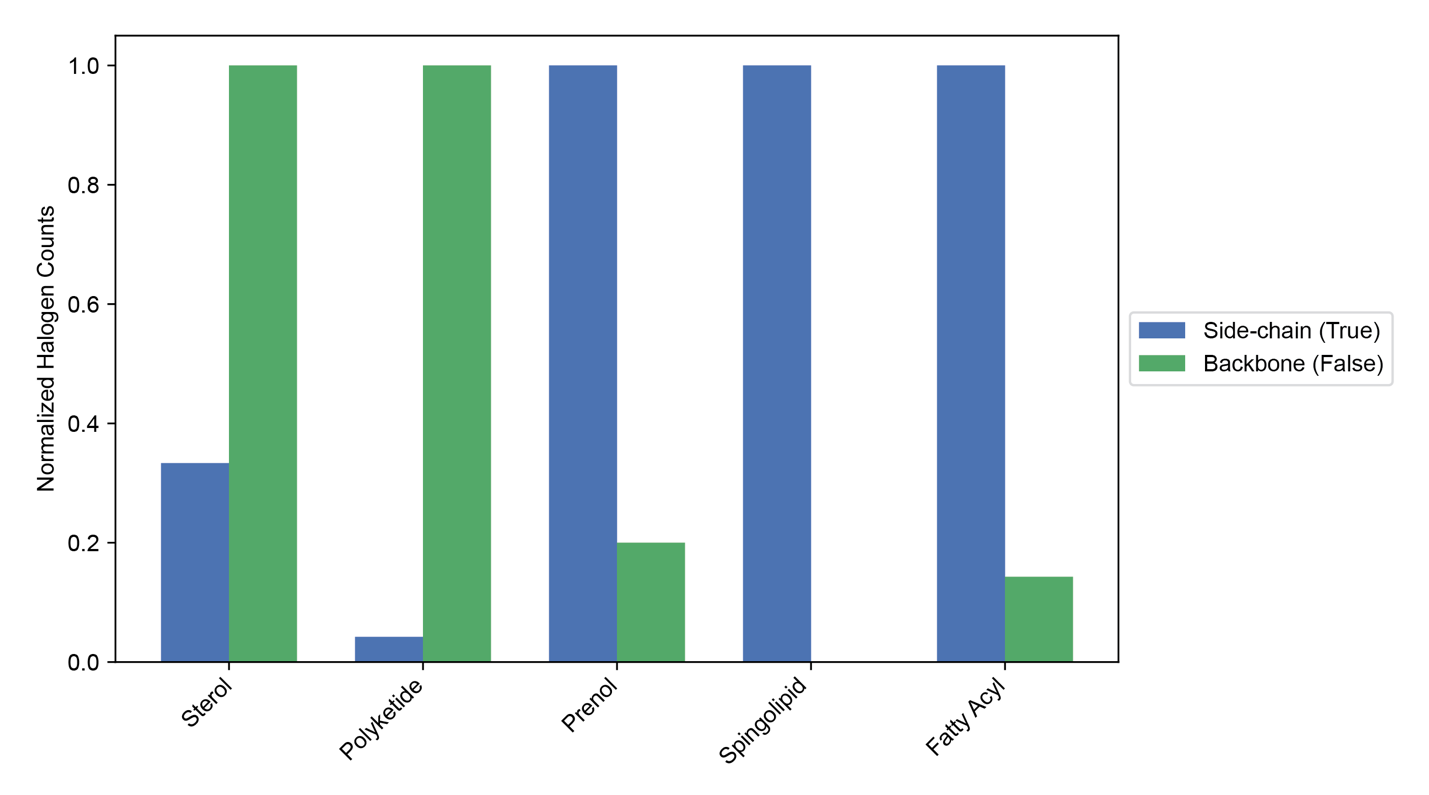


**Figure S23.** **Characterizing halogen bonds of lipid atoms to side-chain vs backbone atoms of proteins across lipid-protein complexes in BioDolphin.** Characterizing the location of halogen bonds (side-chain or not) between lipid atoms and protein atoms (“SIDECHAIN”) in lipid-protein complexes across the eight classes of lipids. The identity of the halogen bond (side-chain or backbone atoms) from a PLIP analysis of all 113,782 entries in the BioDolphin database. Counts were normalized relative to the highest count in each lipid class. Note that PLIP did not identify any halogen bond interactions for saccharolipid-protein, glycerolipid-protein, or glycerophospholipid-protein complexes.

**
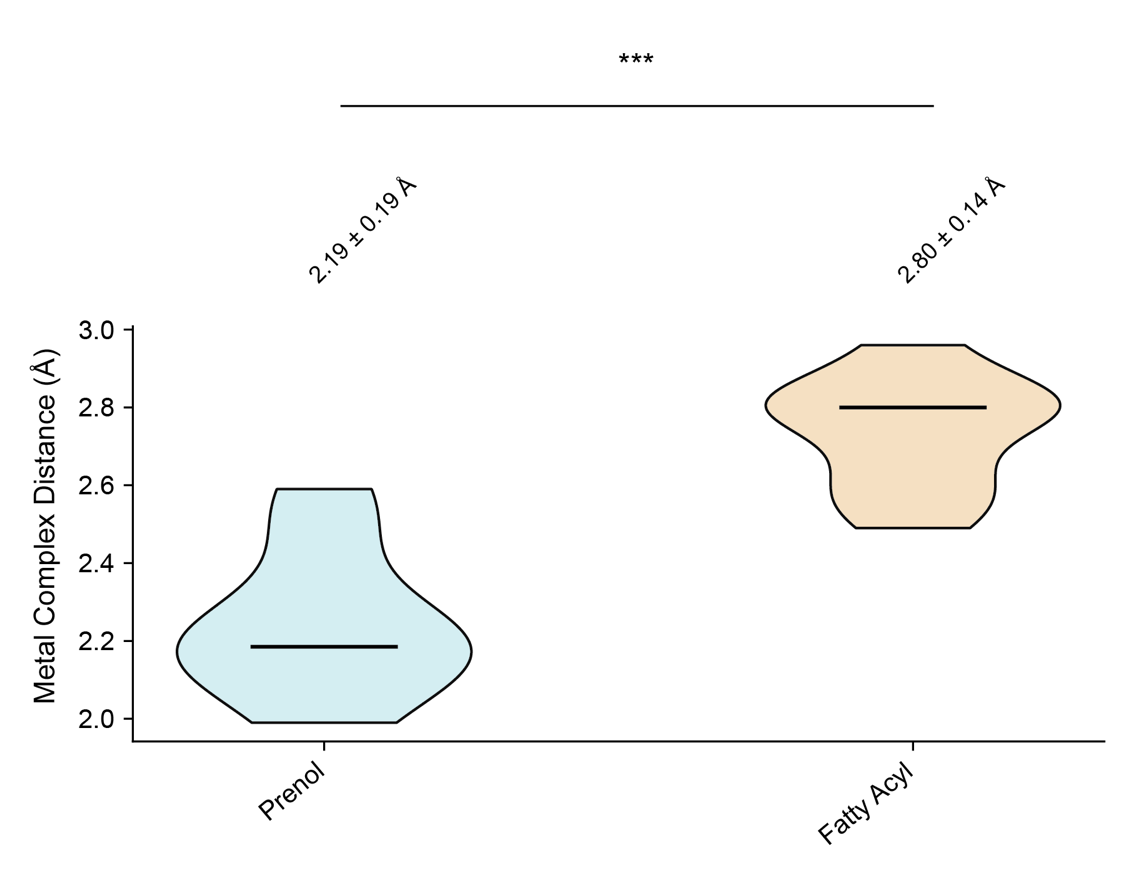
**

**Figure S24.** **Metal-coordinated interaction distance across lipid-protein complexes in BioDolphin.** Distances between metal atoms in lipid and protein atoms (“DIST”) in lipid-protein complexes across the eight classes of lipids. Distances were obtained from a PLIP analysis of all 113,782 entries in the BioDolphin database. The median ± standard deviation metal-coordinated interaction distance is shown for each class of lipid. Note that PLIP did not identify any metal-coordinated interactions for sterol-protein, polyketide-protein, saccharolipid-protein, sphingolipid-protein, glycerolipid-protein, or glycerophospholipid-protein complexes. Pairwise differences between metal complex distances between lipid classes were assessed using a two-sided Mann–Whitney U test. *p*-values were adjusted for multiple comparisons using the Benjamini–Hochberg procedure (FDR < 0.05) as implemented in statsmodels. ns = not significant. * = *p* < 0.05 ; ** = *p* < 0.01 ; *** = *p* < 0.001. Statistical significance (*, **, or ***) is denoted only for select datasets for clarity. All not significant datasets are denoted.


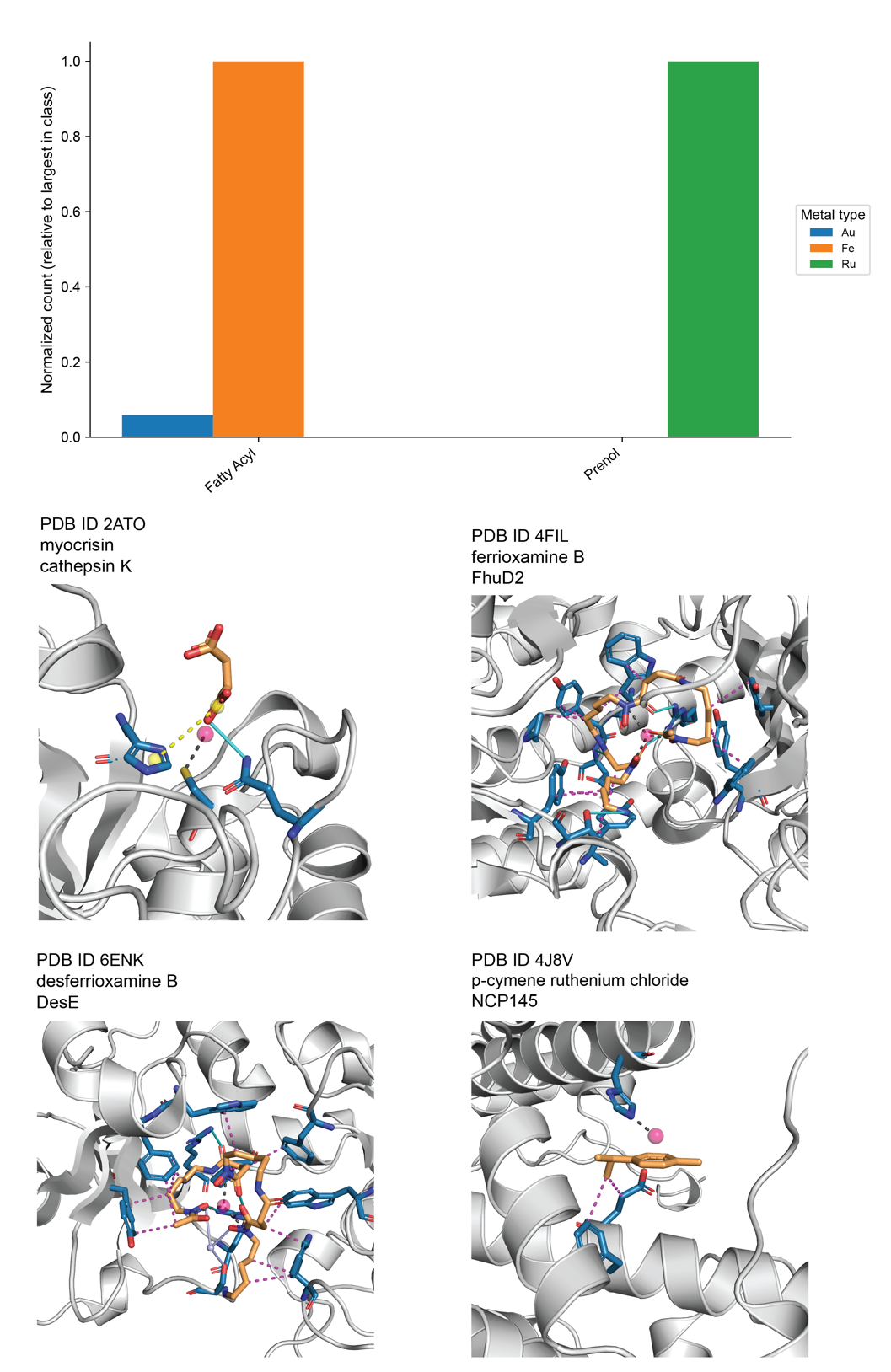


**Figure S25.** **Metal types in metal-coordinated interactions across lipid-protein complexes in BioDolphin.** Top: Metal types in lipids involved in meta-coordinated interactions (“METAL_TYPE”) in lipid-protein complexes across the eight classes of lipids. Metal types were obtained from a PLIP analysis of all 113,782 entries in the BioDolphin database. Counts were normalized relative to the highest count in each lipid class. Note that PLIP did not identify any metal-coordinated interactions for sterol-protein, polyketide-protein, saccharolipid-protein, sphingolipid-protein, glycerolipid-protein, or glycerophospholipid-protein complexes. Bottom: Example of PLIP analysis highlighting metal complex interactions with protein atoms shown in grey cartoon/blue sticks, lipid atoms shown in orange, and metal atoms as spheres.


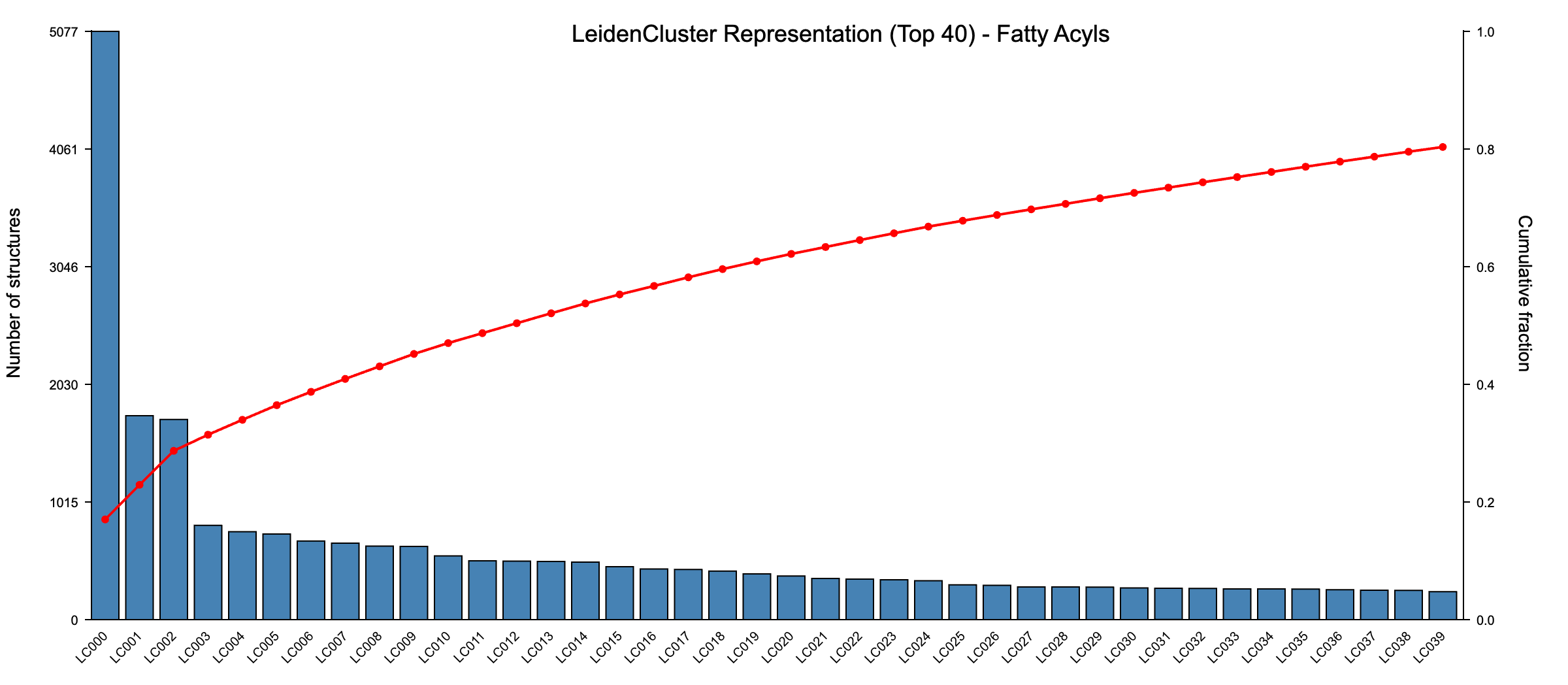


**Figure S26.** **Leiden cluster representation and structural redundancy.** Leiden clusters were derived from Protein Cartography and used as proxies for protein families and structural folds. The bar plot shows the number of structures in the top 40 most populated clusters for the specific lipid class. The red line indicates the cumulative fraction of all structures accounted for by these clusters, relative to the full dataset for each lipid class. A dataset is considered highly redundant when a small number of highly populated clusters account for a substantial fraction (>80%) of all entries, indicating strong overrepresentation of a limited number of protein families or folds.


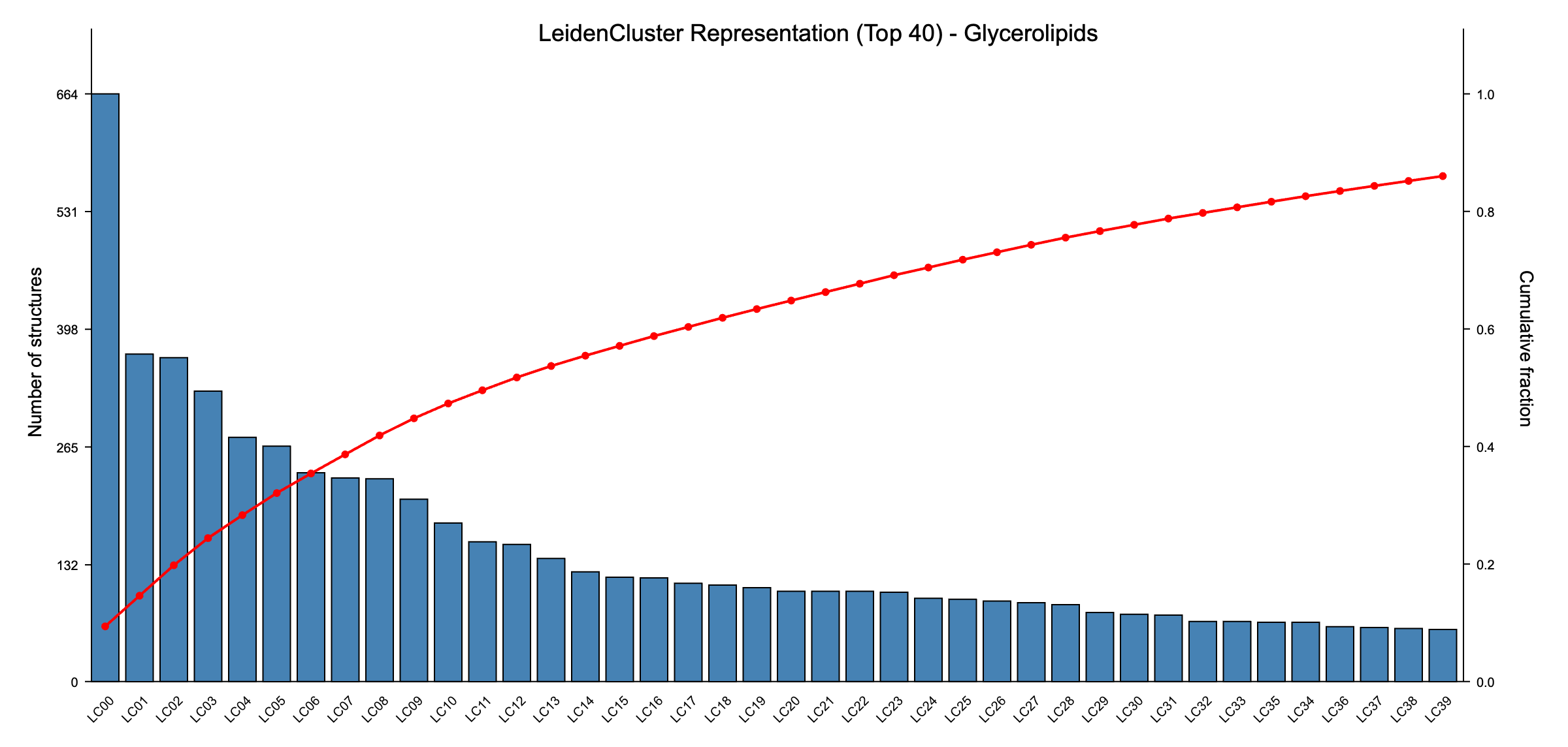


**Figure S26 continued.** **Leiden cluster representation and structural redundancy.** Leiden clusters were derived from Protein Cartography and used as proxies for protein families and structural folds. The bar plot shows the number of structures in the top 40 most populated clusters for the specific lipid class. The red line indicates the cumulative fraction of all structures accounted for by these clusters, relative to the full dataset for each lipid class. A dataset is considered highly redundant when a small number of highly populated clusters account for a substantial fraction (>80%) of all entries, indicating strong overrepresentation of a limited number of protein families or folds.


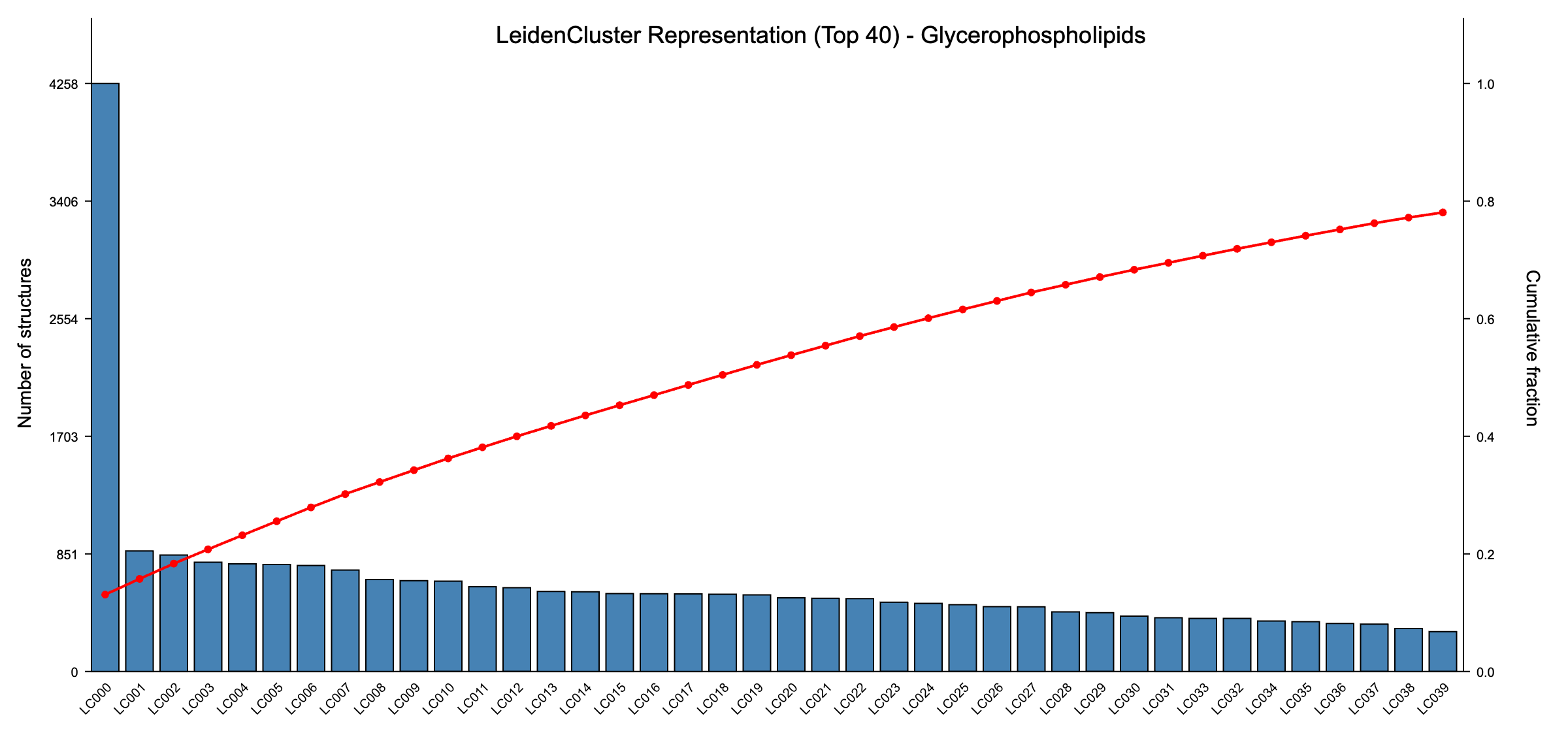


**Figure S26 continued.** **Leiden cluster representation and structural redundancy.** Leiden clusters were derived from Protein Cartography and used as proxies for protein families and structural folds. The bar plot shows the number of structures in the top 40 most populated clusters for the specific lipid class. The red line indicates the cumulative fraction of all structures accounted for by these clusters, relative to the full dataset for each lipid class. A dataset is considered highly redundant when a small number of highly populated clusters account for a substantial fraction (>80%) of all entries, indicating strong overrepresentation of a limited number of protein families or folds.


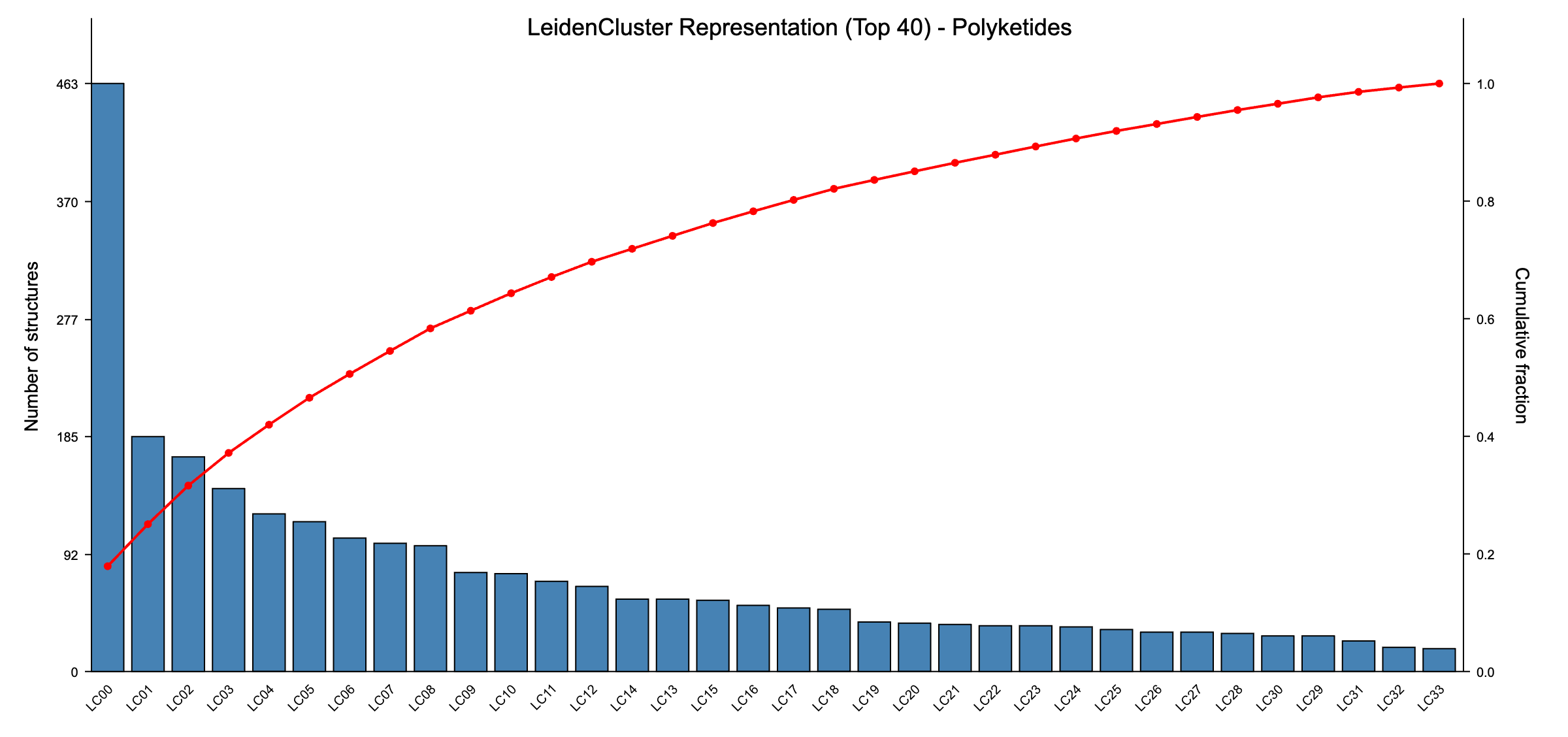


**Figure S26 continued.** **Leiden cluster representation and structural redundancy.** Leiden clusters were derived from Protein Cartography and used as proxies for protein families and structural folds. The bar plot shows the number of structures in the top 40 most populated clusters for the specific lipid class. The red line indicates the cumulative fraction of all structures accounted for by these clusters, relative to the full dataset for each lipid class. A dataset is considered highly redundant when a small number of highly populated clusters account for a substantial fraction (>80%) of all entries, indicating strong overrepresentation of a limited number of protein families or folds.


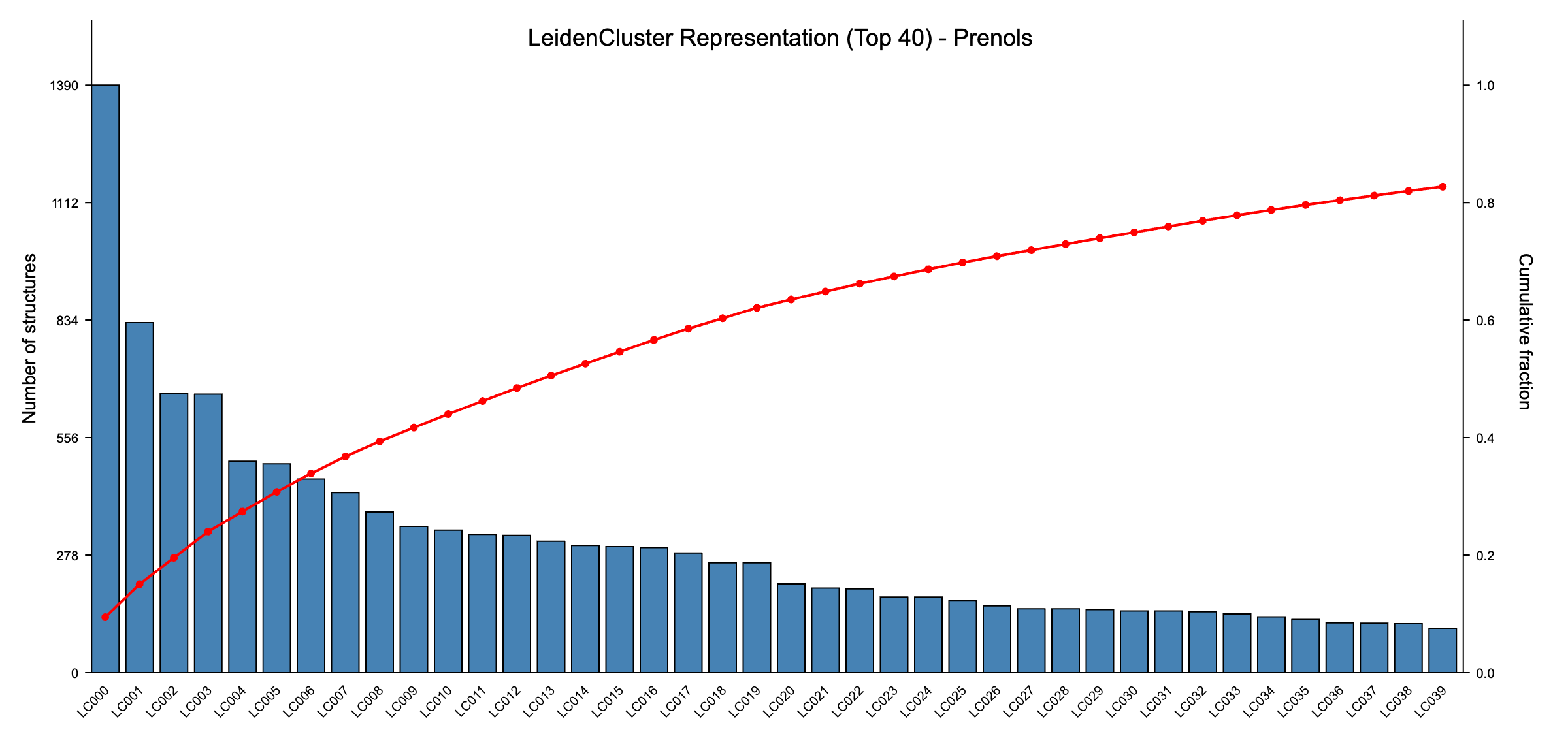


**Figure S26 continued.** **Leiden cluster representation and structural redundancy.** Leiden clusters were derived from Protein Cartography and used as proxies for protein families and structural folds. The bar plot shows the number of structures in the top 40 most populated clusters for the specific lipid class. The red line indicates the cumulative fraction of all structures accounted for by these clusters, relative to the full dataset for each lipid class. A dataset is considered highly redundant when a small number of highly populated clusters account for a substantial fraction (>80%) of all entries, indicating strong overrepresentation of a limited number of protein families or folds.


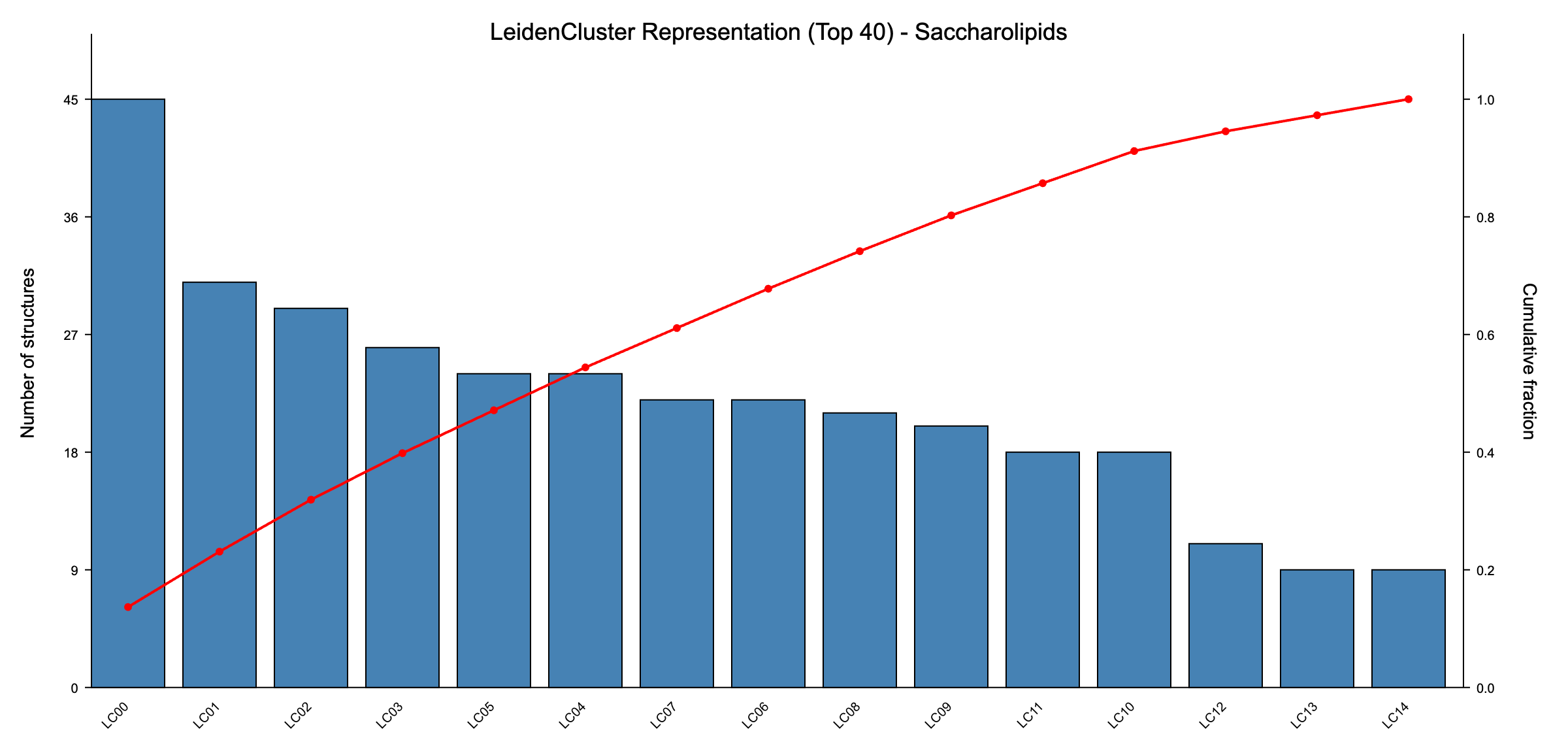


**Figure S26 continued.** **Leiden cluster representation and structural redundancy.** Leiden clusters were derived from Protein Cartography and used as proxies for protein families and structural folds. The bar plot shows the number of structures in the top 40 most populated clusters for the specific lipid class. The red line indicates the cumulative fraction of all structures accounted for by these clusters, relative to the full dataset for each lipid class. A dataset is considered highly redundant when a small number of highly populated clusters account for a substantial fraction (>80%) of all entries, indicating strong overrepresentation of a limited number of protein families or folds.


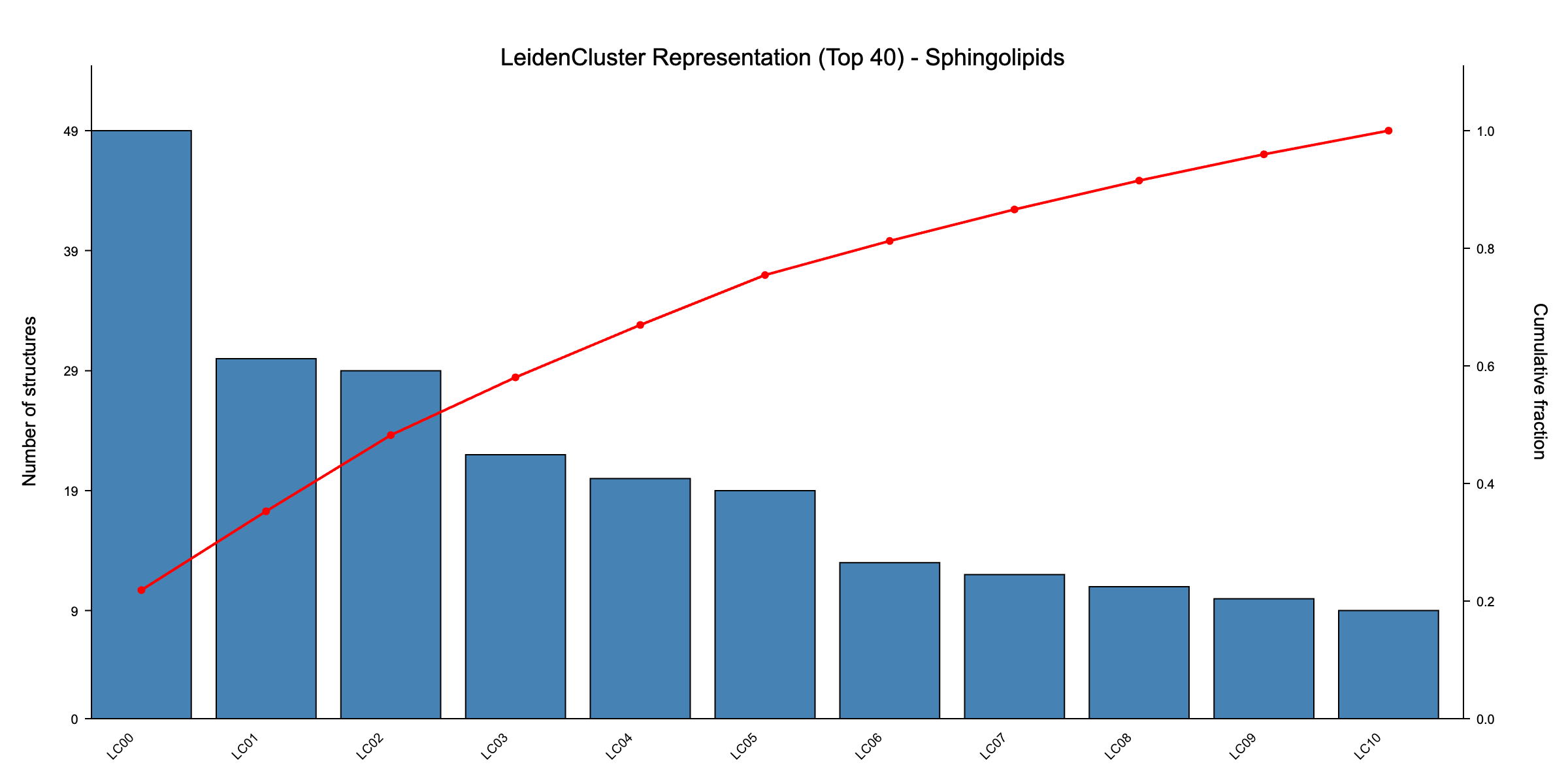


**Figure S26 continued.** **Leiden cluster representation and structural redundancy.** Leiden clusters were derived from Protein Cartography and used as proxies for protein families and structural folds. The bar plot shows the number of structures in the top 40 most populated clusters for the specific lipid class. The red line indicates the cumulative fraction of all structures accounted for by these clusters, relative to the full dataset for each lipid class. A dataset is considered highly redundant when a small number of highly populated clusters account for a substantial fraction (>80%) of all entries, indicating strong overrepresentation of a limited number of protein families or folds.


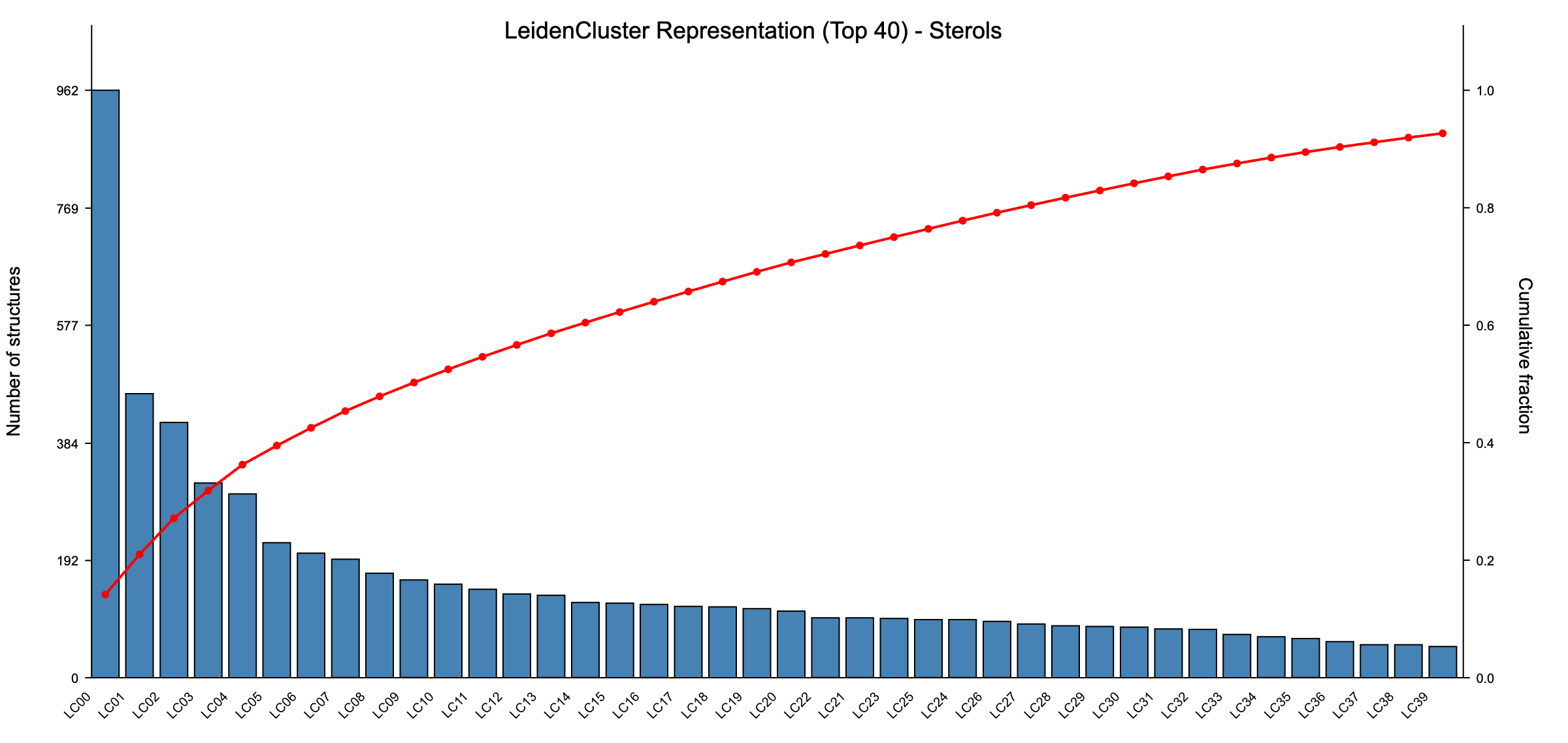


**Figure S26 continued.** **Leiden cluster representation and structural redundancy.** Leiden clusters were derived from Protein Cartography and used as proxies for protein families and structural folds. The bar plot shows the number of structures in the top 40 most populated clusters for the specific lipid class. The red line indicates the cumulative fraction of all structures accounted for by these clusters, relative to the full dataset for each lipid class. A dataset is considered highly redundant when a small number of highly populated clusters account for a substantial fraction (>80%) of all entries, indicating strong overrepresentation of a limited number of protein families or folds.


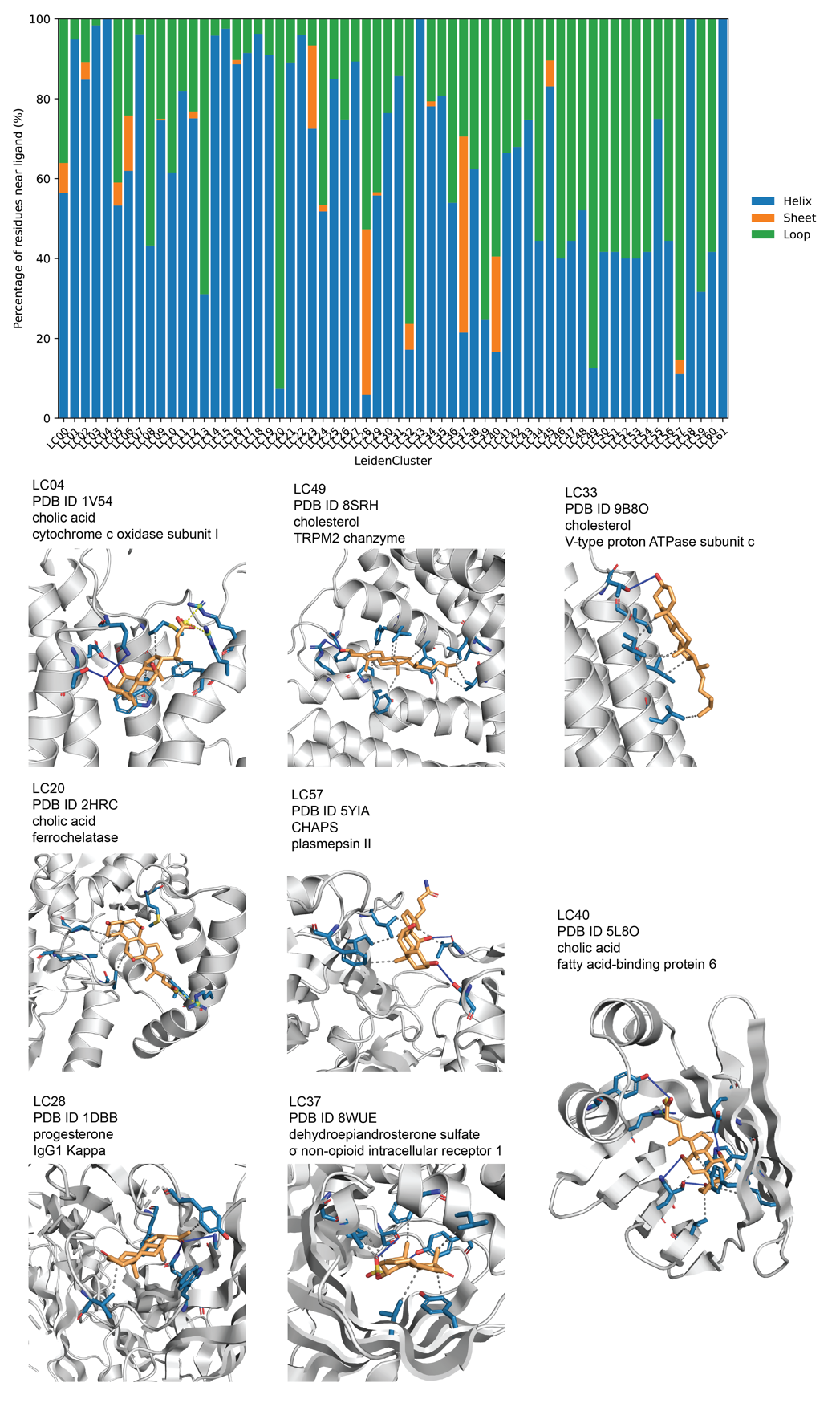


**Figure S27. Landscape of secondary structure content of sterol binding protein proteins across different Leiden clusters.** Top: comparison of secondary structure content of protein residues within 5 Å of the sterol ligand across different Leiden clusters derived using the ProteinCartography tool. Bottom: Representative sterol binding protein structures: protein backbone shown as grey cartoon, PLIP-determine interacting residues shown as blue sticks, and the lipid shown in orange sticks.


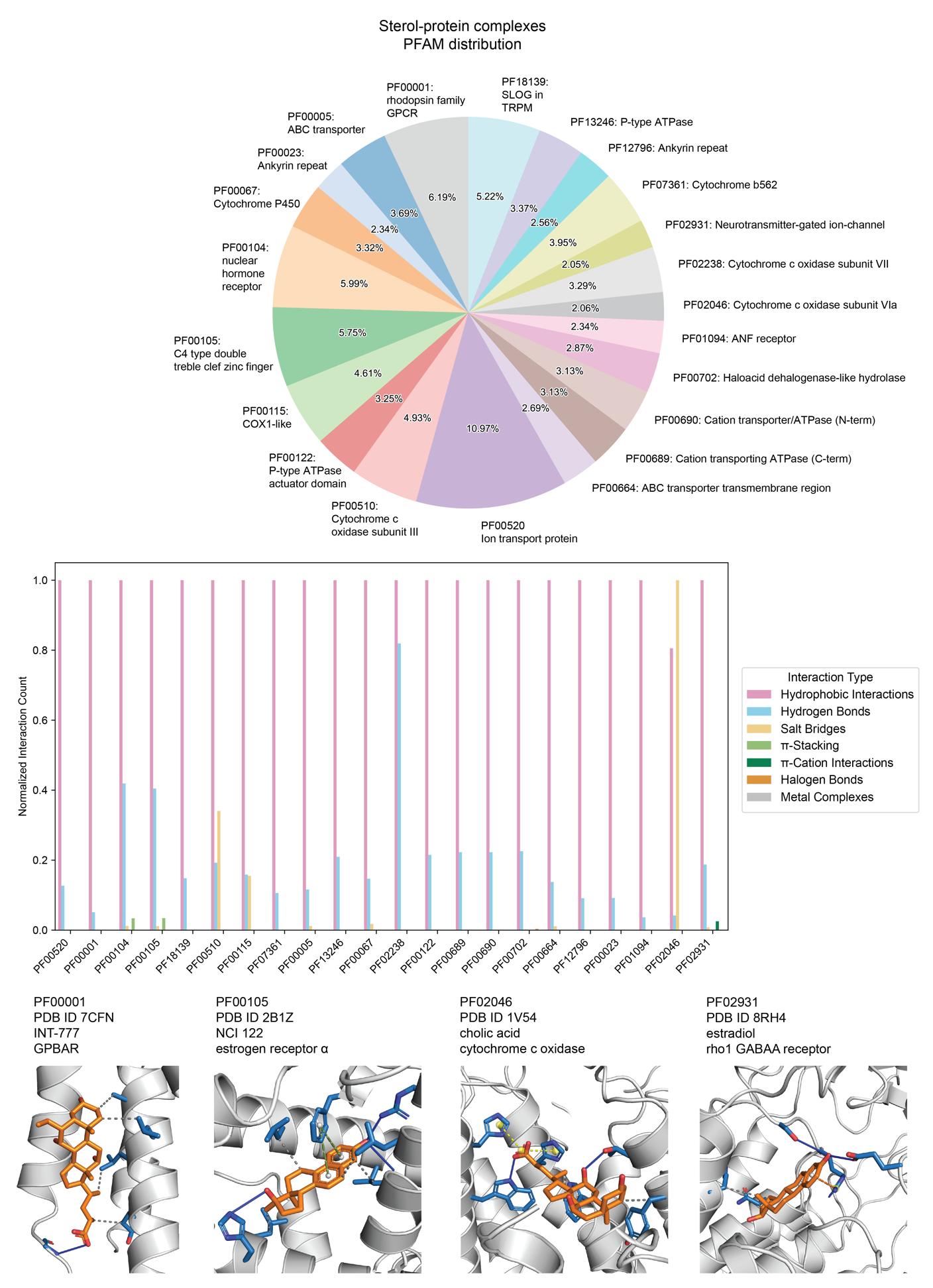


**Figure S28.** **PFAMs and interaction profiles for sterol-protein complexes.** Top: Pie chart of PFAMs associated with the most common sterol-protein complexes. The PFAM frequency values represent the number of PFAM occurrences across sterol-protein complexes in the BioDolphin database. Middle: Normalized number of interactions performed for each PFAM separately. Interaction counts were normalized by dividing counts for each interaction type by the highest count within each PFAM. Bottom: Representative examples PLIP-determined interactions in sterol-protein complexes. Protein backbone shown as grey cartoon, PLIP-determine interacting residues shown as blue sticks, and the lipid shown in orange sticks.


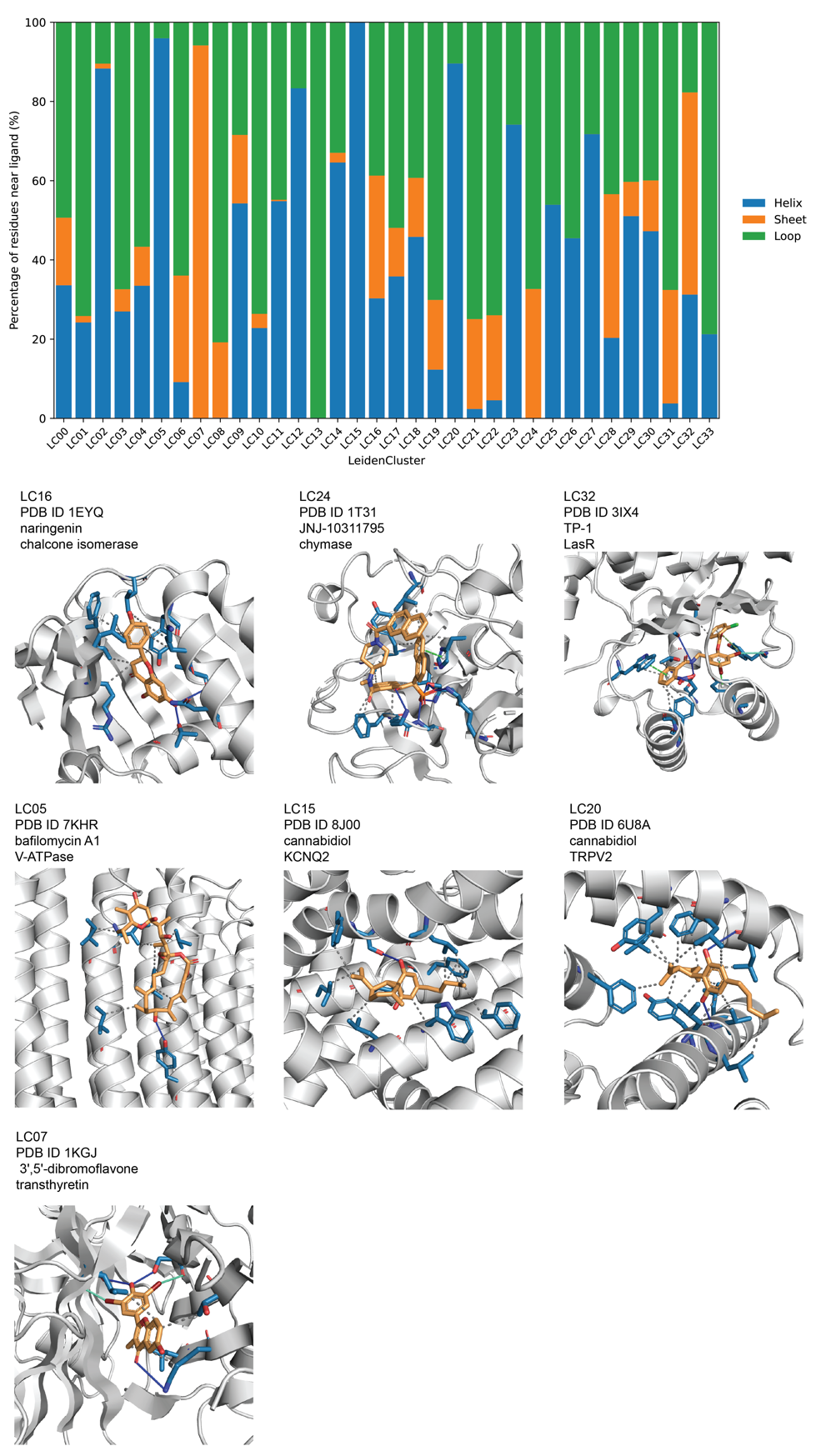


**Figure S29. Landscape of secondary structure content of polyketide binding protein proteins across different Leiden clusters.** Top: comparison of secondary structure content of protein residues within 5 Å of the polyketide ligand across different Leiden clusters derived using the ProteinCartography tool. Bottom: Representative polyketide binding protein structures: protein backbone shown as grey cartoon, PLIP-determine interacting residues shown as blue sticks, and the lipid shown in orange sticks.


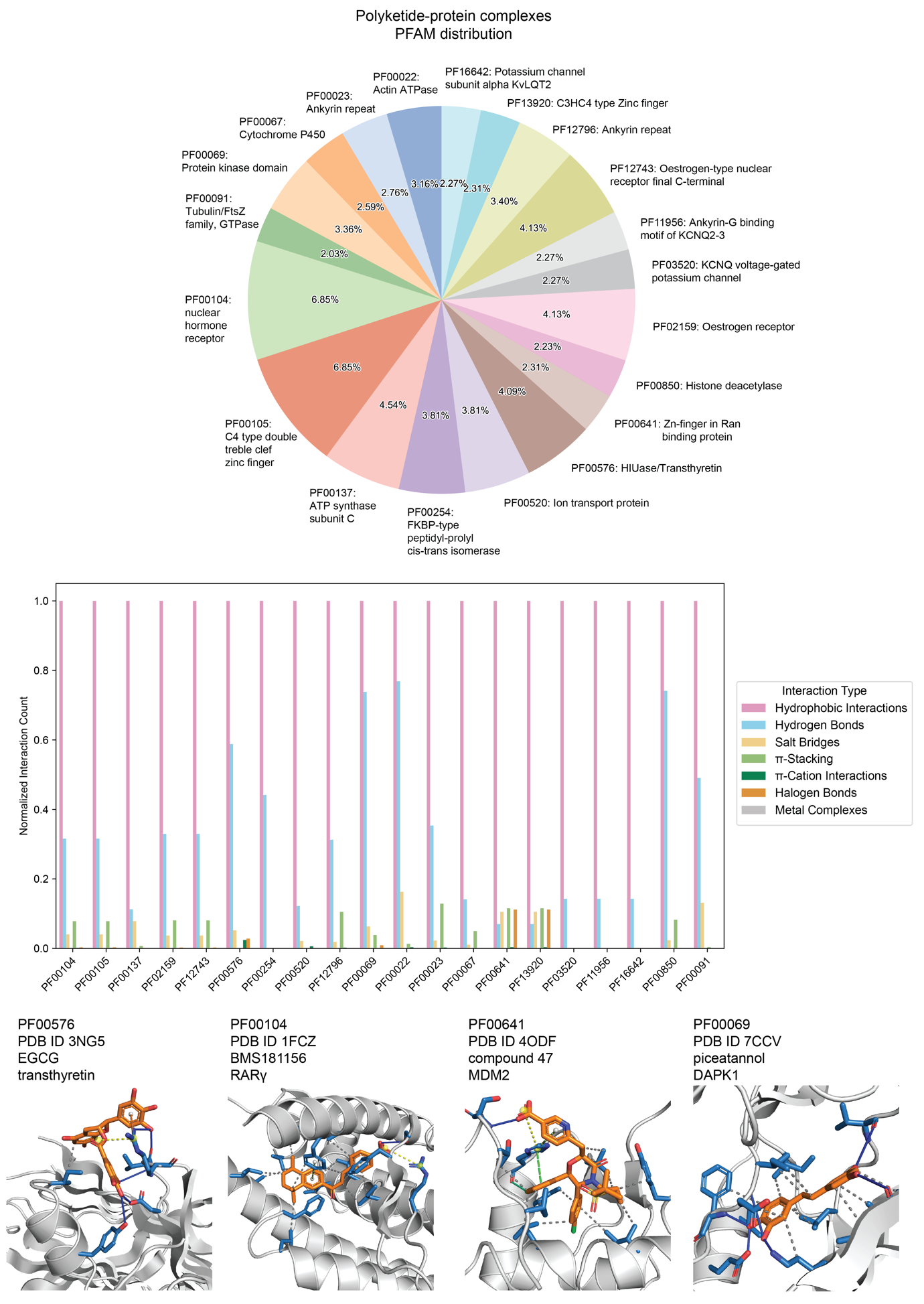


**Figure S30.** **PFAMs and interaction profiles for polyketide-protein complexes.** Top: Pie chart of PFAMs associated with the most common polyketide-protein complexes. The PFAM frequency values represent the number of PFAM occurrences across polyketide-protein complexes in the BioDolphin database. Middle: Normalized number of interactions performed for each PFAM separately. Interaction counts were normalized by dividing counts for each interaction type by the highest count within each PFAM. Bottom: Representative examples PLIP-determined interactions in polyketide-protein complexes. Protein backbone shown as grey cartoon, PLIP-determine interacting residues shown as blue sticks, and the lipid shown in orange sticks.


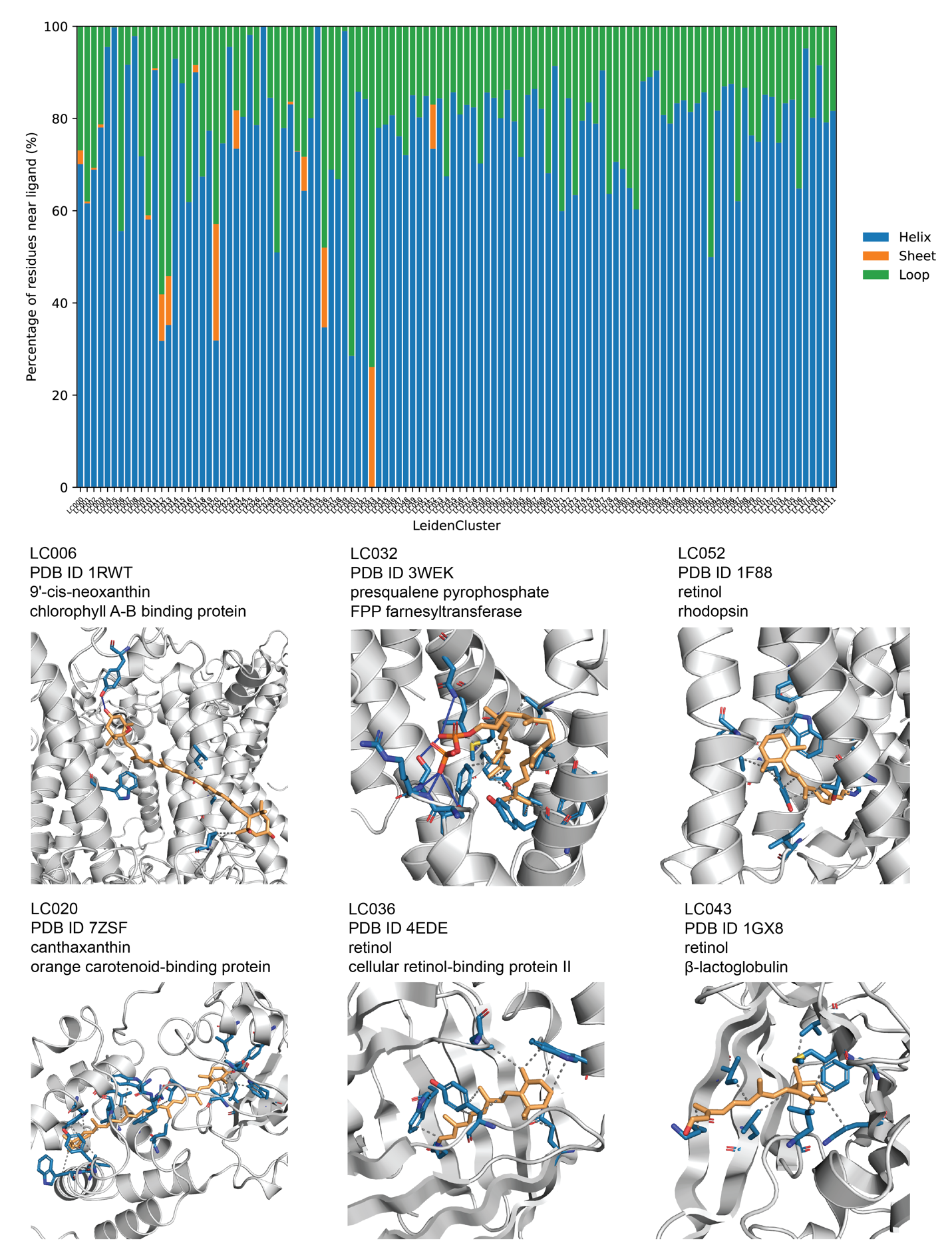


**Figure S31. Landscape of secondary structure content of prenol binding protein proteins across different Leiden clusters.** Top: comparison of secondary structure content of protein residues within 5 Å of the prenol ligand across different Leiden clusters derived using the ProteinCartography tool. Bottom: Representative prenol binding protein structures: protein backbone shown as grey cartoon, PLIP-determine interacting residues shown as blue sticks, and the lipid shown in orange sticks.


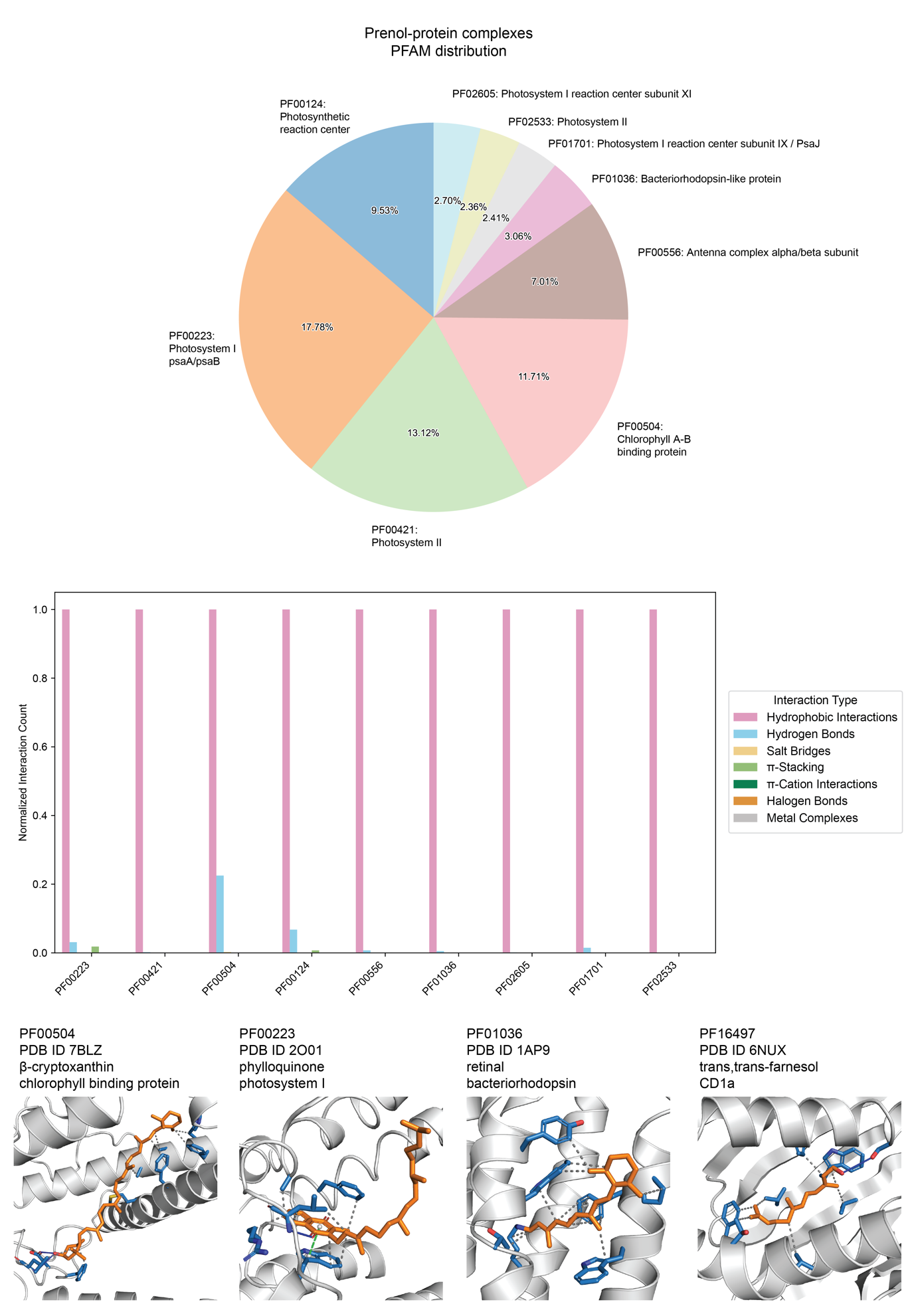


**Figure S32.** **PFAMs and interaction profiles for prenol-protein complexes.** Top: Pie chart of PFAMs associated with the most common prenol-protein complexes. The PFAM frequency values represent the number of PFAM occurrences across prenol-protein complexes in the BioDolphin database. Middle: Normalized number of interactions performed for each PFAM separately. Interaction counts were normalized by dividing counts for each interaction type by the highest count within each PFAM. Bottom: Representative examples PLIP-determined interactions in prenol-protein complexes. Protein backbone shown as grey cartoon, PLIP-determine interacting residues shown as blue sticks, and the lipid shown in orange sticks.


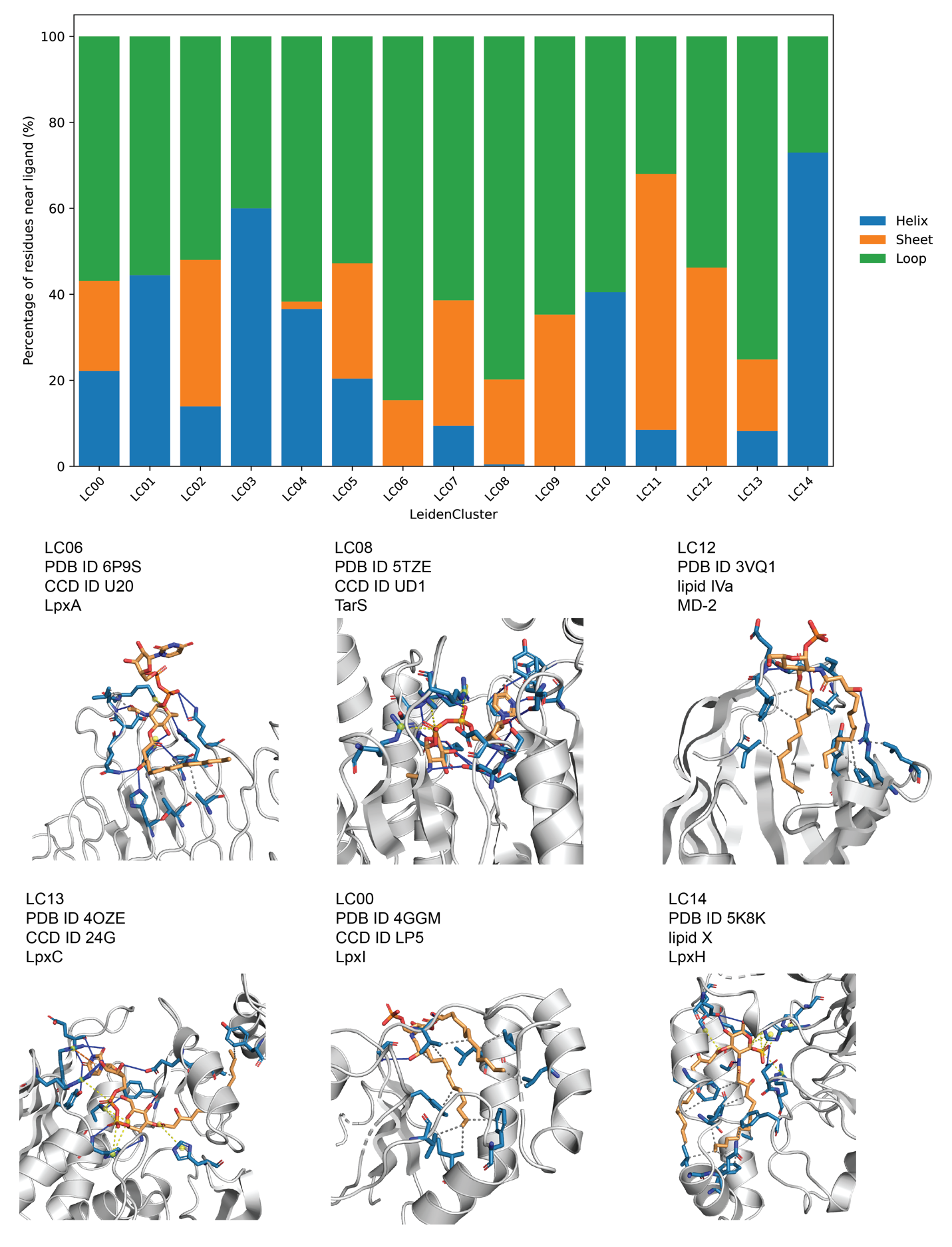


**Figure S33. Landscape of secondary structure content of saccharolipid binding protein proteins across different Leiden clusters.** Top: comparison of secondary structure content of protein residues within 5 Å of the saccharolipid ligand across different Leiden clusters derived using the ProteinCartography tool. Bottom: Representative saccharolipid binding protein structures: protein backbone shown as grey cartoon, PLIP-determine interacting residues shown as blue sticks, and the lipid shown in orange sticks.


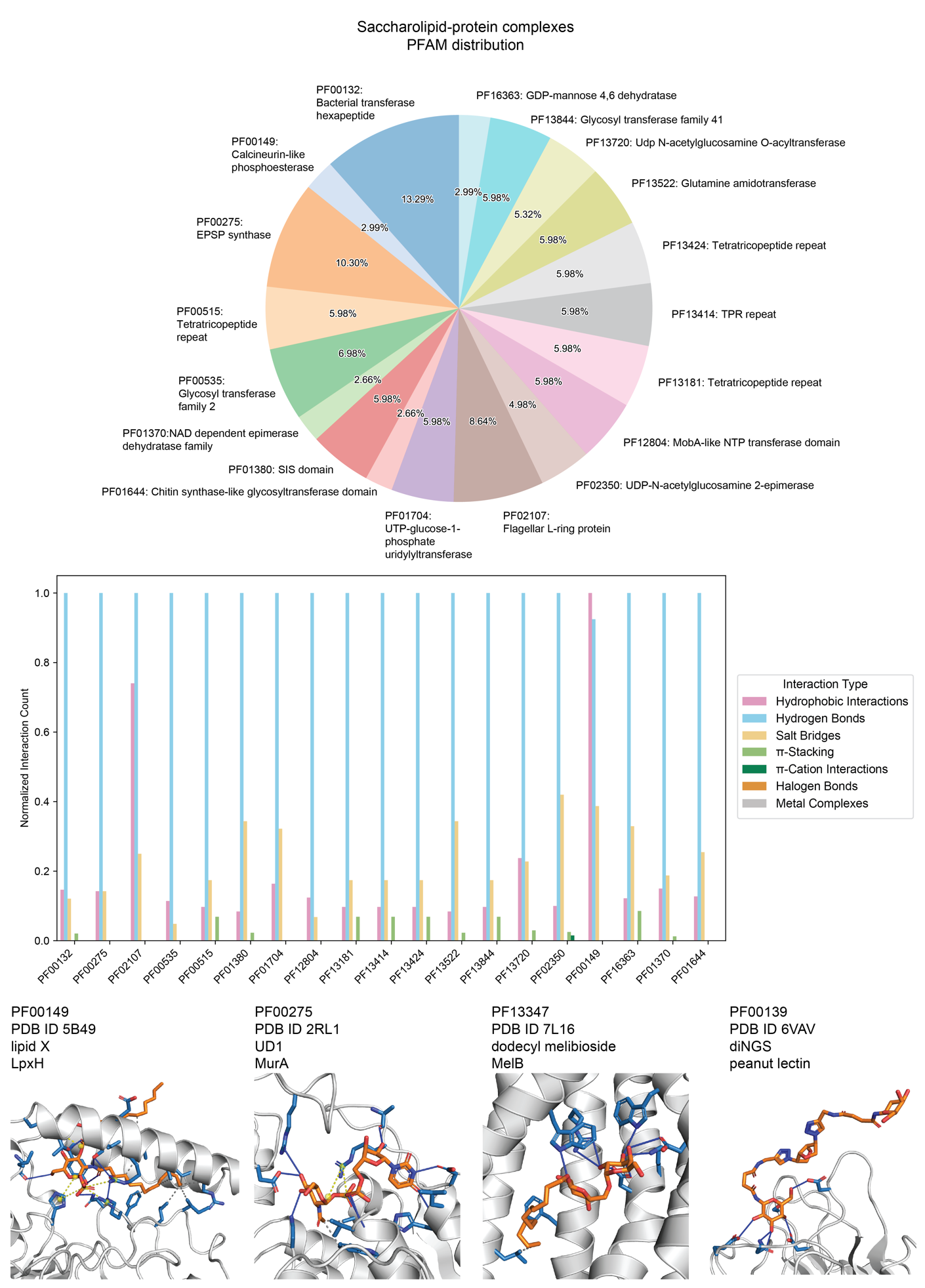


**Figure S34.** **PFAMs and interaction profiles for saccharolipid-protein complexes.** Top: Pie chart of PFAMs associated with the most common saccharolipid-protein complexes. The PFAM frequency values represent the number of PFAM occurrences across saccharolipid-protein complexes in the BioDolphin database. Middle: Normalized number of interactions performed for each PFAM separately. Interaction counts were normalized by dividing counts for each interaction type by the highest count within each PFAM. Bottom: Representative examples PLIP-determined interactions in saccharolipid-protein complexes. Protein backbone shown as grey cartoon, PLIP-determine interacting residues shown as blue sticks, and the lipid shown in orange sticks.


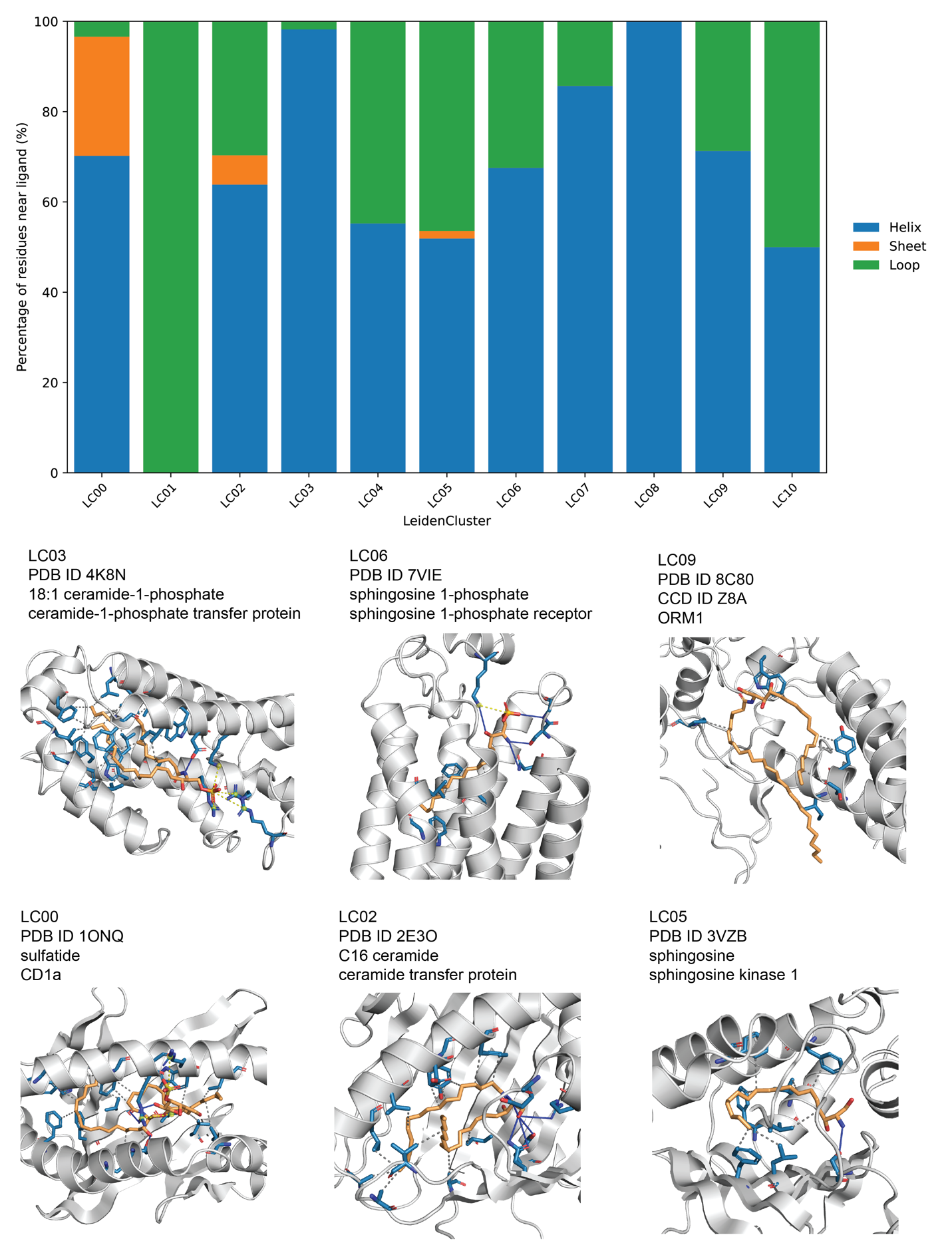


**Figure S35. Landscape of secondary structure content of sphingolipid binding protein proteins across different Leiden clusters.** Top: comparison of secondary structure content of protein residues within 5 Å of the sphingolipid ligand across different Leiden clusters derived using the ProteinCartography tool. Bottom: Representative sphingolipid binding protein structures: protein backbone shown as grey cartoon, PLIP-determine interacting residues shown as blue sticks, and the lipid shown in orange sticks.


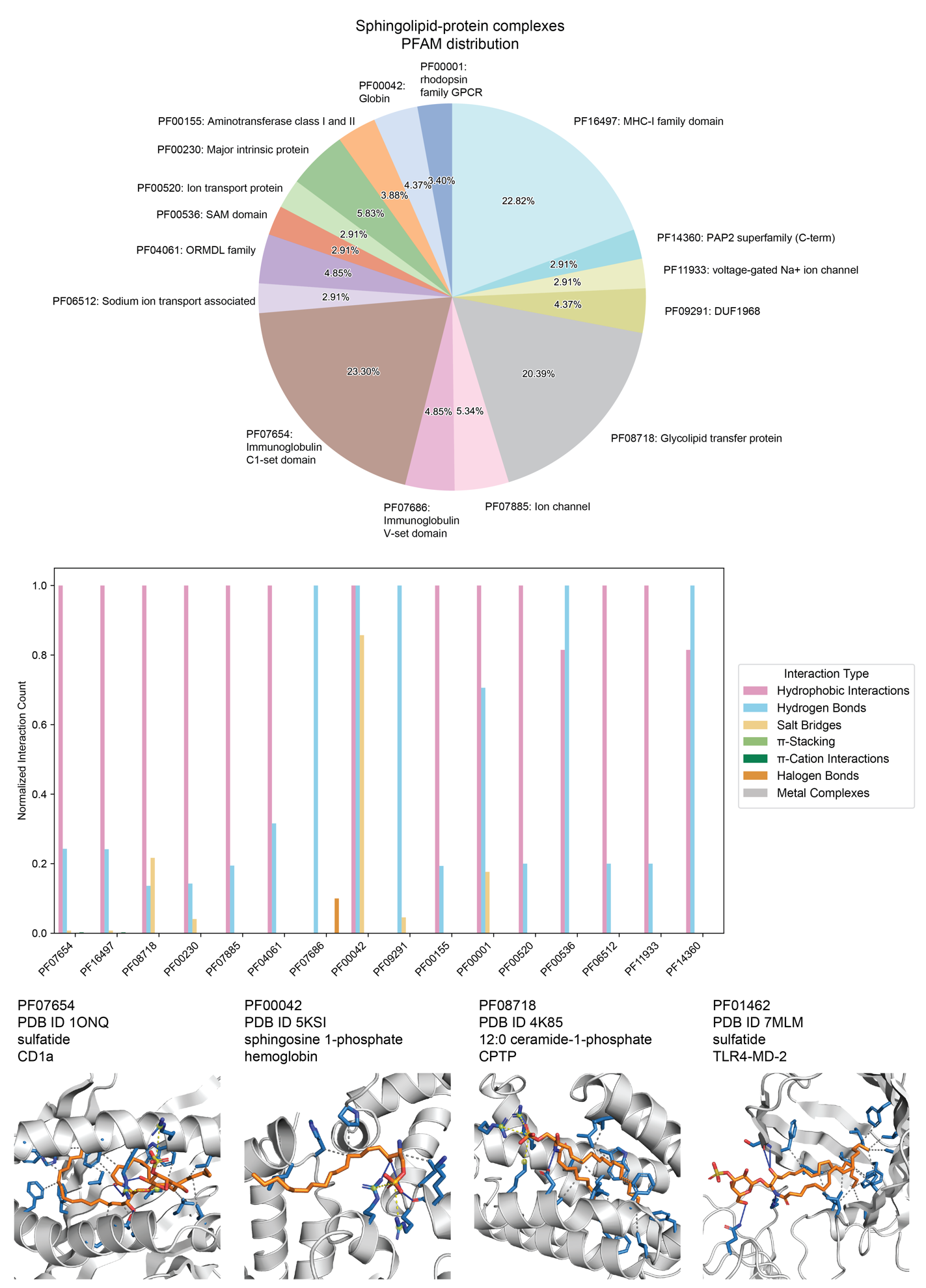


**Figure S36.** **PFAMs and interaction profiles for sphingolipid-protein complexes.** Top: Pie chart of PFAMs associated with the most common sphingolipid-protein complexes. The PFAM frequency values represent the number of PFAM occurrences across sphingolipid-protein complexes in the BioDolphin database. Middle: Normalized number of interactions performed for each PFAM separately. Interaction counts were normalized by dividing counts for each interaction type by the highest count within each PFAM. Bottom: Representative examples PLIP-determined interactions in sphingolipid-protein complexes. Protein backbone shown as grey cartoon, PLIP-determine interacting residues shown as blue sticks, and the lipid shown in orange sticks.


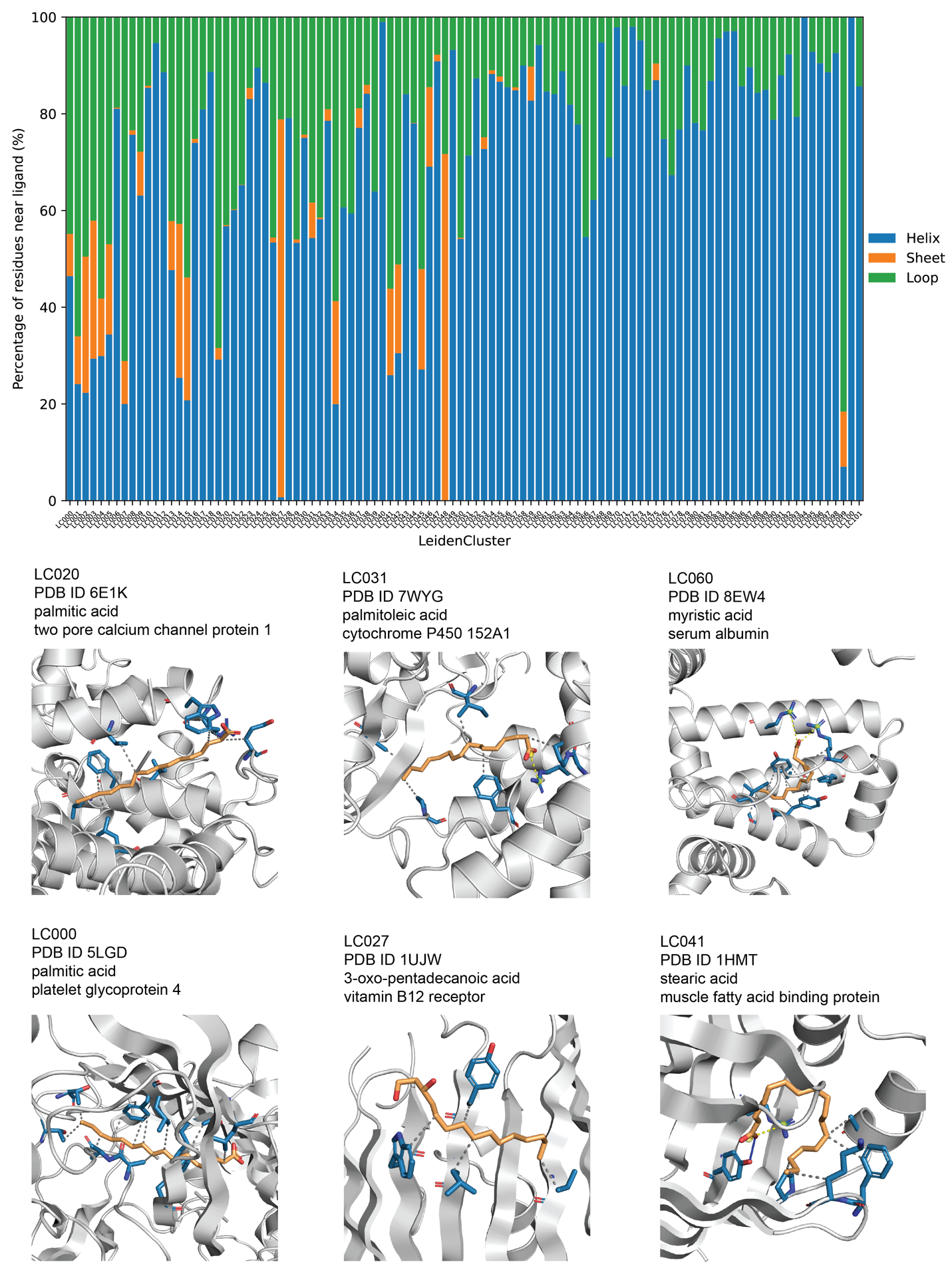


**Figure S37. Landscape of secondary structure content of fatty acyl binding protein proteins across different Leiden clusters.** Top: comparison of secondary structure content of protein residues within 5 Å of the fatty acyl ligand across different Leiden clusters derived using the ProteinCartography tool. Bottom: Representative fatty acyl binding protein structures: protein backbone shown as grey cartoon, PLIP-determine interacting residues shown as blue sticks, and the lipid shown in orange sticks.


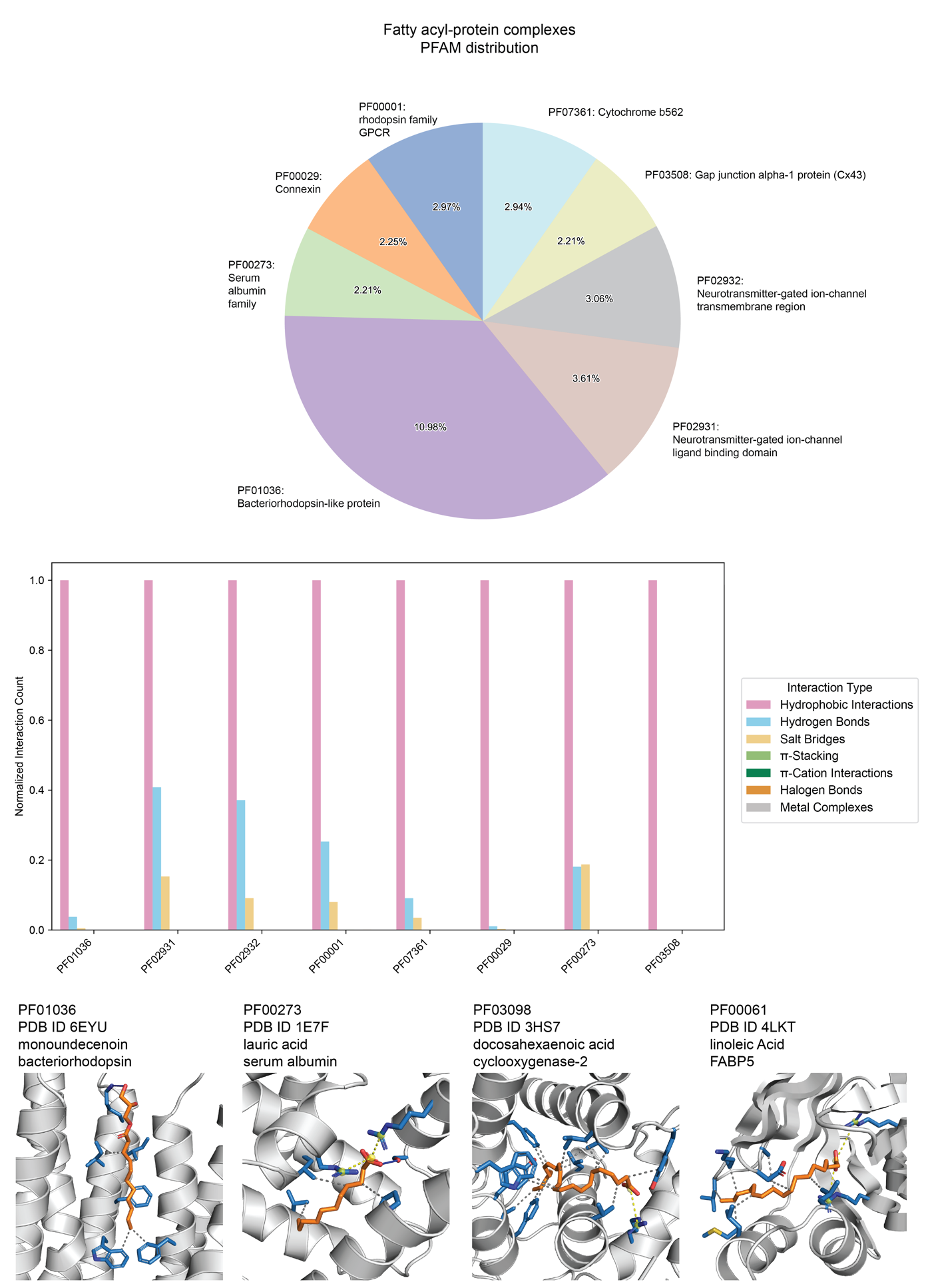


**Figure S38.** **PFAMs and interaction profiles for fatty acyl-protein complexes.** Top: Pie chart of PFAMs associated with the most common fatty acyl-protein complexes. The PFAM frequency values represent the number of PFAM occurrences across fatty acyl-protein complexes in the BioDolphin database. Middle: Normalized number of interactions performed for each PFAM separately. Interaction counts were normalized by dividing counts for each interaction type by the highest count within each PFAM. Bottom: Representative examples PLIP-determined interactions in fatty acyl-protein complexes. Protein backbone shown as grey cartoon, PLIP-determine interacting residues shown as blue sticks, and the lipid shown in orange sticks.


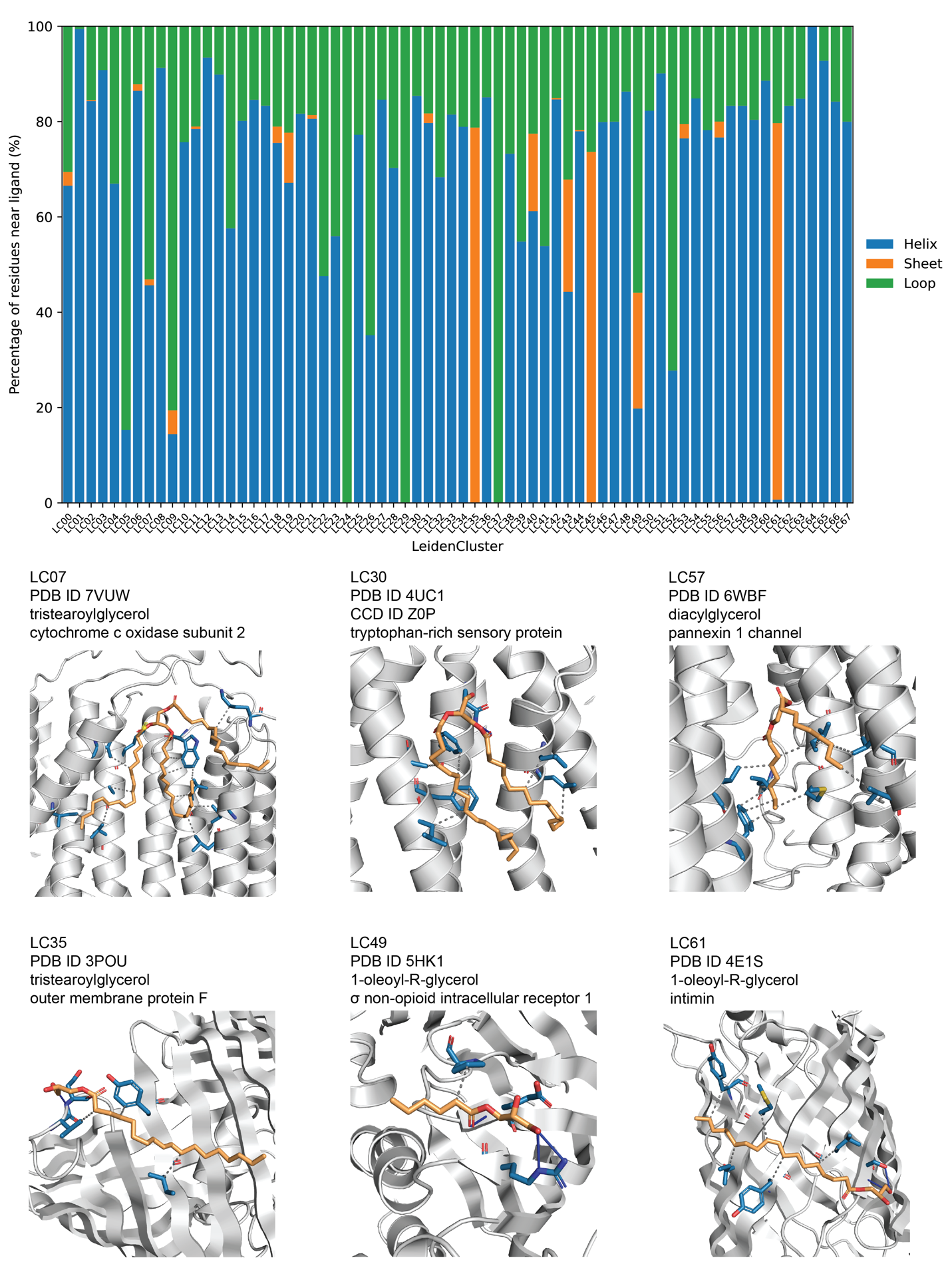


**Figure S39. Landscape of secondary structure content of glycerolipid binding protein proteins across different Leiden clusters.** Top: comparison of secondary structure content of protein residues within 5 Å of the glycerolipid ligand across different Leiden clusters derived using the ProteinCartography tool. Bottom: Representative glycerolipid binding protein structures: protein backbone shown as grey cartoon, PLIP-determine interacting residues shown as blue sticks, and the lipid shown in orange sticks.


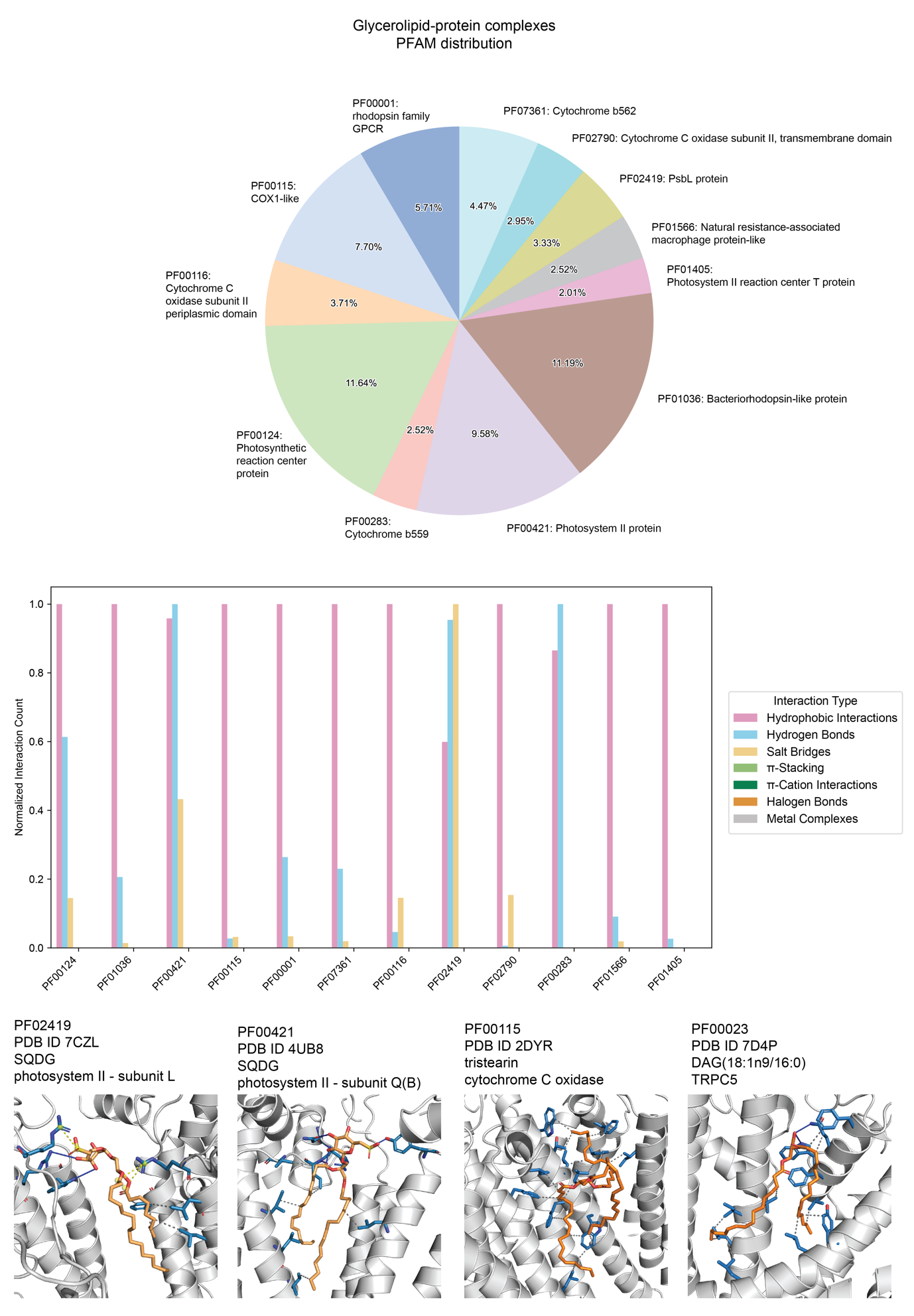


**Figure S40. PFAMs and interaction profiles for glycerolipid-protein complexes.** Top: Pie chart of PFAMs associated with the most common glycerolipid-protein complexes. The PFAM frequency values represent the number of PFAM occurrences across glycerolipid-protein complexes in the BioDolphin database. Middle: Normalized number of interactions performed for each PFAM separately. Interaction counts were normalized by dividing counts for each interaction type by the highest count within each PFAM. Bottom: Representative examples PLIP-determined interactions in glycerolipid-protein complexes. Protein backbone shown as grey cartoon, PLIP-determine interacting residues shown as blue sticks, and the lipid shown in orange sticks.


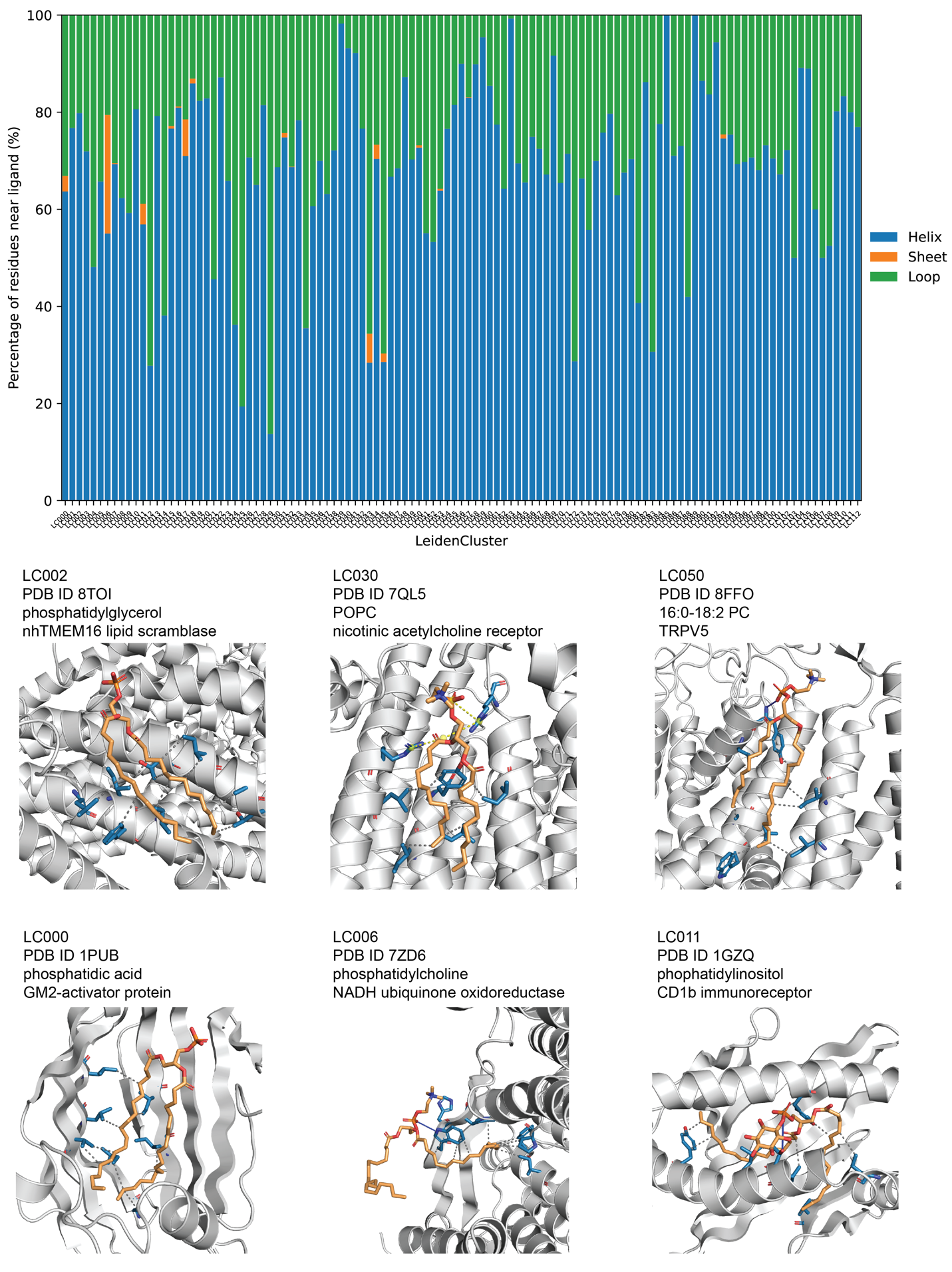


**Figure S41. Landscape of secondary structure content of glycerophospholipid binding protein proteins across different Leiden clusters.** Top: comparison of secondary structure content of protein residues within 5 Å of the glycerophospholipid ligand across different Leiden clusters derived using the ProteinCartography tool. Bottom: Representative glycerophospholipid binding protein structures: protein backbone shown as grey cartoon, PLIP-determine interacting residues shown as blue sticks, and the lipid shown in orange sticks.


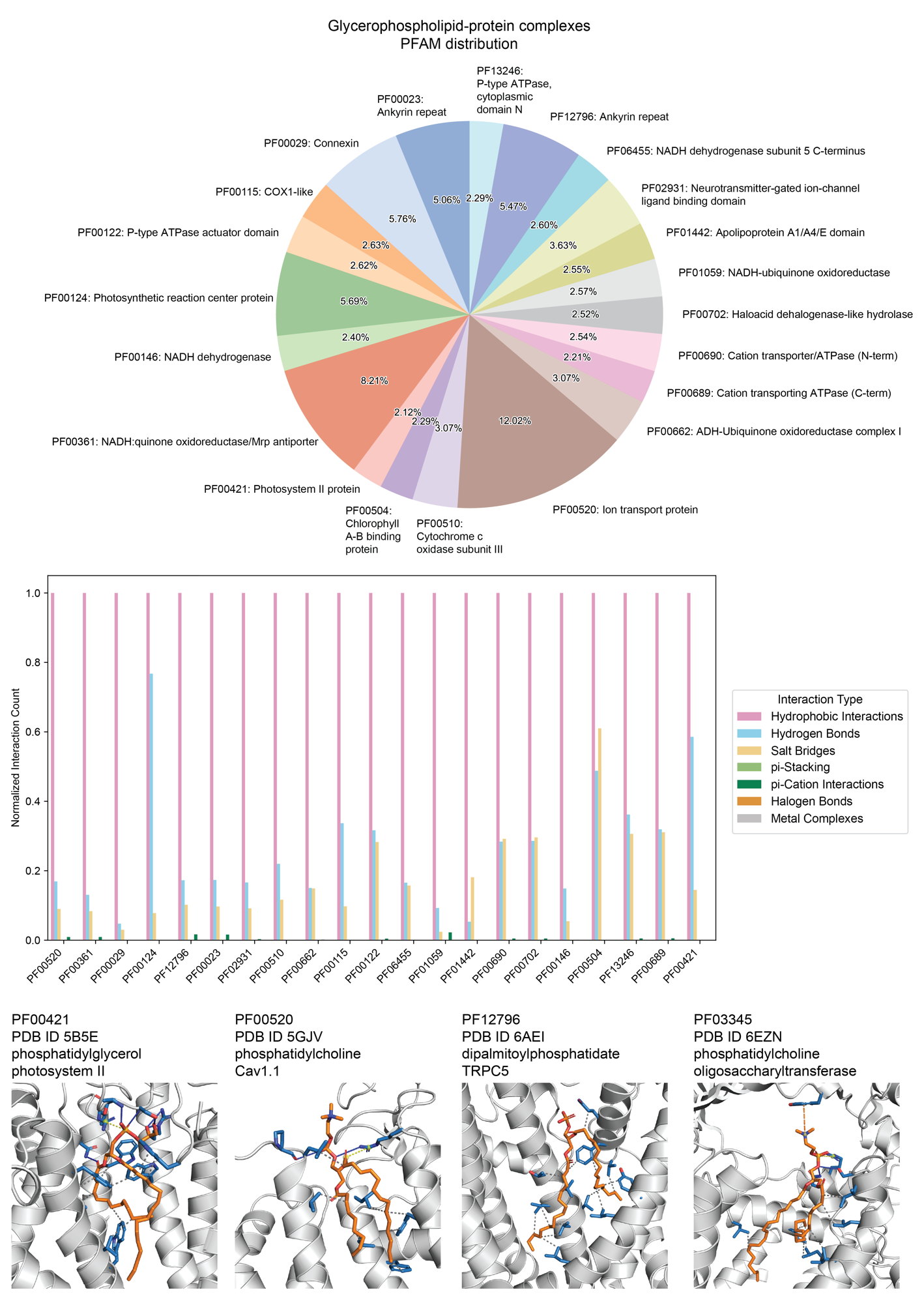


**Figure S42.** **PFAMs and interaction profiles for glycerophospholipid-protein complexes.** Top: Pie chart of PFAMs associated with the most common glycerophospholipid-protein complexes. The PFAM frequency values represent the number of PFAM occurrences across glycerophospholipid-protein complexes in the BioDolphin database. Middle: Normalized number of interactions performed for each PFAM separately. Interaction counts were normalized by dividing counts for each interaction type by the highest count within each PFAM. Bottom: Representative examples PLIP-determined interactions in glycerophospholipid-protein complexes. Protein backbone shown as grey cartoon, PLIP-determine interacting residues shown as blue sticks, and the lipid shown in orange sticks.


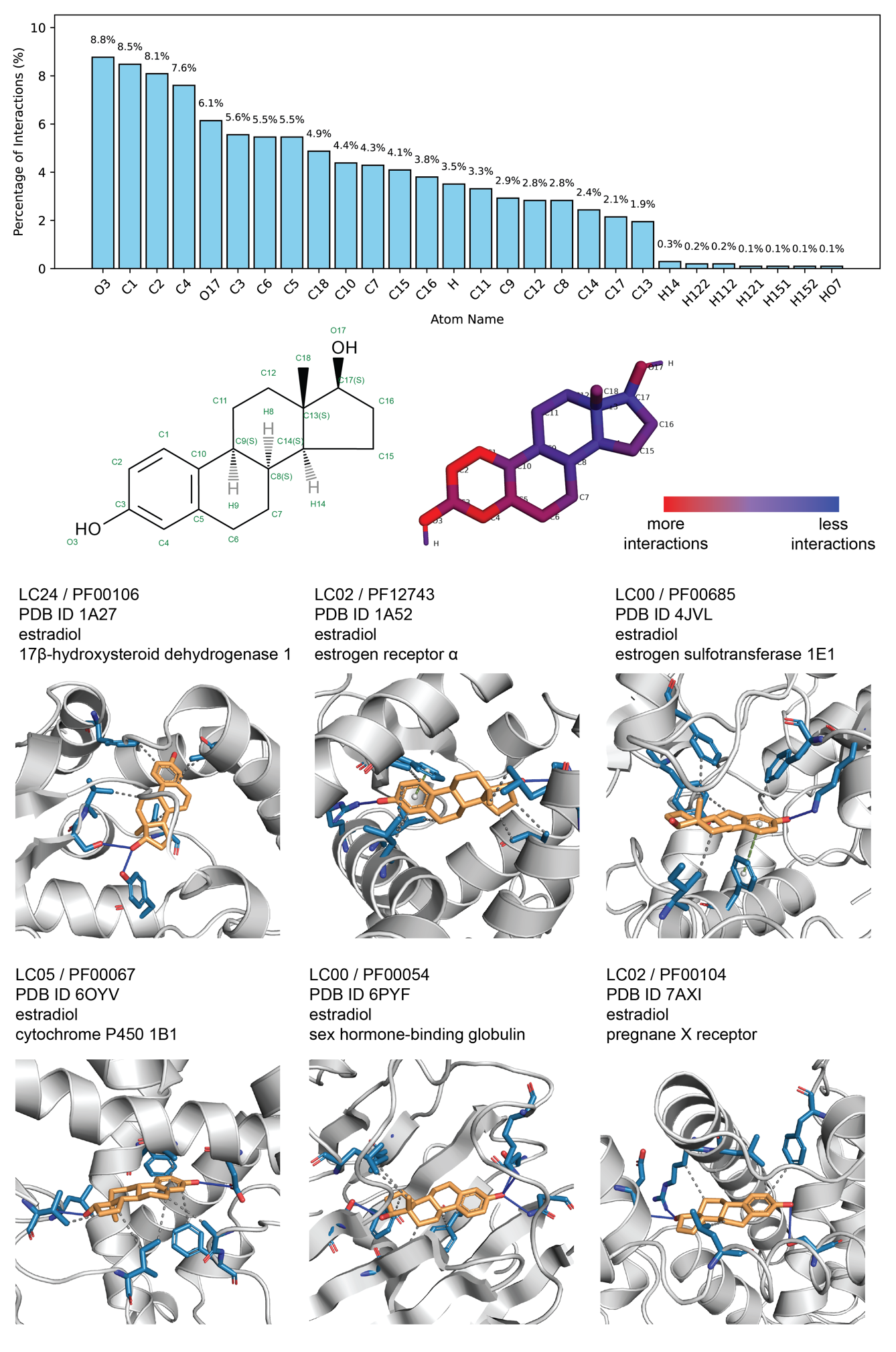


**Figure S43.** **Examples of sterol-protein interactions: different proteins with estradiol.** Representative examples PLIP-determined interactions in estradiol-protein complexes. The PDB CCD ID of estradiol is EST. Top: the percentage of interactions for each atom of EST across available all estradiol-protein complexes in the dataset. The chemical structure of EST is displayed with labeled atoms. The 3D structure of EST is colored according to interaction frequency, with atoms that interact most frequently in red and atoms that interact least in blue. Bottom: protein backbone shown as grey cartoon, PLIP-determine interacting residues shown as blue sticks, and the lipid shown in orange sticks. Leiden clusters (LC) from the ProteinCartography analysis and the PFAM values (PF) are noted.


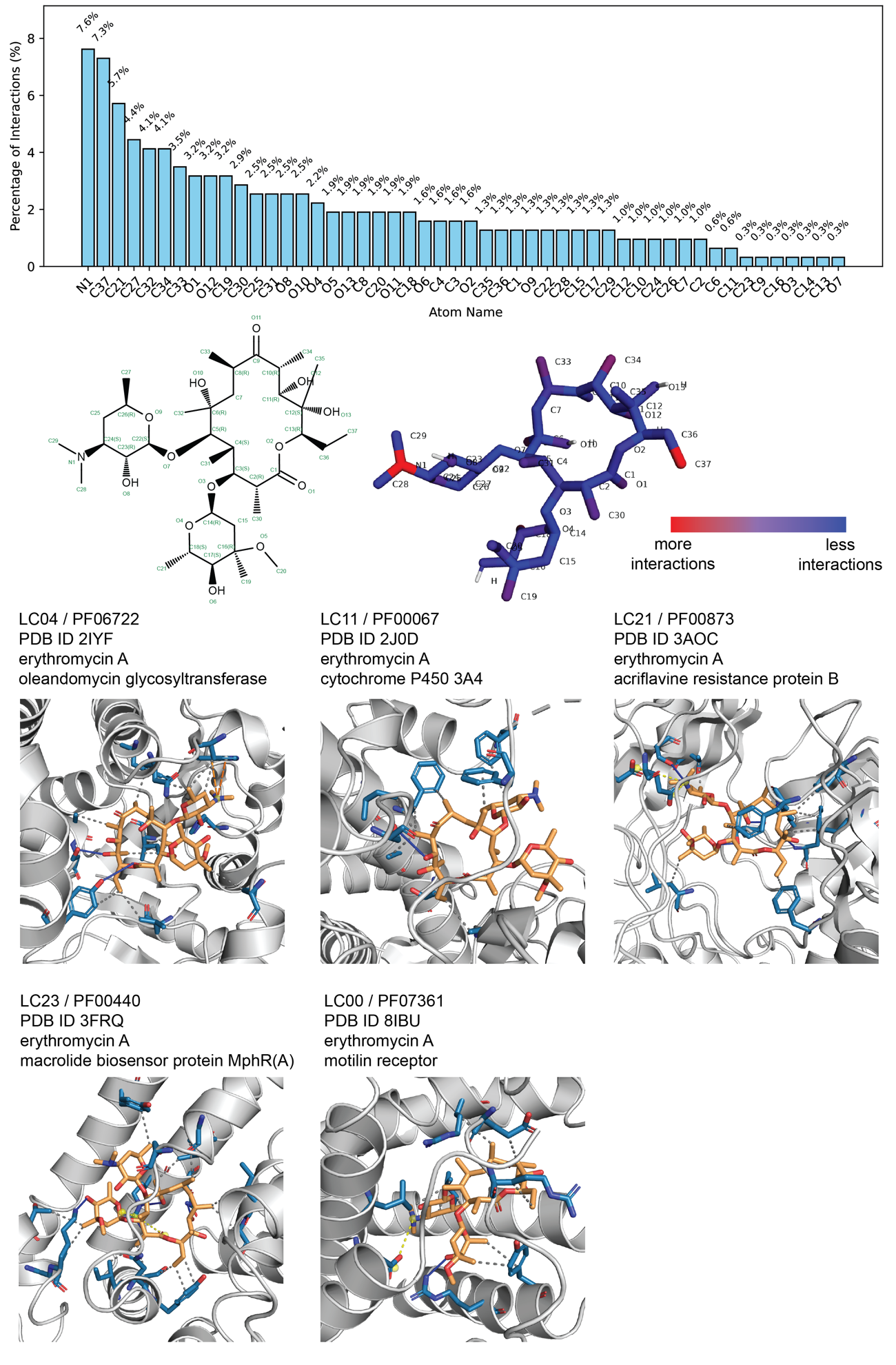


**Figure S44.** **Examples of polyketide-protein interactions: different proteins with erythromycin A.** Representative examples PLIP-determined interactions in erythromycin A-protein complexes. The PDB CCD ID of erythromycin A is ERY. Top: the percentage of interactions for each atom of ERY across available all erythromycin A-protein complexes in the dataset. The chemical structure of ERY is displayed with labeled atoms. The 3D structure of ERY is colored according to interaction frequency, with atoms that interact most frequently in red and atoms that interact least in blue. Bottom: protein backbone shown as grey cartoon, PLIP-determine interacting residues shown as blue sticks, and the lipid shown in orange sticks. Leiden clusters (LC) from the ProteinCartography analysis and the PFAM values (PF) are noted.


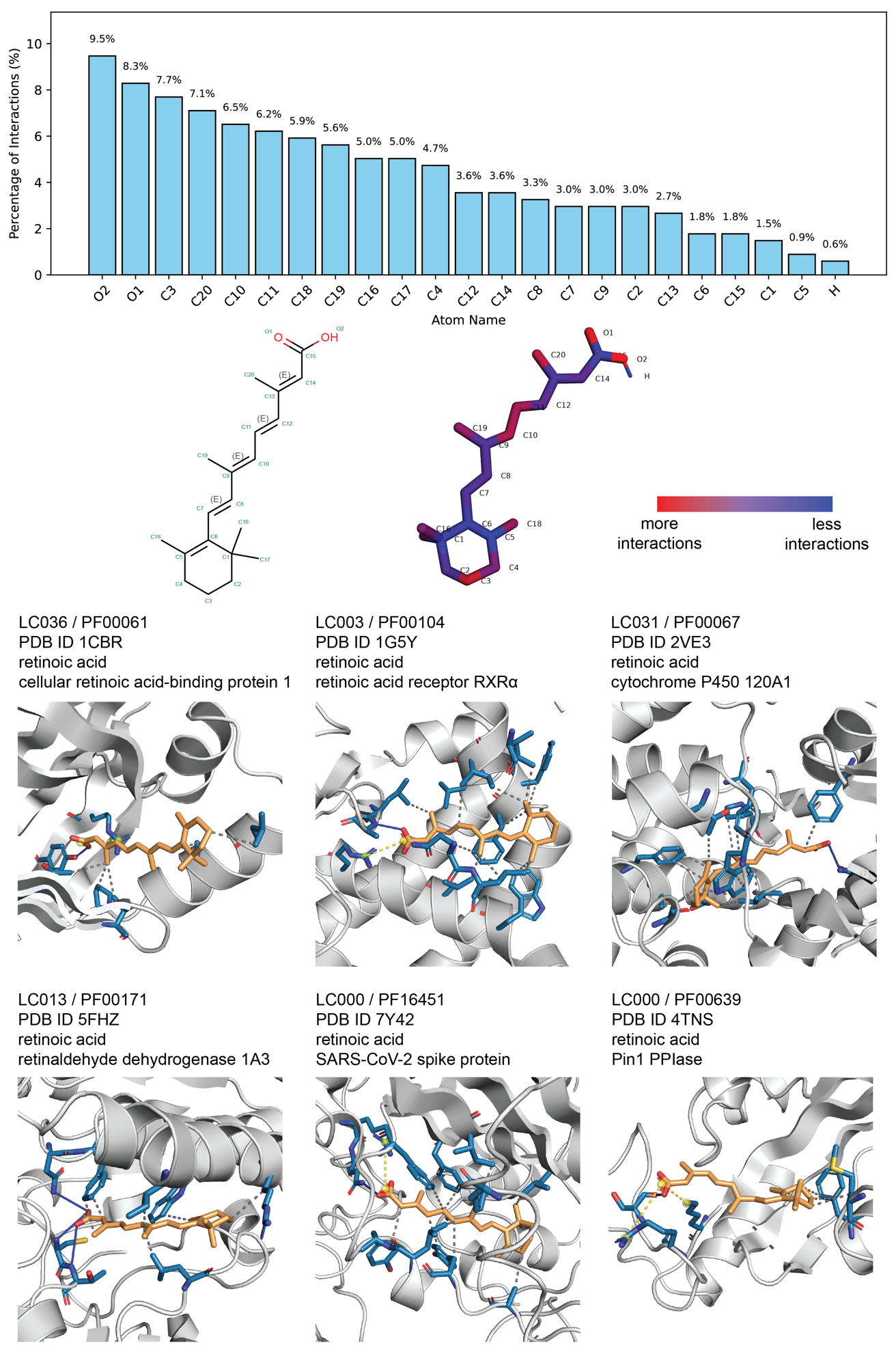


**Figure S45.** **Examples of prenol-protein interactions: different proteins with retinoic acid.** Representative examples PLIP-determined interactions in retinoic acid-protein complexes. The PDB CCD ID of retinoic acid is REA. Top: the percentage of interactions for each atom of REA across available all retinoic acid-protein complexes in the dataset. The chemical structure of REA is displayed with labeled atoms. The 3D structure of REA is colored according to interaction frequency, with atoms that interact most frequently in red and atoms that interact least in blue. Bottom: protein backbone shown as grey cartoon, PLIP-determine interacting residues shown as blue sticks, and the lipid shown in orange sticks. Leiden clusters (LC) from the ProteinCartography analysis and the PFAM values (PF) are noted.


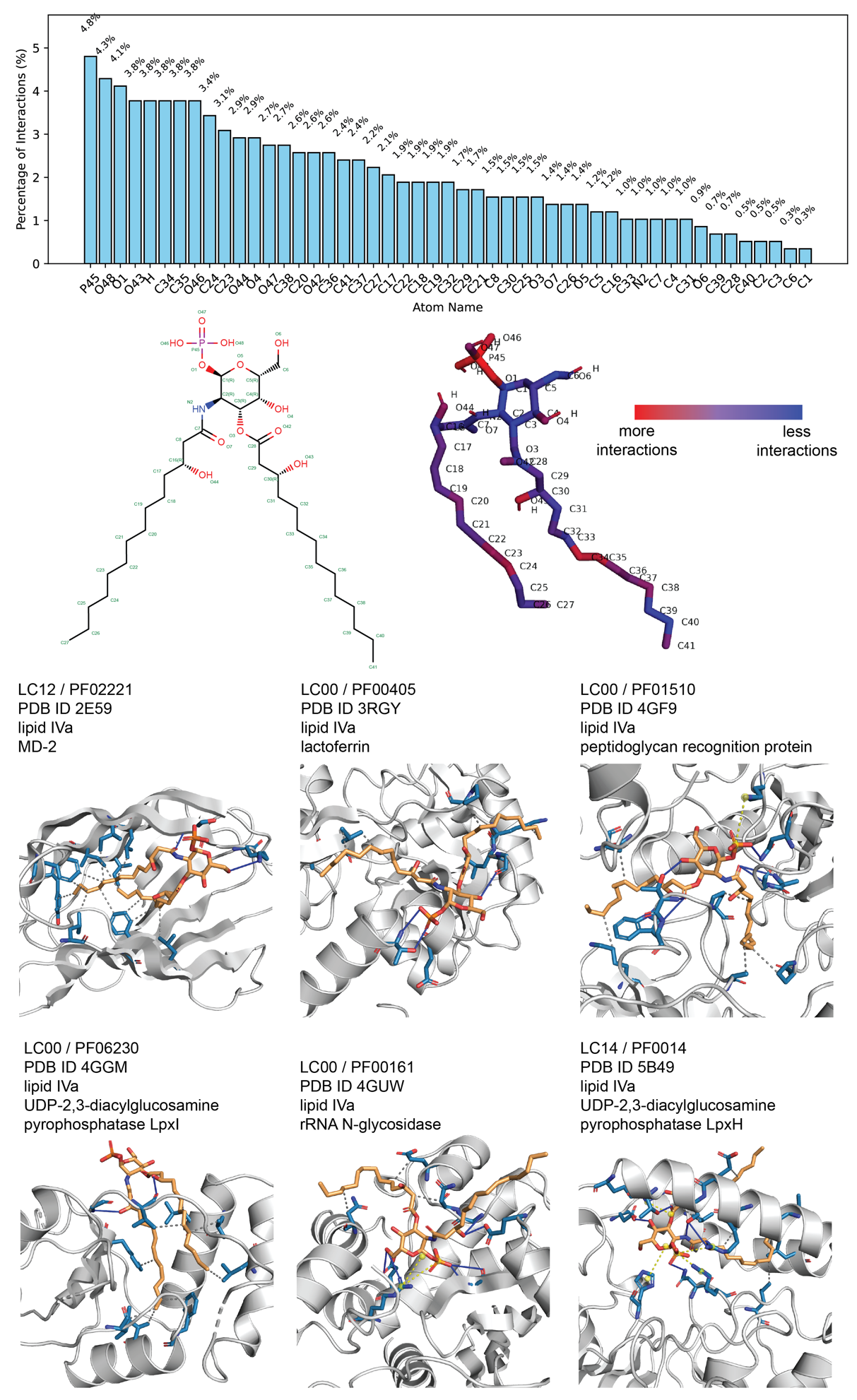


**Figure S46.** **Examples of saccharolipid-protein interactions: different proteins with lipid IVa.** Representative examples PLIP-determined interactions in lipid IVa-protein complexes. The saccharolipid lipid IVa is a precursor in the biosynthesis of bacterial LPS (lipopolysaccharide). The PDB CCD ID of lipid IVa is LP5. Top: the percentage of interactions for each atom of LP5 across available all lipid IVa-protein complexes in the dataset. The chemical structure of LP5 is displayed with labeled atoms. The 3D structure of LP5 is colored according to interaction frequency, with atoms that interact most frequently in red and atoms that interact least in blue. Bottom: protein backbone shown as grey cartoon, PLIP-determine interacting residues shown as blue sticks, and the lipid shown in orange sticks. Leiden clusters (LC) from the ProteinCartography analysis and the PFAM values (PF) are noted.


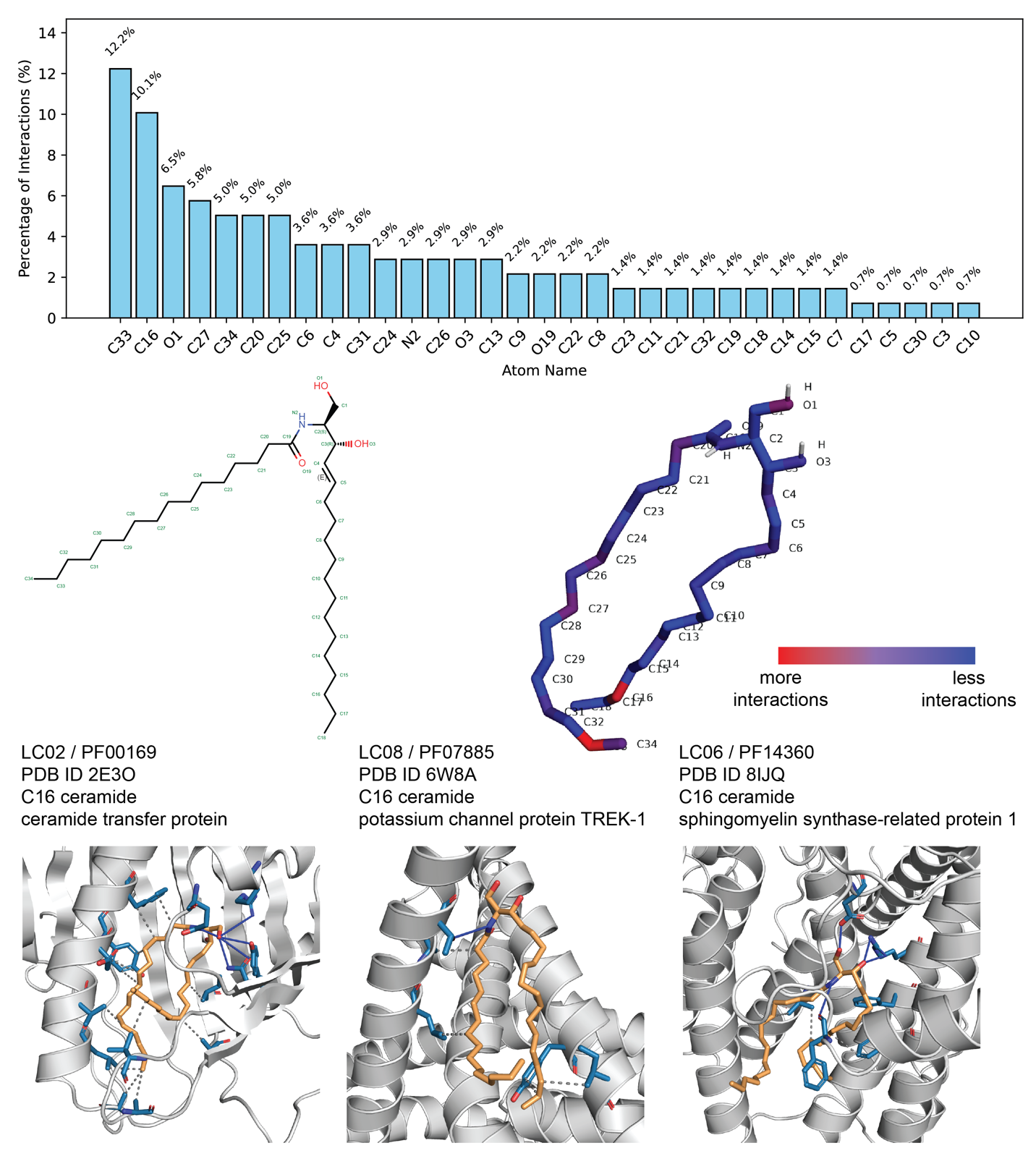


**Figure S47.** **Examples of sphingolipid-protein interactions: different proteins with C16 ceramide.** Representative examples PLIP-determined interactions in C16 ceramide-protein complexes. The PDB CCD ID of C16 ceramide is 16C. Top: the percentage of interactions for each atom of 16C across available all C16 ceramide-protein complexes in the dataset. The chemical structure of 16C is displayed with labeled atoms. The 3D structure of 16C is colored according to interaction frequency, with atoms that interact most frequently in red and atoms that interact least in blue. Bottom: protein backbone shown as grey cartoon, PLIP-determine interacting residues shown as blue sticks, and the lipid shown in orange sticks. Leiden clusters (LC) from the ProteinCartography analysis and the PFAM values (PF) are noted.


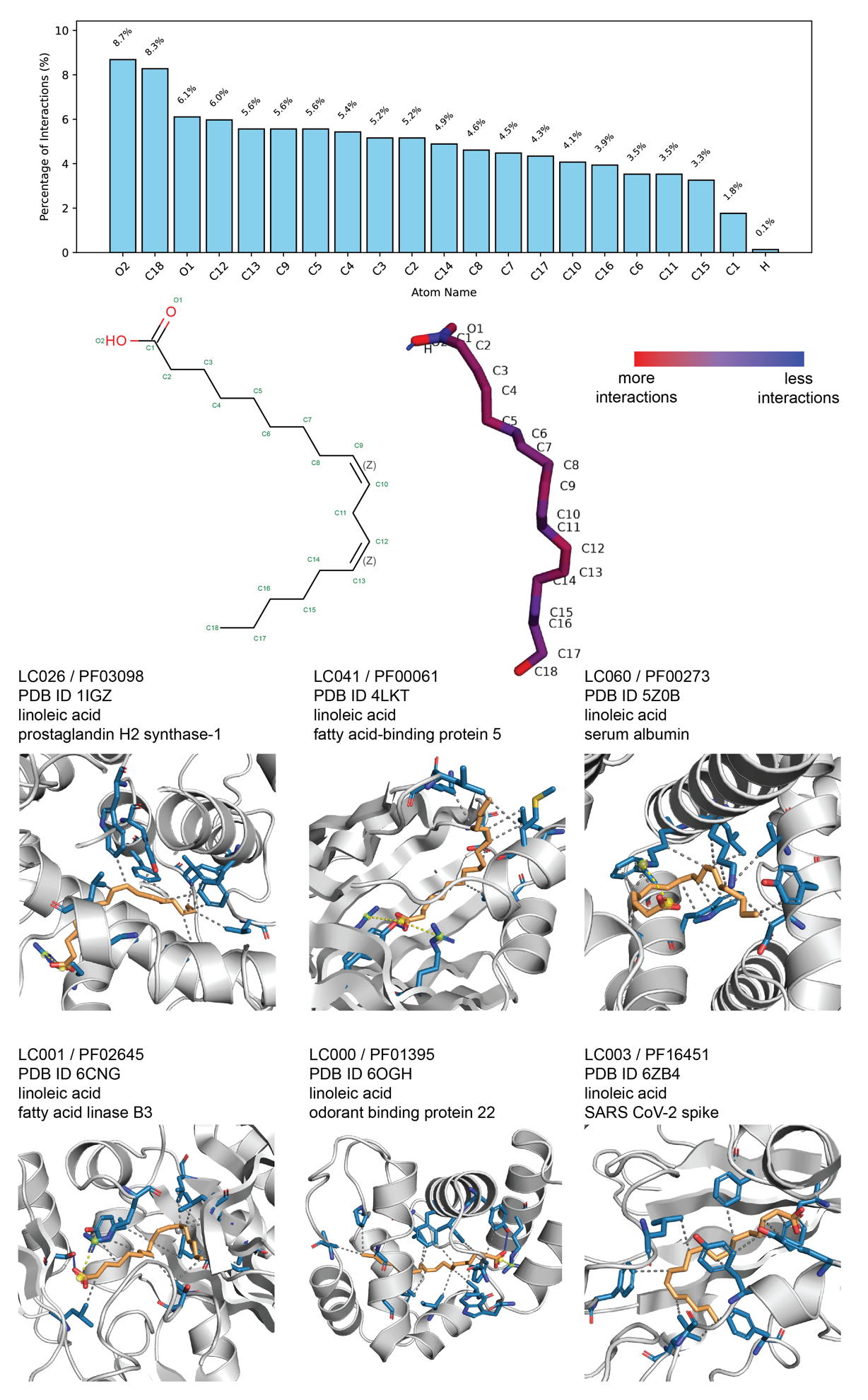


**Figure S48.** **Examples of fatty acyl-protein interactions: different proteins with linoleic acid.** Representative examples PLIP-determined interactions in linoleic acid-protein complexes. The PDB CCD ID of linoleic acid is EIC. Top: the percentage of interactions for each atom of EIC across available all linoleic acid-protein complexes in the dataset. The chemical structure of EIC is displayed with labeled atoms. The 3D structure of EIC is colored according to interaction frequency, with atoms that interact most frequently in red and atoms that interact least in blue. Bottom: protein backbone shown as grey cartoon, PLIP-determine interacting residues shown as blue sticks, and the lipid shown in orange sticks. Leiden clusters (LC) from the ProteinCartography analysis and the PFAM values (PF) are noted.


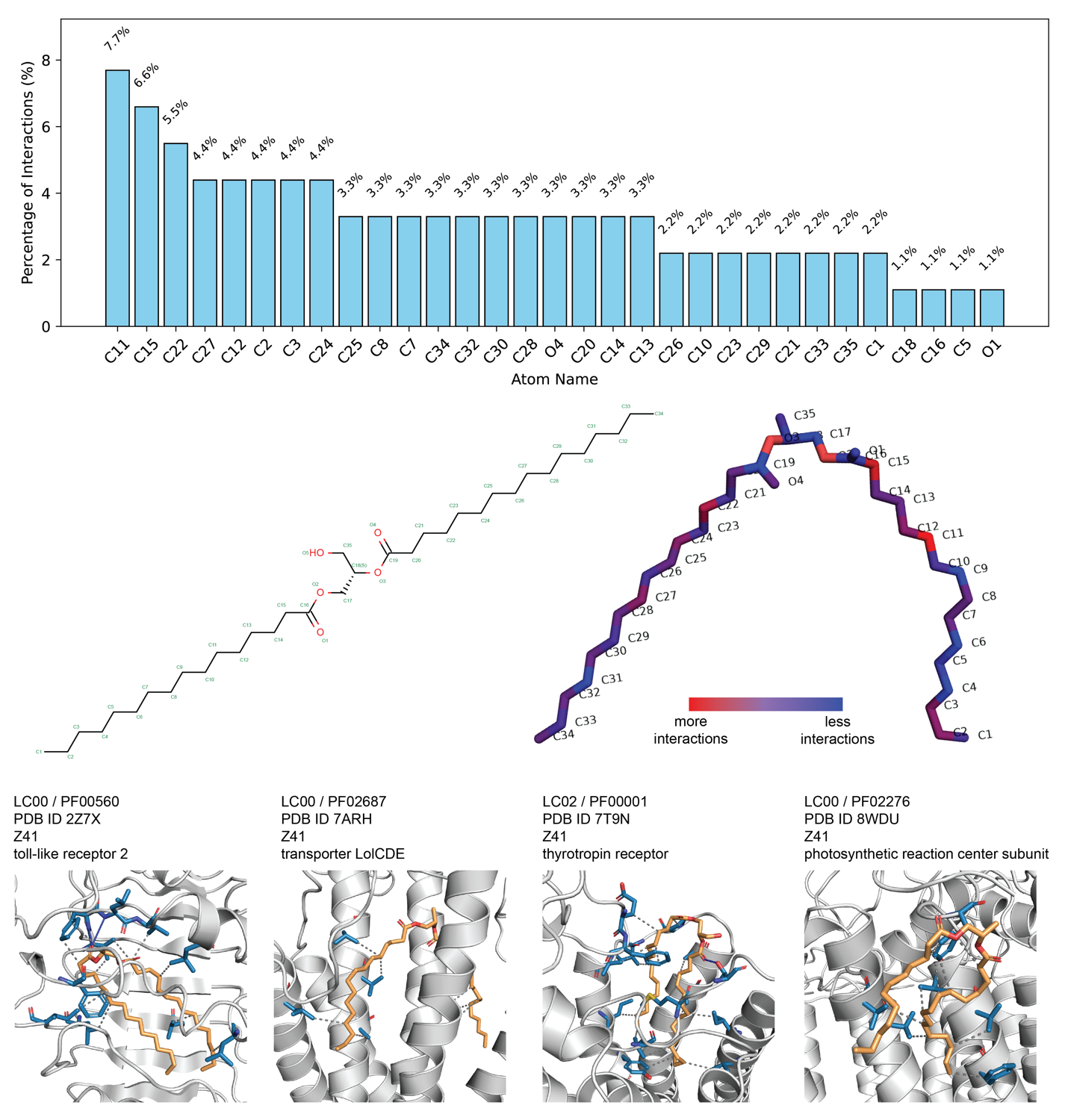


**Figure S49.** **Examples of glycerolipid-protein interactions: different proteins with (2S)-3-hydroxypropane-1,2-diyl dihexadecanoate.** Representative examples PLIP-determined interactions in (2S)-3-hydroxypropane-1,2-diyl dihexadecanoate-protein complexes. The PDB CCD ID of (2S)-3-hydroxypropane-1,2-diyl dihexadecanoate is Z41. Top: the percentage of interactions for each atom of Z41 across available all (2S)-3-hydroxypropane-1,2-diyl dihexadecanoate-protein complexes in the dataset. The chemical structure of Z41 is displayed with labeled atoms. The 3D structure of Z41 is colored according to interaction frequency, with atoms that interact most frequently in red and atoms that interact least in blue. Bottom: protein backbone shown as grey cartoon, PLIP-determine interacting residues shown as blue sticks, and the lipid shown in orange sticks. Leiden clusters (LC) from the ProteinCartography analysis and the PFAM values (PF) are noted.


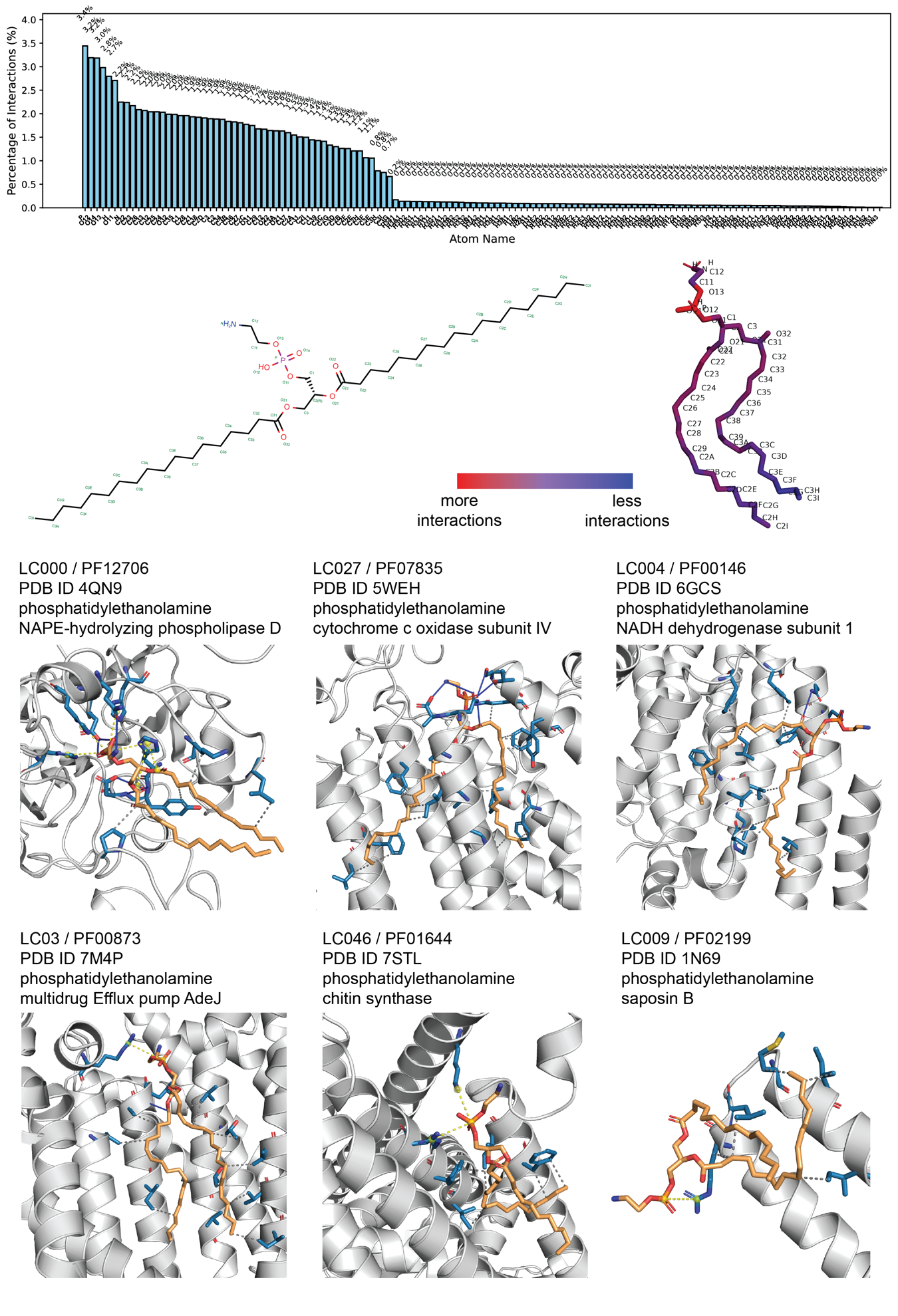


**Figure S50** **Examples of glycerophospholipid-protein interactions: different proteins with phosphatidylethanolamine (PE).** Representative examples PLIP-determined interactions in phosphatidylethanolamine-protein complexes. The PDB CCD ID of phosphatidylethanolamine is 3PE. Top: the percentage of interactions for each atom of 3PE across available all phosphatidylethanolamine-protein complexes in the dataset. The chemical structure of 3PE is displayed with labeled atoms. The 3D structure of 3PE is colored according to interaction frequency, with atoms that interact most frequently in red and atoms that interact least in blue. Bottom: protein backbone shown as grey cartoon, PLIP-determine interacting residues shown as blue sticks, and the lipid shown in orange sticks. Leiden clusters (LC) from the ProteinCartography analysis and the PFAM values (PF) are noted.
